# Simvastatin as Add-On Treatment to Escitalopram in Patients With Major Depression and Obesity: A Randomized Clinical Trial

**DOI:** 10.1001/jamapsychiatry.2025.0801

**Published:** 2025-06-04

**Authors:** Christian Otte, Woo Ri Chae, Deniz Yildirim Dogan, Dominique Piber, Stefan Roepke, An Bin Cho, Samuel Trumm, Michael Kaczmarczyk, Jelena Brasanac, Katja Wingenfeld, Stefanie Koglin, Johannes Wieditz, Klaus Junghanns, Michael Lucht, David Prvulovic, Tillmann H. C. Krüger, Jan Terock, Moritz Haaf, Tobias Hofmann, Nicole Mauche, Jan Philipp Klein, Hans Jörgen Grabe, Andreas Reif, Kai G. Kahl, Deborah Janowitz, Gregor Leicht, Kim Hinkelmann, Maria Strauß, Tim Friede, Stefan M. Gold

**Affiliations:** 1 Charité—Universitätsmedizin Berlin, corporate member of Freie Universität Berlin, Department of Psychiatry and Psychotherapy, Humboldt-Universität zu Berlin, Berlin, Germany; 2 German Center for Mental Health partner site Berlin/Potsdam, Germany; 3 Berlin Institute of Health at Charité Universitätsmedizin Berlin, Berlin, Germany; 4 Department of Medical Statistics, University Medical Center Göttingen, Göttingen, Germany; 5 Department of Psychiatry and Psychotherapy, Lübeck University, Lübeck, Germany; 6 Department of Psychiatry and Psychotherapy, University Medicine Greifswald, Greifswald, Germany; 7 Department of Psychiatry, Psychosomatic Medicine and Psychotherapy, University Medical Centre Frankfurt, Frankfurt am Main, Germany; 8 Department of Psychiatry, Social Psychiatry and Psychotherapy, Hannover Medical School, Hanover, Germany; 9 Centre for Systems Neuroscience Hannover, Hanover, Germany; 10 Department of Psychiatry and Psychotherapy, Helios Hanseklinikum Stralsund, Stralsund, Germany; 11 Department of Psychiatry and Psychotherapy, University Medical Center Hamburg-Eppendorf, Hamburg, Germany; 12 Charité—Universitätsmedizin Berlin, corporate member of Freie Universität Berlin, Department of Psychosomatic Medicine, Center for Internal Medicine and Dermatology, Humboldt-Universität zu Berlin, Berlin, Germany; 13 Charité—Universitätsmedizin Berlin, Center for Patient-Centered Outcomes Research, Berlin, Germany; 14 Department of Psychosomatic Medicine and Psychotherapy, DRK Kliniken Berlin Wiegmann Klinik, Berlin, Germany; 15 Department of Psychiatry and Psychotherapy, University of Leipzig Medical Center, Leipzig, Germany; 16 Fraunhofer Institute for Translational Medicine and Pharmacology ITMP, Frankfurt am Main, Germany; 17 Institute of Neuroimmunology and Multiple Sclerosis, Center for Molecular Neurobiology, University Medical Center Hamburg-Eppendorf, Hamburg, Germany

## Abstract

**Importance:**

Major depressive disorder (MDD) and obesity are common noncommunicable disorders associated with substantial disease burden, which frequently occur comorbidly. Intriguingly, converging lines of evidence from animal models and genetic and observational studies have suggested a biological link between obesity, metabolic syndrome, and depression. Several small randomized clinical trials (RCTs) have suggested the antidepressive potential of statins.

**Objective:**

To examine whether simvastatin added to escitalopram is efficacious in improving depressive symptoms compared with add-on placebo.

**Design, Setting, and Participants:**

This was a confirmatory, double-blind, placebo-controlled, multicenter RCT. Adults with MDD and comorbid obesity from 9 tertiary care settings in Germany were enrolled in this analysis. Data were analyzed from July to October 2024.

**Interventions:**

Simvastatin (40 mg per day) or placebo as add-on to escitalopram (10 mg for the first 2 weeks, then increased to 20 mg until the end of study) in a double-blind fashion for 12 weeks.

**Main Outcomes and Measures:**

The primary outcome was change in Montgomery-Åsberg Depression Rating Scale (MADRS) score from baseline (week 0) to week 12.

**Results:**

From August 21, 2020, to June 06, 2024, a total of 161 patients were enrolled at 9 sites in Germany, of which 160 patients were included in the intention-to-treat analysis (placebo: n = 79, simvastatin: n = 81; mean [SD] age, 39.0 [11.0] years; 126 female [79%]). Retention in the trial was excellent (95.6%), and blinding was effectively maintained. There were 4 serious adverse events with no difference between the groups. Primary end point analysis in the intention-to-treat sample showed no significant treatment effect of add-on simvastatin in MADRS scores (mixed models for repeated measures least squares mean difference, 0.47 points; 95% CI, −2.08 to 3.02; *P* = .71). No effects of simvastatin treatment were observed in any of the mental health–related secondary end points. However, simvastatin treatment significantly reduced low-density lipoprotein cholesterol (simvastatin, −40.37 mg/dL; 95% CI, −47.41 to −33.33 mg/dL; placebo, −3.78 mg/dL; 95% CI, −11.18 to 3.62 mg/dL; *P* < .001), total cholesterol (simvastatin, −39.07 mg/dL; 95% CI, −49.42 to −28.73 mg/dL; placebo, −4.89 mg/dL; 95% CI, −15.64 to 5.87 mg/dL; *P* < .001), and C-reactive protein (simvastatin, −1.04 mg/L; 95% CI, −1.89 to −0.20 mg/L; placebo, 0.57 mg/L; 95% CI, −0.28 to 1.42 mg/L; *P* = .003) compared with placebo.

**Conclusions and Relevance:**

The study failed to meet its primary end point. This demonstrates that simvastatin did not exert additional antidepressive effects when added to escitalopram in patients with comorbid MDD and obesity, despite improving the cardiovascular risk profile.

**Trial Registration:**

ClinicalTrials.gov Identifier: NCT04301271

## Key Points

**Question:**

Does add-on simvastatin, 40 mg per day, to the selective serotonin reuptake inhibitor escitalopram (20 mg per day) improve depression to a greater extent than add-on placebo in patients with major depressive disorder (MDD) and comorbid obesity?

**Findings:**

In this randomized clinical trial including 161 participants with comorbid MDD and obesity, no antidepressive effects were found for simvastatin, 40 mg per day, in the primary end point (change from baseline in Montgomery-Åsberg Depression Rating Scale scores) or any key secondary end point. However, simvastatin reduced low-density lipoprotein cholesterol, total cholesterol, and C-reactive protein levels.

**Meaning:**

Study results demonstrate that simvastatin did not exert additional antidepressive effects when added to escitalopram in patients with comorbid MDD and obesity, despite improving the cardiovascular risk profile.

## Teaser

This randomized clinical trial investigates if adding simvastatin to escitalopram improves depression in patients with major depressive disorder and comorbid obesity.

## Introduction

Major depressive disorder (MDD) is 1 of the top 2 leading causes of burden of disease^1,2^ with a lifetime prevalence of approximately 1 in 6.^3^ Obesity also constitutes a major public health concern, with 1 of 8 people worldwide currently meeting diagnostic criteria for obesity.^4^ MDD and obesity frequently co-occur,^5^ and the presence of one condition increases the risk for developing the other by approximately 50% to 60%.^6^ MDD and obesity are both linked to numerous chronic medical conditions and premature mortality.^7,8^ Thus, evidence-based guidance for better treatment approaches to comorbid depression and obesity is urgently needed.

With regard to treatment, statins (3-hydroxy-3-methylglutaryl coenzyme A [HMG CoA] reductase inhibitors) may have antidepressive effects: a number of experimental studies have shown that statins—in addition to their lipid-lowering properties—may exert pleiotropic effects with potential relevance for depression pathobiology (eg, targeting inflammation, neurogenesis, stress hormones, and relevant neurotransmitter systems^7^). Although earlier meta-analyses^8,9^ of cohort studies on the link between statin prescriptions and depression have yielded conflicting results, recent analyses^10,11,12^ of large-scale cohort and registry data have suggested that statin use may be linked to a lower risk of depression.

Given the methodological limitations of observational studies to derive causal interpretations of treatment effects, randomized clinical trials (RCTs) are needed to test the clinical efficacy of statins in MDD. Indeed, several small RCTs^13,14,15,16,17^ have suggested that statins, when added to standard antidepressants, can reduce depression severity in MDD. Some of these trials^18,19,20^ reported large effects and, consequently, several meta-analyses of the literature supported the potential of statins as antidepressants. However, more recent, large, well-conducted RCTs^21,22^ failed to show benefits of statins compared with placebo when added to standard care.

Moreover, to our knowledge, no RCT thus far has tested the antidepressive potential of statins in patients with MDD and comorbid obesity. Importantly, this is a difficult-to-treat population that often exhibits a chronic course of MDD and is more likely to show treatment resistance to standard pharmacological therapy.^23^ We, therefore, conducted an RCT of add-on simvastatin treatment to the standard antidepressant escitalopram in patients with comorbid MDD and obesity. We hypothesized that add-on simvastatin would improve depression to a greater extent than add-on placebo.

## Methods

### Study Design

This was a 2-arm, double-blind, placebo-controlled multicenter RCT conducted in 9 academic medical centers in Germany. The trial and its protocol (Supplement 1) were reviewed and approved by the ethics committee of the federal state of Berlin and by the federal regulatory authority (Bundesinstitut für Arzneimittel und Medizinprodukte). This trial followed the Consolidated Standards of Reporting Trials (CONSORT) reporting guidelines.

### Participants

The main inclusion criteria for the trial were as follows: (1) written informed consent, (2) major depressive episode according to the *DSM-5*, (3) Montgomery-Åsberg Depression Rating Scale (MADRS) score of 18 or greater, (4) body mass index (BMI) of 30 or greater (calculated as weight in kilograms divided by height in meters squared), and (5) age between 18 and 65 years (≥18 and ≤65). Main exclusion criteria were as follows: (1) current use of statins, (2) current use of antidepressants, (3) acute suicidal ideation (MADRS item 10 >4), (4) pregnancy, or breastfeeding or female individuals with childbearing potential without an acceptable form of contraception (defined as Pearl index <1), and (5) current use of psychotropic medication (eg, antipsychotics, anticonvulsants, lithium, or St John’s wort) except for benzodiazepines, nonbenzodiazepines, and opiates. The eMethods in Supplement 2 contains a complete list of inclusion and exclusion criteria. Information on participant race and ethnicity was not collected for this study because collecting such data is heavily regulated and restricted under German data protection laws.

### Randomization and Masking

Participants of the trial were randomized to either receive simvastatin or placebo, both as add-on to standard antidepressant medication (escitalopram, provided open label by the trial pharmacy). Concealed allocation was ensured by an independent central pharmacy using a computer-generated randomization sequence. Based on the randomization codes, the central pharmacy provided sequentially numbered, tamper-proof containers, which were equal in weight and indistinguishable in appearance containing simvastatin or placebo. Participants were randomized in a 1:1 ratio between placebo and simvastatin using a permuted block procedure and stratified by study site.

### Procedures

A screening visit was conducted to assess eligibility, obtain informed consent, and collect demographic data. The evaluation included determining the severity of the depressive episode, recording relevant clinical variables (such as duration of the current episode, number of prior episodes, and previous treatments), and reviewing medical history. Additionally, a physical examination was performed, alongside the MINI International Neuropsychiatric Interview, electrocardiogram (ECG), and safety laboratory assessments, which included a pregnancy test for female individuals and drug screening.

At the baseline visit (week 0), body weight, height, and waist circumference were measured. Safety assessments (vital signs, ECG, and blood sample collection) and outcome measures were evaluated at baseline and at follow-up visits scheduled for weeks 1, 2, 4, 8, and 12. The trial protocol (Supplement 1) contains further details.

### Interventions

Patients were allocated to treatment with either simvastatin (40 mg per day) or placebo as add-on to escitalopram (10 mg for the first 2 weeks, then increased to 20 mg until the end of study) in a double-blind fashion for 12 weeks. In case of tolerability issues, the escitalopram dosage could be increased later than 2 weeks or could be reduced again if the dosage of 20 mg was associated with adverse effects.

### Outcomes

The change in MADRS^24^ score from baseline (week 0) to week 12 was the predefined primary outcome.

As a key secondary outcome, we assessed changes in patients’ self-reported Beck Depression Inventory II (BDI-II) scores.^21^ Additional secondary outcomes included response rates, defined as a 50% or greater reduction in MADRS scores from baseline, remission rates (MADRS score <10), and the minimal clinically important difference (MCID) based on BDI-II, defined as a greater than 17.5% change from baseline.

Exploratory outcomes included changes from baseline in the Clinical Global Impression Scale for Severity (CGI-S) and Improvement (CGI-I),^25^ the Patient Global Impression of Change (PGIC),^25^ social functioning assessed by the Social and Occupational Functioning Assessment Scale (SOFAS),^26^ and quality of life measured by the EuroQol-5 Dimensions-3 Levels (EQ-5D-3L) Questionnaire, with a calculated MCID.^27^ Additional exploratory outcomes included laboratory parameters such as high-density lipoprotein (HDL) cholesterol, low-density lipoprotein (LDL) cholesterol, total cholesterol , and high-sensitivity C-reactive protein (CRP) levels.

### Sample Size Calculation

Based on a meta-analysis^28^ of 3 small pilot RCTs in patients with depression, which reported a standardized mean difference of −0.73 (95% CI, −1.04 to −0.42; *P* < .001) between add-on statins and add-on placebo, we calculated that a sample size of 64 patients per group would achieve greater than 80% power to detect a difference in mean changes in MADRS scores from baseline to week 12 using a 2-sample *t* test at a 2-sided significance level of 5%. To account for an anticipated dropout rate of approximately 20%, we planned to recruit 80 patients per group, resulting in a total of 160 participants. More details are available in the published study protocol.^22^

### Updated Systematic Review and Meta-Analysis

For evidence synthesis of our current results with the literature, we performed an updated systematic review and meta-analysis of RCTs examining the efficacy of statins (Anatomical Therapeutic Chemical group C10AA HMG CoA reductase inhibitors; any product or dose) as monotherapy or add-on to antidepressants/standard of care and compared with placebo in adults with MDD. The protocol was prospectively registered with PROSPERO (CRD42024588640) on September 10, 2024 (eMethods in Supplement 2).

### Statistical Analysis

Details of the statistical analyses are provided in the statistical analysis plan (Supplement 1) and the eMethods in Supplement 2. In brief, efficacy analyses used the full analysis set based on the intention-to-treat (ITT) principle, including all randomized patients with at least 1 postbaseline (after week 0) data point for the primary end point. Safety analyses were performed on the safety set, comprising all randomized patients, based on the treatment received and timing of intervention. Sensitivity analyses were conducted in the ITT population, using multiple imputation and last observation carried forward.

The primary analysis compared MADRS changes from baseline (week 0) with week 12 between the simvastatin and placebo group by Gaussian linear models for repeated measures (or the mixed model repeated measures [MMRM]) with intervention group, center, time (week 1, 2, 4, 8, and 12), and intervention by time interaction as factors, and baseline MADRS score (week 0) as a covariate.

Secondary dichotomous efficacy end points, including response and remission rates based on MADRS within 12 weeks, were analyzed using logistic regression models, with intervention and center as factors and baseline MADRS (week 0) as a covariate. Treatment effects are expressed as odds ratios with 95% CI and *P* values for testing the null hypothesis of no treatment effect are reported. Analyses of continuous secondary end points, including SOFAS and EQ-5D-3L, followed the same approach as the primary outcome.

Adverse events and serious adverse events (SAEs) were summarized as frequencies and percentages by treatment group. For recurrent events and variable follow-up times, event rates were compared between simvastatin and placebo groups using rate ratios with 95% CIs, calculated via Poisson regression with offset for follow-up time.

All hypothesis tests were performed 2-sided at a significance level of 5%. Secondary, exploratory, and safety outcomes were not adjusted for multiple testing due to their exploratory nature. Statistical analyses were performed from July to October 2024 using R, version 4.3.3 (R Project for Statistical Computing).

## Results

### Enrollment and Baseline Patient Characteristics

Between August 21, 2020, and June 06, 2024, we enrolled 161 patients from 9 sites in Germany, with 160 (placebo: n = 79, simvastatin: n = 81; mean [SD] age, 39.0 [11.0] years; 126 female [79%]; 34 male [21%]) analyzed in the ITT population (eFigure 1 in Supplement 2). Enrollment by site and group is provided in eTable 1 in Supplement 2.

**Figure 1.**
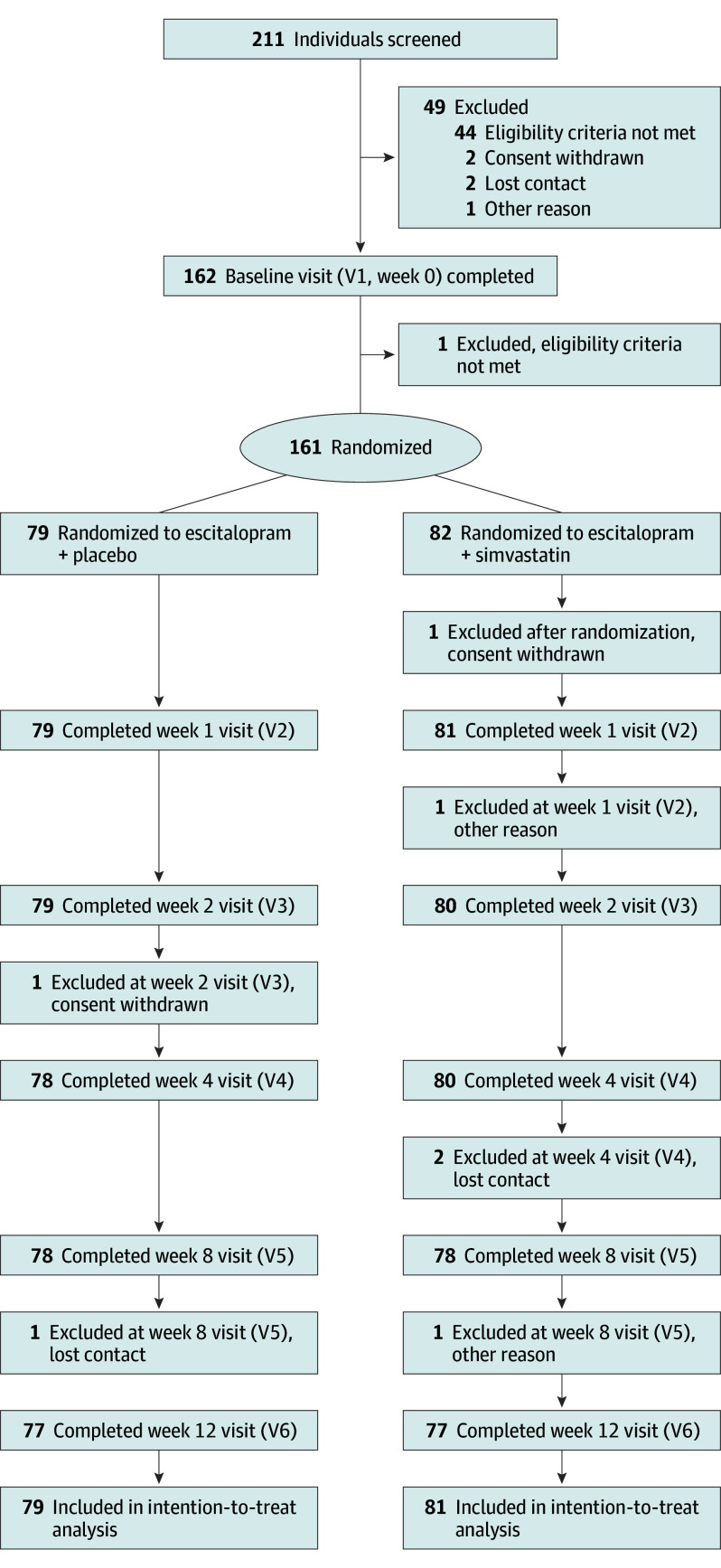
Consolidated Standards of Reporting Trials (CONSORT) Flowchart V indicates visit.

Retention in the trial was excellent (95.6% with 7 participants discontinuing the study prematurely before week 12, n = 3 in the simvastatin group and n = 4 in the placebo group). Participant flow of the trial is illustrated in Figure 1. Data on pill count, escitalopram dosage at end of study, and concurrent medication can be found in eTables 2 to 4 in Supplement 2. In total, 93 participants received any concurrent medication.

Participants on average had moderate depression at baseline. As expected, cardiometabolic risk profiles were very common in the trial population. Absolute numbers and detailed demographic and clinical characteristics by treatment group are provided in Table 1.

**Table 1.** Clinical and Demographic Baseline Characteristics According to the Intention-to-Treat Sample

Characteristic	Overall (N = 160)	Escitalopram + placebo (n = 79)	Escitalopram + simvastatin (n = 81)
**Sex, No. (%)**			
Female	126 (79)	63 (80)	63 (78)
Male	34 (21)	16 (20)	18 (22)
**Age, y**			
Mean (SD)	39 (11)	39 (12)	39 (11)
Median (IQR)	38 (30-48)	38 (31-49)	38 (29-46)
Range	19-64	19-63	20-64
**Body mass index** ^a^			
Mean (SD)	39.1 (6.3)	38.5 (5.1)	39.6 (7.3)
Median (IQR)	38.0 (34.2-42.4)	37.6 (34.7-41.4)	38.7 (33.5-43.1)
Range	29.1-62.2	29.1-52.0	30.2-62.2
**MADRS**			
Mean (SD)	25.5 (5.0)	25.2 (5.2)	25.8 (4.8)
Median (IQR)	25.0 (22.0-29.0)	24.0 (21.5-28.0)	26.0 (22.0-29.0)
Range	13.0-42.0	17.0-42.0	13.0-35.0
**Duration of current episode, wk**			
Median (IQR)	32 (20-76)	40 (20-82)	27 (16-53)
**No. of previous episodes**			
Median (IQR)	3 (2-5)	3 (2-4)	3 (2-7)
**Chronic depression, No. (%)** ^b^			
No	120 (82)	56 (79)	64 (84)
Yes	27 (18)	15 (21)	12 (16)
**Smoking status, No. (%)**			
No	123 (77)	59 (75)	64 (79)
Yes	37 (23)	20 (25)	17 (21)
**Blood pressure (systolic), mm Hg**			
Mean (SD)	135 (14)	135 (12)	135 (16)
Median (IQR)	134 (125-145)	134 (126-146)	135 (123-144)
Range	99-192	110-166	99-192
**Blood pressure (diastolic), mm Hg**			
Mean (SD)	88 (11)	87 (10)	88 (12)
Median (IQR)	87 (80-94)	87 (81-94)	86 (80-94)
Range	47-120	47-108	65-120
**Hypertension, No. (%)**			
No	43 (27)	19 (24)	24 (30)
Yes	117 (73)	60 (76)	57 (70)
**LDL >100mg/dL, No. (%)**			
No	35 (22)	18 (23)	17 (21)
Yes	122 (78)	60 (76)	62 (79)
**Total cholesterol >200mg/dL, No. (%)**			
No	89 (57)	46 (59)	44 (54)
Yes	68.0 (43)	32.0 (41)	36.0 (46)
**Metabolic syndrome**, **No. (%)**^c^			
No	101 (65)	50 (65)	51 (65)
Yes	55 (35)	27 (35)	28 (35)
**CRP ** **>3 mg/L, No. (%)**			
No	55 (35)	25 (32)	30 (38)
Yes	102 (65)	53 (68)	49 (62)

Abbreviations: CRP, C-reactive protein; LDL, low-density lipoprotein cholesterol; MADRS, Montgomery-Åsberg Depression Rating Scale.

SI conversion factor: To convert LDL and total cholesterol to millimoles per liter, multiply by 0.0259; CRP to milligrams per deciliter, divide by 10.

^a^ Body mass index is calculated as weight in kilograms divided by height in meters squared.

^b^ Chronic depression was defined as duration of current depressive episode for 104 weeks or longer.

^c^ Metabolic syndrome was defined as having abdominal girth of 80 cm or greater (women) or 94 cm or greater (men) and fulfilling at least 2 of the following criteria: (1) decreased high-density lipoprotein cholesterol, ie, less than 50 mg/dL (1.29 mmol/L) in women and less than 40 mg/dL (1.03 mmol/L) in men, (2) increased blood pressure, ie, greater than or equal to 130 mm Hg (systolic) or greater than or equal to 85 mm Hg (diastolic), and/or (3) hemoglobin A_1c_ level greater than or equal to 6.5% (48 mmol/mol).

### Blinding Assessment

Blinding was effective as indicated by about 50% of participants correctly guessing their assigned group, and there was no difference between simvastatin and placebo (weighted Cohen κ, −0.001; 95% CI, −0.021 to 0.019).

### Primary Outcome and Sensitivity Analyses

**Figure 2.**
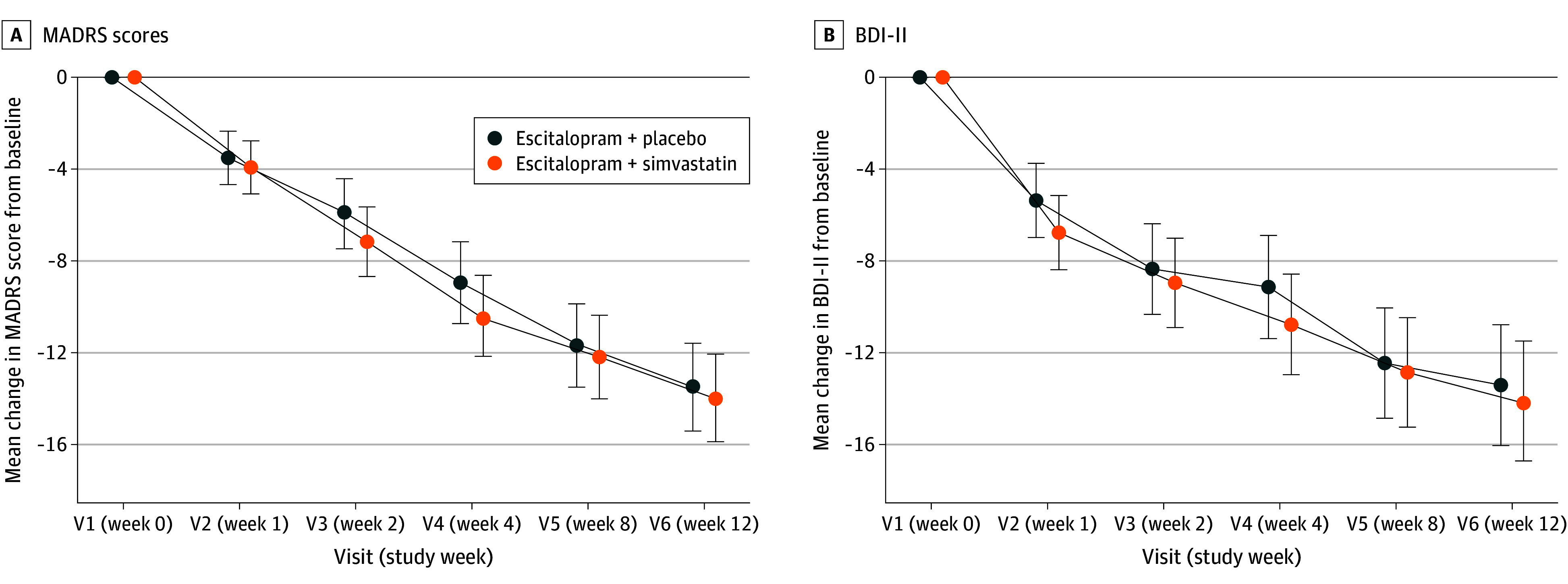
Mean Change in Montgomery-Åsberg Depression Rating Scale (MADRS) Scores Over 12 Weeks and Mean Change in Beck Depression Inventory II (BDI-II) Scores A, MADRS scores. B, BDI-II scores. Error bars represent 95% CIs.

The ITT population comprised 160 patients with at least 1 postbaseline measure of the MADRS. MADRS scores from baseline to week 12 decreased substantially in both groups (simvastatin, −13.97 points; 95% CI, −15.88 to −12.06; placebo, −13.50 points; 95% CI, −15.41 to −11.58) (Figure 2A). Primary end point analysis in the ITT sample showed no significant treatment effect of add-on simvastatin compared with add-on placebo in MADRS scores (MMRM least squares mean difference, 0.47 points; 95% CI, −2.08 to 3.02; *P* = .71). Preplanned sensitivity analyses robustly confirmed this result (Table 2).

**Table 2.** Effects of Treatment on Primary, Secondary, and Exploratory End Points

End point	Group difference	*P* value	Cohen *d* (95% CI)
Primary end point, least squares mean (95% CI)			
MADRS (ITT, MMRM)	0.47 (−2.08 to 3.02)	.72	0.06 (−0.26 to 0.38)
MADRS (ITT, MI)^a^	0.22 (−2.37 to 2.70)	.86	0.03 (−0.30 to 0.34)
MADRS (ITT, LOCF)^a^	0.41 (−2.13 to 2.95)	.75	0.05 (−0.27 to 0.37)
Key secondary end point, least squares mean (95% CI)			
BDI-II	0.69 (−2.80 to 4.18)	.70	0.07 (−0.29 to 0.43)
Secondary end points, OR (95% CI)			
MADRS response	1.39 (0.70 to 2.76)	.35	NA
MADRS remission	1.13 (0.57 to 2.24)	.73	NA
BDI-II MCID	0.83 (0.37 to 1.88)	.66	NA
Exploratory end points, least squares mean (95% CI)			
CGI-S	0.03 (−0.34 to 0.41)	.86	0.01 (−0.07 to 0.09)
SOFAS	−0.42 (−4.53 to 3.69)	.84	−0.03 (−0.34 to 0.28)
EQ-5D (VAS)	−2.41 (−8.26 to 3.44)	.42	−0.13 (−0.44 to 0.18)
CGI-I response, OR (95% CI)	1.01 (0.51 to 2.00)	.98	NA
PGIC response, OR (95% CI)	0.89 (0.45 to 1.74)	.72	NA
HDL, estimated group difference (95% CI), mg/dL	0.55 (−1.80 to 2.90)	.65	0.08 (−0.25 to 0.40)
LDL, estimated group difference (95% CI), mg/dL	36.60 (28.73 to 44.46)	<.001	1.14 (0.90 to 1.39)
Total cholesterol, estimated group difference (95% CI), mg/dL	34.19 (22.95 to 45.42)	<.001	0.86 (0.58 to 1.14)
CRP, estimated group difference (95% CI), mg/L	1.62 (0.55 to 2.68)	.003	0.44 (0.15 to 0.73)

Abbreviations: BDI-II, Beck Depression Inventory II; CGI-I, Clinical Global Impression Scale for Improvement; CRP, C-reactive protein; EQ-5D, EuroQol-5 Dimensions; HDL, high-density lipoprotein cholesterol; ITT, intention to treat; LDL, low-density lipoprotein cholesterol; LOCF, last observation carried forward; MADRS, Montgomery-Åsberg Depression Rating Scale; MCID, minimal clinically important difference; MI, multiple imputation; MMRM, mixed models repeated measures; NA, not applicable; OR, odds ratio; PGIC, Patient Global Impression of Change; SOFAS, Social and Occupational Functioning Assessment Scale; VAS, visual analog scale.

SI conversion factor: To convert HDL, LDL, and total cholesterol to millimoles per liter, multiply by 0.0259; CRP to milligrams per deciliter, divide by 10.

^a^ Prespecified sensitivity analyses for primary end point.

### Secondary Outcomes and Exploratory Outcomes

Similarly, the key secondary end point of patient-reported depression severity (BDI-II) showed decreases in depressive symptoms in both groups but no significant treatment effect for simvastatin (Figure 2B and Table 2).

Furthermore, no significant group differences were observed in secondary end points MADRS response, MADRS remission, or BDI-II MCID (eFigure 2 in Supplement 2 and Table 2).

Confirming the lack of simvastatin effects on mental health outcomes, no significant group differences were observed in any of the exploratory end points for quality of life (EQ-5D), social functioning (SOFAS), or global impression of change from the participants’ or clinicians’ point of view (CGI and PGI) (Table 2).

**Figure 3.**
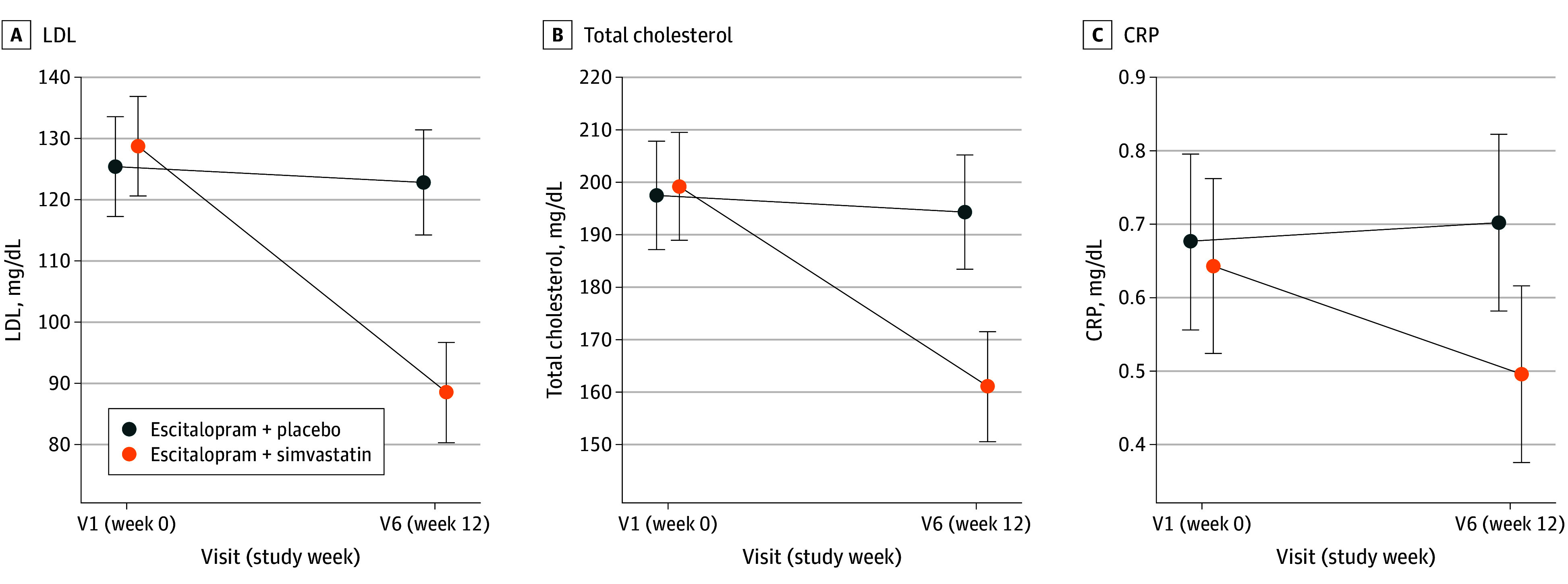
Treatment Effects on Low-Density Lipoprotein (LDL) Cholesterol, Total Cholesterol, and C-Reactive Protein (CRP) Stratified by Visit and Treatment Group A, LDL cholesterol. B, Total cholesterol. C, CRP. Error bars represent 95% CIs. LDL and total cholesterol were only assessed at baseline and after 12 weeks in order to not compromise blinding. SI conversion factor: To convert LDL and total cholesterol to millimoles per liter, multiply by 0.0259; CRP to milligrams per liter, multiply by 10.

In contrast to the mental health end points, simvastatin treatment had significant effects on metabolic health as evidenced by substantial reductions compared with placebo in LDL (simvastatin, −40.37 mg/dL; 95% CI, −47.41 to −33.33 mg/dL; placebo, −3.78 mg/dL; 95% CI, −11.18 to 3.62 mg/dL; *P* < .001; to convert LDL to millimoles per liter, multiply by 0.0259), total cholesterol (simvastatin, −39.07 mg/dL; 95% CI, −49.42 to −28.73 mg/dL; placebo, −4.89 mg/dL; 95% CI, −15.64 to 5.87 mg/dL; *P* < .001; to convert total cholesterol to millimoles per liter, multiply by 0.0259), and CRP (simvastatin, −1.04 mg/L; 95% CI, −1.89 to −0.20 mg/L; placebo, 0.57 mg/L; 95% CI, −0.28 to 1.42 mg/L; *P* = .003; to convert CRP to milligrams per deciliter, divide by 10) levels compared with placebo (eFigure 3 in Supplement 2 and Figure 3). These results suggest excellent adherence to the allocated treatment groups in our trial.

Descriptive statistics for baseline and posttreatment values for all primary, secondary, and exploratory end points by group can be found in eTable 5 in Supplement 2. The baseline and posttreatment values for body weight and BMI can be found in eTable 7 in Supplement 2.

Prespecified subgroup analyses on baseline characteristics that could influence treatment response to simvastatin, including sex, severity of obesity, metabolic syndrome, inflammatory status, and depression severity or chronicity, yielded no significant results (eFigure 4 in Supplement 2).

### Safety Outcomes

Four SAEs occurred in 2 patients, with no significant differences between groups. Three of the SAEs were associated with 1 participant who experienced an unintended pregnancy, followed by spontaneous abortion a few days later, necessitating hospitalization for curettage due to multiple uterine myomas. The remaining SAE involved hospitalization in 1 patient due to worsening of depressive symptoms. A total of 123 participants reported AEs during the trial, mainly headaches and nausea, with no differences between simvastatin (61 participants with AEs) and placebo (62 participants with AEs). eFigure 5 in Supplement 2 shows the distribution of suicidal symptoms (assessed by MADRS item 10) at screening, baseline, and subsequent study visits. None of the participants exhibited high levels of suicidality (MADRS item 10 >4) at any time point, and the percentage of patients with low levels of suicidality increased over the course of the study in both groups. All SAEs and AEs are listed in eTable 6 in Supplement 2.

### Updated Meta-Analysis

To integrate the data from our trial with the previous evidence from the literature, we conducted an updated systematic review and meta-analysis of all available evidence. Included trials were of variable quality, with the smallest 5 trials receiving high or moderate risk of bias ratings. The 3 largest RCTs had a low risk of bias (eFigure 6A in Supplement 2). Overall, the updated meta-analysis revealed a small, albeit statistically significant, benefit of statins, but this was strongly driven by the low-quality trials (eFigure 6B in Supplement 2). Accordingly, stratified analyses demonstrated no significant benefit of statins in those RCTs with a low risk of bias (eFigure 6B in Supplement 2).

## Discussion

In this RCT of patients with comorbid MDD and obesity, we did not find antidepressive effects of simvastatin, 40 mg per day, as add-on treatment to escitalopram compared with add-on placebo. This lack of effect was robust across primary and secondary outcomes and confirmed in subgroup analyses. However, as expected, we found significant simvastatin effects on total cholesterol and LDL and CRP values. Simvastatin was not associated with increased AEs or SAEs compared with placebo.

Our results suggest that simvastatin does not exert additional antidepressive effects when added to escitalopram in patients with comorbid MDD and obesity, despite significantly lowering lipids and CRP level. MDD has been associated with numerous alterations of the immune system,^29,30^ and obesity is consistently associated with subclinical inflammation and increased cardiovascular disease risk.^28^ In our sample, 73% of participants (117 of 160) had hypertension, 35% of participants (55 of 160) met modified criteria of metabolic syndrome, and 65% (102 of 160) had CRP levels greater than 3 mg/L, an established cutoff for increased cardiovascular risk.^31^ However, despite these metabolic and immune alterations, we failed to demonstrate an antidepressive effect of simvastatin.

Given the current evidence, we believe it is unlikely that statins have clinically meaningful acute antidepressive effects when added to standard antidepressants. It is noteworthy that our meta-analysis demonstrated a clear contrast between smaller studies (<70 participants) with high/moderate risk of bias vs trials with larger samples (70-160 participants) and low risk of bias. Although the former studies have predominantly shown significant effects, the latter studies found an almost 3-fold reduced effect size and have consistently failed to demonstrate additional antidepressive effects of statins in different populations with MDD. This is in line with general meta-analytic evidence showing that smaller trials typically report larger effect sizes compared with those observed in larger trials^32^ and a positive association between risk of bias and effect size.^33,34^

Even though simvastatin did not exert additional antidepressive effects in our study, it had the expected and well-known effects on lipids and inflammatory activity. Given that both MDD and obesity are associated with increased cardiovascular risk and higher mortality,^35^ statins should be prescribed in this comorbid group of patients following the guidelines for statin use in primary prevention.^36^ Clinicians should not be discouraged by the lack of additional antidepressive statins effects in our study but should continue prescribing lipid lowering medications to reduce cardiovascular risk in these patients. This is important, because patients with mental disorders are less likely to receive medication for cardiovascular indications than individuals without mental disorders, despite their increased cardiovascular risk.^37^ It is reassuring that, in our study, simvastatin was equally well tolerated and safe as placebo.

### Strengths and Limitations

Our study had several strengths. First, to date and to our knowledge, our study was the largest study among those that examined putative antidepressive statin effects. Second, there was an excellent retention rate and good adherence to the medication. Third, blinding was effectively maintained. Finally, we involved experts by experience from the very beginning, ie, already in the planning process of the study and before grant application.

However, our study also has some limitations. It was conducted in tertiary care centers in a Western, high-income country, in patients with moderate symptom severity and an overall comparatively high response rate, limiting its generalizability. Further, we did not include participants with an established indication for statin treatment. We, therefore, cannot rule out that statins may exert antidepressive effects in populations with an indication for statin treatment, eg, after myocardial infarction or stroke. However, given the high cardiovascular risk in our sample, we believe it is unlikely that putative antidepressive statin effects would clinically meaningfully differ in populations with a statin indication. Finally, as required by our ethics review board, we excluded patients with a lifetime history of suicide attempt.

## Conclusions

In summary, results of this RCT reveal that simvastatin did not exert additional antidepressive effects when added to escitalopram in patients with comorbid MDD and obesity, despite improving the cardiovascular risk profile. Statins should be used in these patients according to guidelines for primary and secondary prevention of cardiovascular diseases.

## Notes

Supplement 1.
Trial Protocol and Statistical Analysis Plan.

Supplement 2.
eMethods.
**eTable 1.** Recruitment by Site and Group Allocation **eTable 2.** Pill Count Overall and by Treatment Group **eTable 3.** Escitalopram Dosage at End of Study (week 12) Overall and by Treatment Group **eTable 4.** Concurrent Medication Given as Proportions of the Population Taking at Least 1 Medication From the Corresponding Categories **eTable 5.** Baseline and Posttreatment (Week 12) Values for Primary, Secondary, and Exploratory Outcomes **eTable 6.** (Serious) Adverse Events Overall and by Treatment Group **eTable 7.** Change in Body Weight and Body Mass Index Overall and by Treatment Group **eFigure 1.** PRISMA Flow Diagram **eFigure 2.** Probability for MADRS Response and Remission After 12 Weeks With Corresponding 95% CIs **eFigure 3.** Prevalence of LDL, Total Cholesterol, and CRP Over a Predefined Cutoff With Corresponding 95% CIs, Stratified by Visit and Treatment Group **eFigure 4.** Subgroup Analyses **eFigure 5.** Suicidal Symptoms, Assessed by MADRS Item 10, at Screening, Baseline, and Throughout the Study by Treatment Group **eFigure 6.** Meta-Analysis of All Available Evidence From RCTs Evaluating the Efficacy of Statins for Depression Severity in Adults With MDD
eReferences.

Supplement 3.
Data Sharing Statement.
