# Supplementary material for: Simvastatin as Add-On Treatment to Escitalopram in Patients With Major Depression and Obesity: A Randomized Clinical Trial
Source: JAMA Psychiatry. 2025 Jun 4;82(8):759–67. doi: 10.1001/jamapsychiatry.2025.0801 (PMC12138799; doi:10.1001/jamapsychiatry.2025.0801)
Supplement: Supplement 1. — Trial Protocol and Statistical Analysis Plan. [file jamapsychiatry-e250801-s001.pdf]

CHARITÉ
UNIVERSITÄTSMEDIZIN BERLIN

NeuroCure
Clinical Research Center (NCRC)

Simvastatin add-on to Escitalopram in patients with comorbid obesity and major depression:

A multicenter, randomized, double-blind, placebo-controlled trial

EudraCT-Number
2018-002947-27

Protocol code
SIMCODE

Protocol

Version1.4 / 24.09.2021

Sponsor of the clinical study

Charité – Universitätsmedizin Berlin, Chariteplatz 1, 10117 Berlin

Principal Investigator

Prof. Dr. Christian Otte, Charité - Universitätsmedizin Berlin
Klinik für Psychiatrie und Psychotherapie, Campus Benjamin Franklin
Hindenburgdamm 30, 12203 Berlin

Biostatistician:

Prof. Dr. Tim Friede, Universitätsklinikum Göttingen

The following persons accept the content of this protocol and confirm to conduct this study in compliance with Good Clinical Practice and applicable regulatory requirements.

Representative of Sponsor and Principal Investigator	Prof. Dr. med. Christian Otte	26.01.22<br>Berlin, date

- Confidential -

The information contained in this protocol has to be kept strictly confidential. Therefore the protocol is only provided to investigators in confidence for review, to study staff, Independent Ethics Committee/Institutional Review Board, monitor, regulatory authorities and CROs (or KKS) and for obtaining written informed consent from patients.

Table of contents

1. Synopsis 1
2. Synopse (Deutsch) 12
3. Flow-Chart 24
4. Introduction 24
4.1 Introduction and background 24
4.2 Data on IMP-Application 25
4.2.1 Summary 25
4.2.2 Physical, Chemical, and Pharmaceutical Properties and Formulation 26
4.2.3 Nonclinical Studies 26
4.2.4 Effects in humans 27
4.2.5 Summary of Data and Guidance for the Investigator 29
4.3 Rationale 29
5. Study objectives 30
5.1 Primary objective 30
5.1.1 Primary efficacy (confirmatory) endpoint 31
5.2 Secondary objectives 31
5.2.1 Secondary efficacy (exploratory) endpoints 31
5.2.2 Other secondary (exploratory) endpoints 31
6. Study population 32
6.1 Number of patients 32
6.2 Selection of patients 33
6.2.1 Inclusion criteria 33
6.2.2 Exclusion criteria 34
6.2.3 Contraception 35
6.2.4 Follow up of pregnancy 35
6.2.5 Methods of avoiding simultaneous enrolment in other trials 35
6.2.6 Screening failures 36
7. Plan for medical treatment 36
7.1 Study design 36
7.2 Description of study medication 36
7.2.1 Investigational Medical product (IMP) 36
7.2.2 Standard medication 37
7.2.3 Control (Placebo) 37
7.2.4 Treatment schedule / Dose regimen 37
7.2.5 Adverse drug reactions and restrictions, contraindications, drug interactions 38
7.2.6 Procedures in case of emergency 40
7.2.7 Randomization 40
7.2.8 Packaging, labelling, and blinding procedures 40
7.2.9 Unblinding 40
7.2.10 Storage requirements and administration (shipping and destruction) 41
7.2.11 Treatment compliance 41
8. Concomitant medication / concomitant therapy 41
9. Study procedures 42
9.1 Recruitment / Screening procedures 42
9.2 Informed consent 42
9.3 Patient identification / Generation of pseudonym 42
9.4 Visits schedule and evaluations 43
9.4.1 Screening visit 44
9.4.2 Randomization visit (Baseline) - Drug dispensation 44
9.4.3 Examinations during trial 45
9.4.4 Final examination (End-of-study) 46
9.5 Evaluations / Efficacy assessments 47

9.5.1 MADRS ... 47
9.5.2 BDI-II ... 47
9.6 Evaluations / Safety assessments ... 47
9.6.1 Clinical examination ... 47
9.6.2 Laboratory safety ... 47
9.6.3 Electrocardiogram ... 48
9.6.4 MINI International Neuropsychiatric Interview ... 48
9.7 Evaluations / Other assessments ... 48
9.7.1 Patient reported outcome measures ... 48
9.7.2 Laboratory assessments ... 49
9.7.3 Other procedures ... 49
10. Risk-benefit-assessment ... 49
11. Termination and subsequent treatment ... 51
11.1 Premature termination of the individual participant ... 51
11.2 Premature termination of the clinical study ... 51
11.3 Follow-up and continuing treatment after regular / premature termination ... 52
12. Adverse events ... 52
12.1 Definitions ... 52
12.1.1 Adverse Event (AE) ... 52
12.1.2 Adverse Reactions (AR) ... 52
12.1.3 Serious Adverse Event (SAE) / Serious Adverse Reaction (SAR) ... 52
12.1.4 Exceptional rules ... 52
12.1.5 Suspected Unexpected Serious Adverse Reactions (SUSAR) ... 53
12.2 Treatment of (S)AEs ... 53
12.3 Assessment of AEs and SAEs ... 53
12.3.1 Assessment of intensity ... 53
12.3.2 Assessment of causality ... 53
12.4 Documentation of AEs and SAEs ... 54
12.5 Reporting of SAEs and SUSARs ... 54
12.5.1 Reporting of SAEs / Contact for SAE reporting ... 54
12.5.2 Reporting of Suspected Unexpected Serious Adverse Reactions (SUSARs) ... 55
12.5.3 Other safety issues requiring expedited reporting ... 55
12.6 Follow-up of adverse events ... 55
12.7 Data Monitoring Committee ... 56
13. Documentation ... 56
13.1 Source Data and Study Files ... 56
13.2 Case Report Forms (CRF) ... 56
13.3 Investigator Site File (ISF) ... 56
13.4 Drug accountability / Flow of study medication ... 56
14. Quality management ... 56
14.1 Control of trial progress and data quality ... 56
14.1.1 Monitoring, Data Quality Assurance ... 57
14.1.2 Audits / Inspections ... 57
14.2 Standardisation and validation ... 57
14.3 Reference institutions ... 57
15. Data entry und data management ... 58
15.1 Source Data and Study Files ... 58
15.2 Data collection / Case Report Form (CRF) ... 58
15.3 Data management ... 58
16. Statistical Analysis ... 59
16.1 Hypotheses ... 59
16.1.1 Primary hypothesis ... 59
16.1.2 Secondary hypotheses ... 59
16.2 Sample size estimation ... 59

16.3 Definition of population for analysis 60
16.4 Analysis of primary and secondary endpoints 60
16.5 Analysis of adverse events 61
16.6 Interim analyses and endpoints for premature study termination 61
17. Reporting 61
17.1 Statistical report 61
17.2 Final report 61
17.3 Publication (policy) 61
18. Ethical, legal and regulatory aspects 62
18.1 ICH-GCP-guidelines 62
18.2 Legal requirements of the study 62
18.2.1 Approval of Ethics Committee 62
18.2.2 Approval of competent authority 62
18.2.3 Notification to regional authorities 62
18.2.4 Patient information and informed consent 62
18.2.5 Patient insurance 62
18.2.6 Data Privacy and confidentiality 63
18.3 Archiving of data / Access to records 63
18.4 Financing 63
19. References 64
20. Appendices 69
20.1 Stellungnahme zur Geschlechterverteilung 69
20.2 Stellungnahme zur Weiterbehandlung nach Studienende 69
20.3 Zusammenfassende Nutzen-Risiko-Abwägung 69
20.4 Ergänzende Informationen zum Prüfpräparat 69

Abbreviations

ADL	Activities of daily living
AE	Adverse Event
ALT/GPT	Alanine aminotransferase
AMG	Arzneimittelgesetz
AR	Adverse Reactions
AST/GOT	Aspartate transaminase
BCRP	Breast Cancer Resistance Protein
BDI-II	Beck Depression Inventory II
BDNF	Brain Derived Neurotrophic Factor
Beta-HCG	β-Subunit of hCG gonadotropin
BfArM	Bundesinstitut für Arzneimittel und Medizinprodukte
BMBF	Bundesministerium für Forschung und Bildung
BMI	Body-Mass-Index
CD-ROM	Compact Disc Read-Only Memory
CDT	Carbohydrate Deficient Transferrin
CI	Confidence interval
CGI-I	Clinical Global Impression scale – Improvement
CGI-S	Clinical Global Impression scale – Severity of illness
CHD	Coronary heart disease
CK	Creatine kinase
CNS	Central nervous system
CONSORT	Consolidated Standards of Reporting Trials
CRF	Case Report Form
CRP	C-reactive protein
CTCAE	Common Terminology Criteria for Adverse Events
CYP3A4	Cytochrome P450 3A4
DMC	Data Monitoring Committee
DSM 5	Diagnostic and Statistical Manual of Mental Disorders 5th Edition)
DSMB	Data and Safety Monitoring Board
ECG/EKG	Electrocardiography/Elektrokardiogramm
eCRF	Electronic case report form
ECT	Electroconvulsive therapy
EDC	Electronic data capture system
EMA	European Medicines Agency
EQ-5D-3L	EuroQol-5 Dimensions-3 Levels Questionnaire
ESC	Escitalopram
EWR	Europäische Wirtschaftsraum
FACS	fluorescence activated cell sorting
FDA	Food and Drug Administration
Gamma-GT	Gamma-glutamyltransferase
GBD	Global Burden of Disease
GCP	Good Clinical Practice
GFR	Glomerular filtration rate
HbA1c	Glycated hemoglobin
HDI	Haftpflichtverband der Deutschen Industrie
HDL	High-density lipoprotein
HIV	Human immunodeficiency virus
iAS	interActive Systems

ICD	International Statistical Classification of Diseases and Related Health Problems
ICH	International Conference on Harmonisation of technical requirements for registration of pharmaceuticals for human use
ID	Identity document
IEC	Independent Ethics Committee
IL-1beta	Interleukin 1 beta
IL-6	Interleukin 6
IMP	Investigational Medical Product
IRB	Institutional review board
ISF	Investigator site file
ITT	Intent to treat Population
KKS	Koordinierungszentrum für klinische Studien
LDL	Low-density lipoprotein
LOCF	last-observation-carried-forward
MADRS	Montgomery-Asberg-Depression Rating Scale
MAO inhibitor	Monoamine oxidase inhibitors
MCH	Mean corpuscular hemoglobin
MCHC	Mean corpuscular hemoglobin concentration
MCID	Minimal clinically important difference
MCV	Mean corpuscular volume
MDD	Major depressive disorder
MDE	Major depressive episode
mg/d	Milligram/day
MINI	Mini International Neuropsychiatric Interview
MMRM	Mixed Model for Repeated Measures
MPV	Mean platelet volume
NCRC	NeuroCure Clinical Research Center
NMDA	N-Methyl-D-aspartic acid
PBMC	peripheral blood mononuclear cells
PGIC	Patients’ Global Impression of Change Scale
PI	Principal Investigator*
PLC	Placebo Statistical analysis
PPP	Per protocol population
qRT-PCR	quantitative real-time polymerase chain reaction
QTc	corrected QT interval
RCT	Randomized controlled trial
RDE	Remote data entry
RDW-CV	Red blood cell distribution width
SAE	Serious Adverse Event
SAR	Serious Adverse Reaction
SAS	Statistical Analysis System
SDV	Source data verification
SIM	Simvastatin
SIMCODE	Simvastatin add-on to Escitalopram in patients with comorbid obesity and major depression: A multicenter, randomized, double-blind, placebo-controlled trial
SOFAS	Social and Occupational Functioning Assessment Scale
SSL	Secure data transfer-protocol
SSRI	Selective Serotonin Reuptake Inhibitor

* will be used as a synonym for Representative of Sponsor.

SUSAR	Suspected Unexpected Serious Adverse Reaction
TG	triglyceride
TMF	trial master file
TNF	tumor necrosis factor
YLD	Years lived with disability
ULN	upper limit normal
VLDL	Very-low-density lipoprotein

1. Synopsis

**Study title**	Simvastatin add-on to Escitalopram in patients with comorbid obesity and major depression: A multicenter, randomized, double-blind, placebo-controlled trial (SIMCODE)
**Type of project**	Randomized, placebo-controlled, double-blind multicenter trial with parallel-group design, Phase II
**Sponsor**	Charité – Universitätsmedizin Berlin, Charitéplatz 1, 10117 Berlin
**(Principal) investigator (PI)**	Prof. Dr. Christian Otte<br>Charité - Universitätsmedizin Berlin, Klinik für Psychiatrie und Psychotherapie, Campus Benjamin Franklin, Hindenburgdamm 30, 12203 Berlin<br>Tel: 030-450517531; Fax: 030-450 517942<br>Email: christian.otte@charite.de
**Administrative support by**	NeuroCure Clinical Research Center (NCRC), Charité - Universitätsmedizin Berlin, Campus Mitte, Charitéplatz 1, 10117 Berlin<br>Koordinierungszentrum für Klinische Studien (KKS Charité), Charité - Universitätsmedizin Berlin, Campus Virchow-Klinikum, Augustenburger Platz 1, 13353 Berlin
**Study Centers**	Coordinating investigator: Prof. Dr. Christian Otte, Charité - Universitätsmedizin Berlin, Klinik für Psychiatrie und Psychotherapie, Campus Benjamin Franklin<br>Investigational centers: 9 centers located in Germany.<br>Data and Safety Monitoring Board (DSMB):<br>- Prof. Dr. Brenda Penninx, Vrije Universiteit Amsterdam<br>- Prof. Dr. Falk Kiefer, Zentralinstitut für Seelische Gesundheit, Mannheim<br>- Prof. Dr. Carsten Spitzer, Fachklinik Universitätsmedizin Rostock<br>- Mrs. Francoise Margue, Deutsche Depressionsliga
**Timetable**	Recruitment start / start of study: (first patient first visit) Q1 2020<br>End of recruitment Q1 2023<br>End of study[1] (last patient last visit) Q2 2023<br>Trial duration (for a patient): 12 weeks<br>follow up: none
**Rationale**	Major depressive disorder (MDD) and obesity are major contributors to impaired health worldwide. Statins are among the most prescribed medications with well-established safety and efficacy. Statins are recommended in primary prevention of cardiovascular disease, which has been linked to both MDD and obesity. Moreover, statins are promising candidates to treat MDD because a meta-analysis of pilot randomized controlled trials has found antidepressive effects of statins as adjunct therapy to antidepressants. However, no study so far has tested the antidepressive potential of statins in patients with MDD and comorbid obesity

[1] 2010/C 82/01, 2.5, 43.

**Study objectives**	**<u>Primary objective</u>**<br>To examine whether add-on 40 mg/d Simvastatin to standard antidepressant medication (Escitalopram 20 mg/d) improves depression to a greater extent than adjunct placebo in patients with major depression and comorbid obesity<br>**<u>Secondary objectives</u>**<br>To examine whether add-on 40 mg/d Simvastatin to standard antidepressant medication (Escitalopram 20 mg/d) improves response rates, remission rates, patients' impression of change, clinicians impression of severity and change, quality of life, social functioning, self-report depression, lipid values, and immunometabolism / mitochondrial function to a greater extent than adjunct placebo in patients with major depression and comorbid obesity
**Hypotheses / endpoints**	**<u>Primary hypothesis</u>**:<br>Simvastatin (40 mg/d) add-on to standard antidepressant Escitalopram (20 mg/d) will improve depression to a greater extent than add-on placebo in patients with comorbid obesity and major depression<br>**<u>Primary efficacy (confirmatory) endpoint</u>**<br>Change score from baseline to week 12 in Montgomery-Asberg-Depression Rating Scale (MADRS)<br>**<u>Secondary Hypotheses</u>**:<br>Add-on Simvastatin (40 mg/d) to standard antidepressant Escitalopram (20 mg/d) will improve<br>**1)** response rates and remission rates<br>**2)** patients' impression of change, clinicians' impression of severity and improvement, quality of life, social functioning, self-report depression<br>**3)** lipid values<br>**4)** mitochondrial and cellular function of immune cells<br>to a greater extent over 12 weeks than adjunct placebo in patients with major depression and comorbid obesity.<br>**<u>Secondary efficacy (exploratory) endpoints</u>**<br>**1)** MADRS-response (50 % MADRS score reduction from baseline), MADRS-remission (MADRS score < 10), and MADRS-minimal clinically important difference (MCID)<br>**2)** change Beck Depression Inventory (BDI-II) scores from baseline to week 12, and BDI-II-MCID<br>**<u>Other secondary (exploratory) endpoints</u>**<br>**2)** change in Patients' Global Impression of Change Scale (PGIC), change in Clinicians' Global Impression of Severity of illness (CGI-S), Clinicians' Global Impression of Improvement (CGI-I), EuroQol-5 Dimensions-3 Levels Questionnaire (EQ-5D-3L), and Social and Occupational Functioning Assessment Scale (SOFAS) from baseline to week 12<br>**3)** change in high-density lipoprotein (HDL), low-density lipoprotein (LDL), and total cholesterol from baseline to week 12

	**4)** change in mitochondrial and cellular function of immune cells from baseline to week 12
**Study design**	multicenter, randomized, double-blind, placebo-controlled, parallel-group
**Study medication / Therapeutical strategy**	**Investigational Medical Product (IMP)**: Simvastatin (fixed dose 40 mg/d over 12 weeks)<br>**Standard-medication**: Escitalopram (fixed dose 10 mg/d - week 1 – 2 and 20 mg/d week 3 - 12)<br>IMP or placebo will be provided as add-on medication to standard antidepressant treatment with Escitalopram
**Control therapy**	Placebo capsules (indistinguishable from IMP) over 12 weeks
**Total number of patients**	160 patients divided into 2 groups:<br>- Group 1 (80 patients): Placebo add-on standard-medication<br>- Group 2 (80 patients): IMP add-on standard-medication
**Study population**	Patients with comorbid obesity (body mass index ≥ 30) and major depression
**Inclusion criteria**	- Written informed consent is present<br>- The patient has the capacity to give consent (He/she is able to understand the nature and anticipated effects/side effects of the proposed medical intervention)<br>- The patient has a major depressive episode according to DSM 5 (Diagnostic and Statistical Manual of Mental Disorders 5th Edition)<br>- The patient has a score of ≥ 18 in the Montgomery-Asberg Depression Rating Scale (MADRS)<br>- The patient has a body mass index ≥ 30<br>- The patient's age is between 18 and 65 years (≥ 18 und ≤ 65)<br>- The patient has not given childbirth within the 6 months prior to study entry and is not breastfeeding<br>- In case of non-psychotropic medication: The patient received stable pharmacological medication for at least 14 days prior to study entry (any changes in medication dose or frequency of therapy must be answered with no)<br>- The patient did not take antidepressants during the last 7 days prior to study entry (discontinuation of effective medication to enable study participation is prohibited)<br>- The patient did not receive prior treatment with Escitalopram in index episode<br>- The patient had less than three (<3) trials with antidepressants in index episode<br>- The patient does not have a history of non-response to Escitalopram<br>- The patient did not receive treatment with ketamine, electroconvulsive therapy (ECT) or other stimulatory treatments in index episode<br>- The patient does not meet any of the following criteria:<br>- schizophrenia<br>- schizoaffective disorder<br>- bipolar disorder

	- The patient is not diagnosed with dementia and does not have moderate or severe impairment of general cognitive function according to clinical impression<br>- The patient does not have clinically relevant elevated liver enzymes [GOT or GPT > 3 x upper limit normal (ULN)] and does not have elevated Carbohydrate Deficient Transferrin (CDT) ≥ 2.4 %<br>- The patient does not meet the criteria for alcohol use disorder (DSM-5: 303.90; ICD-10: F10.20) or substance use disorder (DSM-5: 304; ICD-10: F11.20 – F19.20) in M.I.N.I. for DSM-5 and a urine/serum drug screening is negative (except for benzodiazepines and opiates)<br>- The patient does not have a history of suicide attempt<br>- The patient does not have diagnosed epilepsy or increased bleeding diathesis or a history of angle closure glaucoma or other glaucomas<br>- The patient did not have bariatric surgery prior to study entry<br>- The patient does not have a known allergy or contraindication against Escitalopram or Simvastatin<br>- The patient does not meet any of the following criteria:<br>- hereditary muscle disease<br>- known history of rhabdomyolysis<br>- elevated creatine kinase (CK) outside of the sex-specific reference intervals<br>- History of muscular symptoms under treatment with statins or fibrates<br>- The patient does not have elevated TSH level outside of the age- and sex-specific reference intervals.<br>- The patient does not have insulin-dependent diabetes mellitus<br>- The patient does not have uncontrolled hepatic disorder, renal or cardiovascular disease<br>- The patient does not have untreated hypothyroidism<br>- The patient does not have a history of myocardial infarction or stroke<br>- The patient does not have symptomatic peripheral arterial disease<br>- The patient does not have monogenic familial hypercholesterolemia<br>- The patient does not have clinically significant laboratory abnormalities<br>- The patient did not participate in other interventional trials during the 6 months before and at the time of this trial<br>- The patient is not an employee of the investigator study site, or a family member of the employees or the investigator, or otherwise dependent on the sponsor, the investigator or the investigator study site.
**Exclusion criteria**	- The patient has current use of statins (for visits 2-6 applies: except for IMP Simvastatin)<br>- The patient has current use of antidepressants (for visits 2-6 applies: except for standard medication Escitalopram)<br>- The patient has acute suicidal tendencies (MADRS Item 10 > 4)

- The patient uses potent CYP3A4-inhibitors (e.g. clarithromycin, erythromycin, HIV protease inhibitors – see “Risks, adverse drug reactions, drug interactions, restrictions, contraindications, procedures in case of emergency”)
- The patient uses potent CYP3A4 inductors (carbamazepine, efavirenz, nevirapin, etravirine).
- The patient uses Fibrates, Amiodaron, Amlodipin, Verapamil, Fluconazol, Diltiazem, Fusidic acid, Niacin, Daptomycin or Lomitapid or BCRP-Inhibtors (e.g. Elbasvir or Grazoprevir)
- The patient uses Gemfibrozil, Ciclosporin or Danazol
- The patient has known hypersensitivity to other ingredients of Simvastatin and Escitalopram [butylated hydroxyanisole, microcrystalline celluose, citric acid, starch, lactose, magnesium stearate, hypromellose, talc, titanium dioxide, iron oxies, colloidal silicon dioxide, croscarmellose sodium, polyethylene glycol]
- The patient uses medication that is associated with QTc-prolongation [antiarrhythmica class IA and III, antipsychotics (e.g. haloperidol), phenothiazines, tricyclic antidepressants, antibiotics (e.g. moxifloxacin), and certain antihistaminergic drugs (e.g. astemizol, mizolastin)]
- The patient has clinically significant abnormalities in 12-lead ECG (e.g. QTc-prolongation ≥ 500 ms or increase ≥ 60 ms from baseline visit)
- The patient is pregnant
- The patient with childbearing potential is not willing to use an acceptable form of contraception (defined as Pearl index < 1)
- The patient has current use of psychotropic medication (e.g. antipsychotics, anticonvulsants, lithium or St. John's Wort) except for benzodiazepines, non-benzodiazepines and opiates
- The patient uses nonselective, irreversible monoamine oxidase (MAO) inhibitor (e.g. Tranylcypromine) or selective, reversible inhibitor of monoamine oxidase A (e.g. Moclobemide) or the nonselective, reversible monoamine oxidase inhibitor Linezolid
- The patient is unwilling to consent to saving, processing and propagation of pseudonymized medical data for study reasons
- The patient is legally detained in an official institution
- The patient has active SARS-CoV-2 infection

Visit and documentation schedule

Maximum variation of ± 3 days is permitted for the scheduling of each study visit

Assessments	Screening	Baseline (Visit 1)	Visit 2	Visit 3	Visit 4	Visit 5	End-of-study (Visit 6)
		week 0	week 1	week 2	week 4	week 8	week 12
Screening and consent							
Informed consent	X						
Inclusion criteria		X					
Exclusion criteria		X	X	X	X	X	X
Medical history	X						

Treatment status		X					
M.I.N.I – DSM-5	X						
Urine/serum drug screening	X						
Concomitant medication	X						
Safety							
SARS-CoV-2 Rapid Antigen Test (for all patients without proof of complete vaccination)							
Physical exam	X						X
ECG	X	X	X	X	X	X	X
Blood pressure & heart rate	X	X	X	X	X	X	X
Safety laboratory (incl. Pregnancy test - women only)	X	X			X	X	X
Adverse events recording			X	X	X	X	X
Effectiveness							
MADRS	X	X	X	X	X	X	X
BDI-II		X	X	X	X	X	X
Social functioning and quality of life							
SOFAS		X			X		X
EQ-5D-3L		X			X		X
Other							
Demographics	X						
BMI	X	X					X
Waist circumference		X					X
PGIC			X	X	X	X	X
CGI-S		X	X	X	X	X	X
CGI-I			X	X	X	X	X
Laboratory (lipid assessment)		X					X
Laboratory (immunometabolism / mitochondrial function)		X			X	X	X
Drug dispensation		X		X	X	X	

Note: Electrocardiography (ECG), Body-Mass-Index (BMI), Mini International Neuropsychiatric Interview (MINI), Montgomery-Asberg Depression Scale (MADRS), Beck-Depressions-Inventar (BDI), Patient Global Impression of Change (PGIC), Clinicians' Global Impression of Severity of illness (CGI-S), Clinicians' Global Impression of Improvement (CGI-I), Social and Occupational Functioning Assessment Scale (SOFAS), Generic Quality of Life Questionnaire (EQ-5D-3L)

**Safety**	Assessment of safety: Assessment of blood pressure and heart rate at every visit. Assessment of laboratory parameters – safety laboratory (incl. pregnancy test – women only) at screening, baseline, week 4, week 8 and week 12; physical examination at screening and end-of-study; ECG at every visit.

	Standard procedures for reporting of adverse events will be used. Adverse events will be assessed at every visit starting at visit 2.
**Discontinuation criteria**	**<u>Discontinuation of individual patient</u>**<br>Patients are free to withdraw from participation in the study at any time without specifying any reasons. An investigator may discontinue or withdraw a participant from the study for the following reasons:<br>- If any clinical adverse event (AE), laboratory abnormality, or other medical condition occurs such that continued participation in the study would not be in the best interest of the participant (see also 11.1)<br>**<u>Discontinuation of the trial</u>**<br>According to the German Fachinformation for Simvastatin, the combined incidence for myopathy/rhabdomyolysis in 41.413 patients treated for at least 4 years was 0.08% for 40mg/d Simvastatin. Severe symptomatic hyponatremia (serum sodium [S_{Na}] <125 mmol/L) is a rare but potentially fatal complication of Escitalopram. Signs and symptoms of hyponatremia include headache, difficulty concentrating, memory impairment, confusion, weakness, and unsteadiness and can be as severe as hallucination, syncope, seizure, coma, respiratory arrest, and death. Therefore, the whole study may be discontinued at the occurrence of the treatment-related serious adverse event (SAE) “myopathy/rhabdomyolysis” as defined by the German Fachinformation for Simvastatin [muscle pain, tenderness or weakness with creatine kinase (CK) above ten times the upper limit of normal (ULN)] in more than 5 participants or the treatment-related SAE “severe symptomatic hyponatremia” (serum sodium [S_{Na}] <125 mmol/L) in more than 5 participants. In addition, an early discontinuation of the trial may be decided if new scientific data during the course of the trial changes the risk-benefit-balance significantly. If such data emerges, recruitment and treatment of currently treated patients will be paused immediately. A final decision on continuation or termination of the trial will then made by the Sponsor/PI based on the recommendation of the Data and Safety Monitoring Board (DSMB).
**Statistical analysis**	**<u>Primary efficacy (confirmatory) endpoint</u>:**<br>The primary analysis will compare MADRS change from baseline to week 12 between the Simvastatin and placebo group by Gaussian linear models for repeated measures (so-called MMRM) with intervention, center, time (week 1, 2, 4, 8, and 12), and intervention-by-time interaction as factors, and baseline MADRS score as covariate. The primary analysis population will be the intention-to-treat (ITT) population.<br>**<u>Secondary efficacy (exploratory) endpoints</u>:**<br>The secondary efficacy endpoints include the percentages of MADRS-response, MADRS-remission, and MADRS-MCID within 12 weeks that will be analyzed by logistic regression models with intervention and center as factors and baseline MADRS as covariate. The analyses of secondary efficacy endpoint BDI-II will follow the same lines as the primary outcome. The analyses of safety parameters and all secondary endpoints will have an exploratory character.

**Pharmacological-toxicological evaluation**	Both Simvastatin and Escitalopram are approved for several indications since decades. The respective "latest version of German Fachinformation" for each medication are in the *Investigator site file* (ISF).
**Risks, adverse drug reactions, drug interactions, restrictions, contraindications, procedures in case of emergency**	Simvastatin is the IMP that will be tested versus placebo as add-on to standard antidepressant medication with Escitalopram, which will be given open label to each patient. We list the risks and potential adverse drug effects for both medications. See for further information the latest version of German "Fachinformation".<br><br>**Simvastatin:** Simvastatin is approved for several indications since decades and has in general proven to be a safe medication. The risks and adverse effects listed below are all taken from the latest version of German "Fachinformation".<br>The most prominent risk of statin use is myopathy, associated with muscle pain, muscle tenderness, muscle weakness and increases of creatine kinase (CK). The most severe form of myopathy is rhabdomyolysis with or without acute renal failure secondary to myoglobinuria that occurs very rarely and is dose-dependent. The combined incidence for myopathy/rhabdomyolysis in 41.413 patients treated for at least 4 years was 0.03% for 20mg/d, 0.08% for 40mg/d, and 0.61% for 80 mg/d Simvastatin. Thus, to minimize the risk of Simvastatin, we will use it in a medium dose (40mg/d) and not a high dose (80mg/d). In addition to safety laboratory values at screening, baseline, week 4, and week 12, It will be explicitly asked if the patient experienced unexplained muscle pain or dark urine as a sign of rhabdomyolysis.<br>Other adverse effects are listed below and the information is taken from the latest version of German "Fachinformation" and classified as follows:<br>*Very often: (>1/10); often (> 1/100); sometimes (> 1/1000), rarely (> 1/10.000), very rarely (< 1/10.000); not known (cannot be determined on basis of available data)*<br>Very often: none<br>Often: none<br>Sometimes: none<br>Rarely: Anemia, headache, dizziness, paresthesia, peripheral neuropathy, obstipation, stomach ache, flatulence, dyspepsia, diarrhea, nausea, emesis, pancreatitis, hepatitis/icterus, rash, pruritus, alopecia, myopathy, rhabdomyolysis, myalgia, muscle cramps, asthenia, vision blurred, visual impairment<br>Very rarely: insomnia, memory complaints, liver failure, lichenoid drug eruptions, muscle rupture, gynecomastia<br>Not known: depression, interstitial lung disease, tendinopathy, immune-mediated myopathy, erectile dysfunction<br><br>Medication that is contraindicated with Simvastatin due to inhibition of CYP3A4 will not be permitted (Itraconazol, Ketoconazol, Posaconazol, Voriconazol, Erythromycin, Clarithromycin, Telithromycin, HIV protease inhibitors (e.g. Nelfinavir), Boceprevir, Telaprevir, Nefazodon, Cobicistat, Ciclosporin, Danazol, Gemfibrozil).

	Further, <u>medication that is not recommended to use in parallel</u> to Simvastatin in a dosage of 40 mg/d will not be permitted (Fibrates, Amiodaron, Amlodipin, Verapamil, Fluconazol, Diltiazem, Fusidic acid, Niacin, Daptomycin, Lomitapid).<br><br>Ticagrelor is <u>not recommended</u> to use in parallel to Simvastatin in a dosage greater than 40 mg/d. Since in this study Simvastatin is given in a dosage of 40 mg/d, Ticagrelor will be permitted.<br><br>Finally, patients will be noticed that they should not drink grapefruit juice during Simvastatin treatment due to inhibition of CYP3A4 and corresponding increases in Simvastatin plasma levels.<br><br>In turn, <u>medication</u> that induces CYP3A4 leading to decreased Simvastatin levels will also <u>not be permitted</u> (Carbamazepine, Efavirenz, Nevirapin, Etravirine).<br><br>Patients with contraindications for Simvastatin will not be included or excluded from the study in case of later occurrence (see under inclusion criteria).<br><br>In case of an emergency the blind will be broken immediately and the patient will receive immediate medical care.<br><br>**<u>Escitalopram:</u>** The most prominent risk of Escitalopram is QTc-Prolongation in the ECG that can lead to ventricular arrhythmia (torsade des pointes). To exclude patients with QTc-prolongation an ECG will be conducted at screening. Furthermore, an additional safety ECG will be conducted at every visit.<br><br>Other adverse effects are listed below and the information is taken from the latest version of German “Fachinformation” and classified as follows:<br>*Very often: (>1/10); often (> 1/100); sometimes (> 1/1000), rarely (> 1/10.000), very rarely (< 1/10.000); not known (cannot be determined on basis of available data)*<br><br><u>Very often:</u> headache, nausea<br><br><u>Often:</u> decreased appetite, increased appetite, weight gain, anxiety, restlessness, nightmares, decreased libido, anorgasmia, insomnia, fatigue, dizziness, paresthesia, tremor, sinusitis, yawning, diarrhea, obstipation, emesis, dry mouth, hyperhidrosis, arthralgia, myalgia, delayed ejaculation, erectile dysfunction, fever<br><br><u>Sometimes:</u> weight loss, bruxism, agitation, nervousness, panic attacks, confusion, disturbed taste, insomnia, syncope, mydriasis, blurred vision, tinnitus, tachycardia, epistaxis, gastrointestinal bleeding, urticarial, alopecia, rash, pruritus, metrorrhagia, menorrhagia, edema<br><br><u>Rarely:</u> anaphylaxis, aggression, depersonalization, hallucination, serotonin syndrome, bradycardia<br><br><u>Very rarely:</u> none<br><br><u>Not known:</u> thrombocytopenia, inadequate ADH-secretion, hyponatremia, anorexia, mania, suicidality, dyskinesia, seizures, akathisia (psychomotor restlessness), QTc-prolongation in the ECG, orthostasis, hepatitis, increased liver enzymes, ecchymosis, angioedema, urinary retention, galactorrhea, priapism

	Medication that is contraindicated with Escitalopram will not be permitted, which includes all irreversible MAO-inhibitors and medication that is associated with QTc-prolongation [antiarrhythmica class IA and III, antipsychotics (e.g. haloperidol), phenothiazines, tricyclic antidepressants, antibiotics (e.g. moxifloxacin), and certain antihistaminergic drugs (e.g. astemizol, mizolastin)].<br>Patients with contraindications for Escitalopram will not be included (please see under inclusion criteria).
**Risk-benefit analysis**	Both medications (Simvastatin, Escitalopram) are approved for several indications since decades and have overall been proven to be safe. All depressed patients enrolled in the trial will be continuously treated with a gold standard antidepressant (Escitalopram) as described by the National Disease Management Guideline (“Nationale Versorgungsleitlinie”) for the treatment of MDD. For Escitalopram a dose-response relationship has been suggested. Especially for patients with severe depression the high standard dose of 20 mg/d was shown to be superior compared to 10 mg/d [1]. A fixed-dose of 10 mg/d Escitalopram for the entire study duration of 12 weeks would therefore not allow the optimal treatment for some patients. Thus, we consider this option ethically not justifiable. A dose increase from 10 mg/d to 20 mg/d based on individual patient response would however interfere with comparability of the treatment group and placebo group, because a strong decrease in depressive symptoms in the treatment group compared to placebo group could potentially be masked by a higher percentage of Escitalopram dose escalation in the placebo group. Therefore, we will increase Escitalopram to 20 mg/d after giving a low standard dose of 10 mg/d in the first two weeks. In case of adverse reactions related to Escitalopram, we will follow the instructions for dose adjustment described in 7.2.4 Treatment schedule / Dose regimen.<br>IMP Simvastatin is offered in an “add-on” design, thus there will be virtually no drug-free patients in this trial. Invasive interventions beyond ordinary sampling of peripheral venous blood will not be performed within this trial, therefore the risks associated with trial participation are mainly attributable to IMP Simvastatin that has already been shown in depressed patients to be safe and well tolerated in combination with an SSRI (Selective Serotonin Reuptake Inhibitor) [2].<br>It has been suggested that both for the lipid-lowering effects and for the pleiotropic effects of statins a high dose is more effective than a low dose [3-5]. Especially in studies examining effects of statins on diseases of the central nervous system such as multiple sclerosis and mild cognitive disorder a high dose of Simvastatin (60 – 80 mg/d) has been administered [6-8]. One of the most important side effects of Simvastatin is dose-dependent risk of myopathy. The combined incidence for myopathy/rhabdomyolysis under Simvastatin was 0.03% for 20 mg/d, 0.08% for 40 mg/d and markedly went up to 0.61% for 80 mg/d according to latest version of German “Fachinformation”. To minimize the risk of Simvastatin and examine the pleiotropic effects of Simvastatin in depression, we will use it in a medium dose (40 mg/d) and not a high dose (80 mg/d). In addition to assessing safety laboratory values including creatine kinase (CK) at screening, baseline, at week 4, week 8 and week 12, it will be explicitly asked during each visit if the patient experienced unexplained muscle pain or dark urine as a sign of rhabdomyolysis. Furthermore, we will not include

	particularly vulnerable patients (e.g. patients with renal impairment, untreated hypothyroidism or history of muscle toxicity under statins or fibrates) to our study. To exclude patients with QTc-prolongation an ECG will be conducted at screening. Furthermore, an additional safety ECG will be conducted at every visit.<br>According to a meta-analysis of small, randomized controlled trials, a beneficial antidepressive effect of Simvastatin is to be expected [2]. Many depressed patients with comorbid obesity might additionally benefit from Simvastatin in terms of primary prevention of cardiovascular disease.<br>In summary, the potential benefits of the study by far outweigh the risks associated with Escitalopram and Simvastatin. Importantly, a recent extensive literature review on parallel SSRI and statin use concluded the following: “Escitalopram, Citalopram, and Paroxetine are almost certain to be safe with all statins” [9].

2. Synopse (Deutsch)

**Titel der Studie**	Simvastatin als Zusatztherapie zur antidepressiven Medikation mit Escitalopram bei Patienten mit Depression und Adipositas: Eine multizentrische, randomisierte, doppelt-verblindete, Placebo-kontrollierte Studie (SIMCODE)
**Art des Vorhabens**	Randomisierte, Placebo-kontrollierte, doppelt-blinde Multicenterstudie mit Parallelgruppen- Design. Phase II
**Sponsor**	Charité – Universitätsmedizin Berlin, Chariteplatz 1, 10117 Berlin
**Sponsorvertreter und Prüfer / Leiter der Prüfgruppe (PI)**	Prof. Dr. Christian Otte<br>Charité – Universitätsmedizin Berlin, Klinik für Psychiatrie und Psychotherapie, Campus Benjamin Franklin, Hindenburgdamm 30, 12203 Berlin<br>Tel: 030-450517531; Fax: 030-450 517942<br>E-Mail: christian.otte@charite.de
**Administration**	NeuroCure Clinical Research Center (NCRC), Charité – Universitätsmedizin Berlin, Campus Mitte, Charitéplatz 1, 10117 Berlin<br>Koordinierungszentrum für Klinische Studien (KKS Charité), Charité – Universitätsmedizin Berlin, Campus Virchow-Klinikum, Augustenburger Platz 1, 13353 Berlin
**Prüfzentren**	Koordinierender Prüfer: Prof. Dr. Christian Otte, Charité – Universitätsmedizin Berlin, Klinik für Psychiatrie und Psychotherapie, Campus Benjamin Franklin<br>Prüfzentren: 9 Zentren in Deutschland.<br>Data and Safety Monitoring Board (DSMB):<br>- Prof. Dr. Brenda Penninx, Vrije Universiteit Amsterdam<br>- Prof. Dr. Falk Kiefer, Zentralinstitut für Seelische Gesundheit, Mannheim<br>- Prof. Dr. Carsten Spitzer, Fachklinik Universitätsmedizin Rostock<br>- Mrs. Francoise Margue, Deutsche Depressionsliga
**Zeitplan**	Rekrutierungsstart / Beginn der Studie (*first patient first visit*): Q1 2020<br>Ende der Rekrutierung: Q1 2023<br>Ende der Studie[2] (*last patient last visit*): Q2 2023<br>Studiendauer (für einen Patienten): 12 Wochen<br>Katamnese: keine
**Fragestellung aufbauend auf wissenschaftlichem Hintergrund**	Major Depression (MDD) und Adipositas gehören zu den häufigsten Erkrankungen weltweit. Statine gehören zu den meist verschriebenen Medikamenten und ihre Sicherheit und Effektivität ist etabliert. Statine werden empfohlen in der primären Prävention von kardiovaskulären Krankheiten, die sowohl mit MDD als auch mit Adipositas in Verbindung gebracht wurden. Darüber hinaus zeigte eine Metaanalyse, dass Statine außerdem vielversprechend als Ergänzungstherapie zur herkömmlichen antidepressiven Therapie sein könnten, da sich antidepressive Effekte zeigten.

[2] 2010/C 82/01, 2.5 43.

	Nichtsdestotrotz hat bis jetzt keine Studie die potentielle antidepressive Wirkung von Statinen bei Patienten mit MDD und komorbider Adipositas getestet.
**Studienziele**	**<u>Primäres Ziel</u>**<br>Untersuchung, ob eine Simvastatin (40 mg/d) Zusatztherapie zur antidepressiven Medikation mit Escitalopram (20 mg/d) zu einer zusätzlichen Verbesserung der Depression im Vergleich zu einer Zusatzbehandlung mit Placebo bei Patienten mit Major Depression und komorbider Adipositas führt<br>**<u>Sekundäres Ziel</u>**<br>Untersuchung, ob eine Simvastatin (40 mg/d) Zusatztherapie zur antidepressiven Medikation mit Escitalopram (20 mg/d) zu einer zusätzlich verbesserten Ansprechrate, Remissionsrate, Veränderungseinschätzung durch Patienten, Schwere- und Veränderungseinschätzung durch Behandler, Lebensqualität, Sozialem Funktionsniveau, selbstberichtete Depression, Lipidwerten und Immunometabolismus / mitochondrialer Funktion im Vergleich zur Zusatzbehandlung mit Placebo bei Patienten mit Majo Depression und komorbider Adipositas führt
**Hypothesen / Endpunkte**	**<u>Primäre Hypothese</u>**:<br>Simvastatin (40 mg/d) als Zusatztherapie zur konventionellen Antidepressiva-Standardtherapie mit Escitalopram (20 mg/d) führt zu einer zusätzlichen Verbesserung der Depression im Vergleich zu einer Zusatzbehandlung mit Placebo bei Patienten mit Major Depression und komorbider Adipositas<br>**<u>Primärer (konfirmatorischer) Endpunkt</u>**<br>Veränderungsscore von Baseline zur Woche 12 in der Montgomery-Asberg-Depression Rating Skala (MADRS)<br>**<u>Sekundäre Hypothesen:</u>**<br>Simvastatin (40 mg/d) als Zusatztherapie zur konventionellen Antidepressiva-Standardtherapie mit Escitalopram (20 mg/d) führt nach 12 Wochen zu einer zusätzlich verbesserten<br>**1)** Ansprech- und Remissionsrate<br>**2)** Veränderungseinschätzung durch Patienten, Schwere- und Veränderungseinschätzung durch Behandler, Lebensqualität, Sozialem Funktionsniveau, selbstberichtete Depression<br>**3)** Lipidwerte<br>**4)** Immunometabolismus / mitochondriale Funktion<br>im Vergleich zur Behandlung mit Placebo bei Patienten mit Major Depression und komorbider Adipositas<br>**<u>Sekundäre (explorative) Endpunkte (Effektivität)</u>**<br>**1)** Ansprechrate (Reduktion der Punkte im MADRS um 50 % zur Baseline), Remission (MADRS Punkte < 10) und MADRS geringster klinisch relevanter Unterschied (MCID)<br>**2)** Veränderung in den *Beck Depression Inventory* (BDI-II) Punkten von Baseline zu Woche 12 und geringster klinisch relevanter Unterschied (MCID)

	**<u>Weitere sekundäre (explorative) Endpunkte</u>**<br>**2)** Veränderung in der *Patients' Global Impression of Change Scale* (PGIC), Veränderung in der *Clinicians' Global Impression of Severity of illness* (CGI-S*), Clinicians' Global Impression of Improvement* (CGI-I),<br>*EuroQol-5 Dimensions-3 Levels Questionnaire* (EQ-5D-3L), *Social and Occupational Functioning Assessment Scale* (SOFAS) von Baseline zu Woche 12<br>**3)** Veränderung in *high-density lipoprotein* (HDL), *low-density lipoprotein* (LDL) und total Cholesterin von Baseline zu Woche 12<br>**4)** Veränderung in der respiratorischen Reserve von Monozyten und T Zellen und andere zelluläre Funktionen von Baseline zu Woche 12
**Studiendesign**	multizentrisch, randomiziert, doppel-blind, Placebo-kontrolliert, Parallelgruppen
**Studienmedikation / Behandlungsstrategie**	**<u>IMP:</u>** (*Investigational Medical Product*): Simvastatin (feste Dosierung 40 mg/d für 12 Wochen)<br>**<u>Standardmedikation</u>**: Escitalopram (Dosierung 10 mg/d in den ersten zwei Wochen, dann feste Dosierung 20 mg/d bis Woche 12)<br>Simvastatin oder Placebo werden als Zusatztherapie zur konventionellen Antidepressiva-Standardtherapie mit Escitalopram zur Verfügung gestellt.
**Vergleichstherapie**	Placebo-Kapseln (nicht unterscheidbar zur IMP) für 12 Wochen
**Gesamtzahl Patienten**	160 Patienten unterteilt in zwei Gruppen:<br>- Gruppe 1 (80 Patienten): Placebo plus Standardmedikation<br>- Gruppe 2 (80 Patienten): IMP plus Standardmedikation
**Studienpopulation**	Patienten mit komorbider Adipositas (body mass index ≥ 30) und Major Depression
**Einschlusskriterien**	- Eine unterschriebene Einwilligungserklärung ist vorhanden<br>- Der Patient ist einwilligungsfähig [Er erfasst klar Art, Bedeutung und Tragweite (Risiken) der ärztlichen Maßnahme]<br>- Der Patient hat eine Episode einer Majoren Depression nach DSM 5<br>- Der Patient hat einen *Montgomery-Asberg Depression Rating Scale* (MADRS) Score von ≥ 18<br>- Der Patient hat einen Body Mass Index von ≥ 30<br>- Das Alter des Patienten liegt zwischen 18 und 65 Jahren (≥ 18 und ≤ 65)<br>- Die Patientin hat in den vergangenen 6 Monaten nicht entbunden und befindet sich nicht in der Stillzeit<br>- Im Falle einer nicht-psychotropen Medikation:<br>Der Patient erhält eine stabile pharmakologische Medikation für mind. 14 Tage vor Studienbeginn (jegliche Veränderungen der Dosis oder Einnahmefrequenz müssen mit nein beantwortet werden)<br>- Der Patient hatte in den letzten 7 Tagen vor Studieneinschluss keine Einnahme von Antidepressiva (Das Absetzen einer wirksamen Vormedikation, um Patienten zur Studienteilnahme zu qualifizieren, ist ausdrücklich untersagt)<br>- Der Patient hat keine vorherige Behandlung mit Escitalopram in der aktuellen *Major depressive episode* (MDE)

	- Der Patient hatte weniger als drei (< 3) Behandlungsversuche mit Antidepressiva in der aktuellen MDE<br>- Der Patient besitzt keine frühere *Non-Response* auf Escitalopram<br>- Der Patient wurde in der aktuellen MDE nicht mit Ketamin, elektrokonvulsiver Therapie (EKT) oder anderen Stimulationsbehandlungen behandelt<br>- Der Patient erfüllt keine der folgenden Kriterien:<br>- Schizophrenie<br>- schizoaffektive Störung<br>- bipolare Störung<br>- Der Patient hat keine diagnostizierte Demenz und besitzt nach dem klinischen Eindruck keine moderaten oder schweren generellen kognitiven Funktionseinschränkungen<br>- Der Patient hat keine klinisch relevanten erhöhten Leberwerte [GOT oder GPT > 3 x obere Normgrenze (ULN)] und hat keinen erhöhten CDT-Wert (Carbohydrate-deficient Transferrin) ≥ 2,4 %<br>- Der Patient erfüllt nicht die Kriterien von Alkoholkonsumstörung (DSM-5: 303.90; ICD-10: F10.20) bzw. Substanzkonsumstörung (DSM-5: 304; ICD-10: F11.20 – F19.20) im M.I.N.I für DSM-5 und ein Urin oder Serum-Drogenscreening-Test ist negativ (ausgenommen Benzodiazepine und Opiate)<br>- Der Patient hat keine Suizidversuche in der Vorgeschichte<br>- Der Patient hat keine bekannte Epilepsie oder Blutungsneigung oder ein Engwinkelglaukom oder ein Glaukom in der Vorgeschichte<br>- Der Patient hatte keine bariatrische Chirurgie in der Vorgeschichte<br>- Der Patient besitzt keine bekannten Allergien oder Kontraindikationen bzgl. Escitalopram oder Simvastatin<br>- Keine der folgenden Punkte treffen für den Patienten zu:<br>- Erblich bedingte Muskelerkrankungen<br>- Bekannte Vorgeschichte von Rhabdomyolyse<br>- erhöhte Kreatininkinase (CK) außerhalb der geschlechtsspezifischen Referenzintervalle<br>- muskuläre Symptomatik unter Behandlung mit Statinen oder Fibraten in der Vorgeschichte<br>- Der Patient hat keinen erhöhten TSH-Wert (TSH basal) außerhalb der alters- und geschlechtsspezifischen Referenzintervallen<br>Der Patient hat keinen Insulin-abhängigen Diabetes Mellitus<br>- Der Patient besitzt keine unkontrollierte Leberfunktionsstörung, Nieren- oder Herz-Kreislauf-Erkrankungen<br>- Der Patient besitzt keine unbehandelte Hypothyreose<br>- Der Patient hatte keinen Myokardinfarkt oder Schlaganfall in der Vorgeschichte<br>- Der Patient besitzt keine symptomatische periphere arterielle Verschlusskrankheit<br>- Der Patient besitzt keine monogene familiäre Hyperlipidämie<br>- Der Patient hat keine klinisch signifikanten Auffälligkeiten im Sicherheitslabor<br>- Der Patient nimmt derzeit und nahm innerhalb der letzten 6 Monate an keiner anderen interventionellen Studie teil<br>- Der Patient ist kein Mitarbeiter eines Studienzentrums oder Familienmitglied eines Mitarbeiters oder eines Prüfers oder anderweitig abhängig vom Sponsor, von einem der Prüfer oder Prüfzentren.

**Ausschlusskriterien**	- Der Patient hat eine aktuelle Einnahme von Statinen (für Visiten 2-6 gilt der Zusatz: mit Ausnahme der IMP Simvastatin)<br>- Der Patient hat eine aktuelle Einnahme von Antidepressiva (für Visiten 2-6 gilt der Zusatz: mit Ausnahme der Standardmedikation Escitalopram)<br>- Beim Patienten bestehen akute suizidale Tendenzen (MADRS Item 10 > 4)<br>- Der Patient benutzt potente CYP3A4-Inhibitoren (z.B. Clarithromycin, Erythromycin, HIV Proteaseinhibitoren – siehe „Mögliche Risiken, Nebenwirkungen, Kontraindikationen, Maßnahmen, die bei eventuellen Zwischenfällen zu ergreifen sind“)<br>- Der Patient benutzt potente CYP3A4-Induktoren (Carbamazepine, Efavirenz, Nevirapin, Etravirine)<br>- Der Patient benutzt Fibrate, Amiodaron, Amlodipin, Verapamil, Fluconazol, Diltiazem, Fusidinsäure, Niacin, Daptomycin oder Lomitapid oder BCRP-Inhibtoren (z.B. Elbasvir oder Grazoprevir)<br>- Der Patient benutzt Gemfibrozil, Ciclosporin oder Danazol<br>- Der Patient hat eine bekannte Überempfindlichkeit gegen sonstige Bestandteile von Simvastatin und Escitalopram [Butylhydroxyanisol, mikrokristalline Cellulose, Citronensäure-Monohydrat, Stärke, Lactose, Magnesiumstearat, Hypromellose, Talkum, Titandioxid, Eisen(III)-oxid, hochdisperses Siliciumdioxid, Croscarmellose-Natrium, Macrogol (Polyethylenglycol)]<br>- Der Patient benutzt Medikation, die mit QTc-Verlängerung assoziiert ist [Antiarrhythmika Klasse IA and III, Antipsychotika (z.B. Haloperidol), Phenothiazine, trizyklische Antidepressiva, Antibiotika wie z.B. Moxifloxacin, und einige antihistaminerge Medikation (z.B. Astemizol, Mizolastin)]<br>- Der Patient hat klinisch signifikante Auffälligkeiten im 12-Kanal EKG (z.B. Verlängerung des QTc-Intervalls ≥ 500 ms oder QTc ≥ 60 ms zu Baseline-Visite)<br>- Die Patientin ist schwanger<br>- Die Patientin im gebärfähigen Alter zeigt eine fehlende Bereitschaft für die Anwendung einer effektiven Verhütungsmethode (definiert als Pearl-Index < 1)<br>- Der Patient nimmt psychotrope Medikation (z.B. Antipsychotika, Antikonvulsiva, Lithium oder Johanniskraut) mit Ausnahme von Benzodiazepinen, Non-Benzodiazepinen oder Opiaten ein<br>- Der Patient benutzt nicht selektive, irreversible MAO-Hemmer (z.B. Tranylcypromin) oder selektive, reversible MAO-A Hemmer (z.B. Moclobemid) oder den reversiblen nicht selektiven MAO-Hemmer Linezolid<br>- Der Patient zeigt eine fehlende Bereitschaft zur Speicherung, Verarbeitung und Weitergabe pseudonymisierter Studiendaten im Rahmen der klinischen Prüfung<br>- Der Patient ist aufgrund behördlicher oder gerichtlicher Anordnung in einer Anstalt untergebracht<br>- Der Patient hat eine aktive SARS-CoV-2-Infektion
**Visiten und Dokumentationszeitpunkte**	Für die Planung der einzelnen Visiten ist eine maximale Abweichung von ± 3 Tagen zulässig.

Erhebungen	Screening	Baseline<br>Visit 1<br>Woche 0	Visit 2<br>Woche 1	Visit 3<br>Woche 2	Visit 4<br>Woche 4	Visit 5<br>Woche 8	Studienende<br>Visit 6<br>Woche 12
**Screening und Einwilligung**							
Einwilligungserklärung	X						
Einschlusskriterien		X					
Ausschlusskriterien		X	X	X	X	X	X
Medizinische Anamnese	X						
Aktueller Behandlungsstatus		X					
M.I.N.I – DSM-5	X						
Urin/Serum Drogenscreening	X						
Begleitmedikation	X						
**Sicherheit**							
SARS-CoV-2 Antigen Schnelltest (für Patienten ohne Nachweis vollständiger Impfung)							
Körperliche Untersuchung	X						X
EKG	X	X	X	X	X	X	X
Blutdruck- und Herzfrequenzmessung	X	X	X	X	X	X	X
Sicherheitslabor (inkl. Schwangerschaftstest - nur für Frauen)	X	X			X	X	X
Registrierung *Adverse Events*			X	X	X	X	X
**Effektivität**							
MADRS	X	X	X	X	X	X	X
BDI-II		X	X	X	X	X	X
**Soz. Funktionsniveau und Lebensqualität**							
SOFAS		X			X		X
EQ-5D-3L		X			X		X
**Andere**							
Demographische Daten	X						
BMI	X	X					X

Bauchumfang	X					X
PGIC		X	X	X	X	X
CGI-S	X	X	X	X	X	X
CGI-I		X	X	X	X	X
Labor - Lipid	X					X
Labor - Immun-metabolismus / mitochondriale Funktion	X			X	X	X
Medikamenten-ausgabe	X		X	X	X	

Anmerkung: Elektrokardiographie (EKG), Body-Mass-Index (BMI), Mini International Neuropsychiatric Interview (MINI), Montgomery-Asberg Depression Scale (MADRS), Beck-Depressions-Inventar (BDI), Patient Global Impression of Change (PGIC), Clinicians' Global Impression of Severity of illness (CGI-S), Clinicians' Global Impression of Improvement (CGI-I), Social and Occupational Functioning Assessment Scale (SOFAS), Generic Quality of Life Questionnaire (EQ-5D-3L).

**Sicherheit**	Beurteilung der Sicherheit: Beurteilung des Blutdrucks und der Herzfrequenz zu jeder Visite. Beurteilung der Laborparameter - Sicherheitslabor (inklusive Schwangerschaftstest) zum Screening, Baseline, Woche 4, Woche 8 und Woche 12. Körperliche Untersuchung zu Screening und zum Studienende. EKG zum Screening und zu jeder weiteren Visite. Das Standardprozedere für die Meldung von „adverse events" wird angewendet. „Adverse events" werden ab der Visite 2 zu jeder Visite erfasst.
**Abbruchkriterien**	**Abbruchkriterien für individuelle Patienten**<br>Jeder Teilnehmer kann ohne Angabe von Gründen aus der Studie ausscheiden. Zwingende Gründe für einen Abbruch sind:<br>- Adverse Events (AE), klin. sign. auffällige Laborbefunde oder andere Krankheitsfälle, bei denen eine weitere Studienteilnahme dem Wohlergehen des Patienten schaden würde (siehe auch 11.1)<br>- Wenn Patienten Ausschlusskriterien erfüllen (entweder neu entwickelt oder nicht zuvor erkannt) die einer weitere Studienteilnahme ausschließen<br>**Abbruchkriterien für Studie**<br>Laut der deutschen Fachinformation für Simvastatin war die kombinierte Inzidenz für Myopathie/Rhabdomyolyse bei 41.413 Patienten, die mindestens 4 Jahre behandelt wurden, 0,08 % für die Dosierung von 40 mg pro Tag Simvastatin. Die schwere symptomatische Hyponatriämie (Serum Natrium [S_{Na}] <125 mmol/L) ist eine seltene, jedoch potenziell fatale Komplikation von Escitalopram. Symptome von Hyponatriämie reichen von Kopfschmerzen, Konzentrationsstörungen, Gedächtnisstörungen, Verwirrtheit, Schwäche, unsicherer Gang und Stand bis zu Halluzination, Synkope, Krampfanfälle, Koma, Atemstillstand und Tod. Daher werden wir die gesamte Studie vorzeitig abbrechen, wenn das Vorkommen von behandlungs-assoziierten SAE „Myopathie/Rhabdomyolyse" wie definiert in der deutschen Fachinformation für Simvastatin (Muskelschmerzen, -empfindlichkeit oder –schwäche verbunden mit Erhöhungen der Kreatinkinase (CK) über das 10-Fache des oberen Normwertes) oder das Vorkommen vom behandlungs-assoziierten SAE „schwere symptomatische Hyponatriämie" (Serum Natrium [S_{Na}] <125 mmol/L) jeweils bei mehr als 5 Studienteilnehmern auftritt. Weiterhin wird ein vorzeitiges Studienende in Erwägung gezogen, wenn neue wissenschaftliche Erkenntnisse verfügbar

	werden, welche die Risiko-Nutzen-Abwägung signifikant verändern. Sollten solche Erkenntnisse verfügbar werden, wird die Rekrutierung und Behandlung sofort unterbrochen. Eine finale Entscheidung bzgl. einer Weiterführung oder Beendigung der Studie wird durch den Sponsor/PI getroffen basierend auf der Empfehlung des *Data and Safety Monitoring Board* (DSMB).
**Statistische Auswertung**	**<u>Effektivität (Primärer, konfirmatorischer Endpunkt)</u>**<br>In der primären Analyse soll mithilfe von *Gaussian linear models for repeated measures* (s.g. MMRM) die MADRS Veränderung von der Baseline zur Woche 12 zwischen der Simvastatin- und Placebo-Gruppe verglichen werden. Hierbei sollen die Faktoren Intervention, Zentrum, Zeit (Woche 1, 2, 4, 8, und 12) und die Interaktion Intervention x Zeit berechnet werden. Als Kovariat werden die Baseline MADRS Punkte berücksichtigt. Die primäre zu analysierende Population ist die *intention-to-treat* (ITT) Population.<br>**<u>Effektivität (Sekundäre, explorative Endpunkte)</u>**<br>Die zentralen sekundären Endpunkte, die Ansprechrate in Prozent, Remissionsrate und MCID sollen mithilfe von logistischen Regressionsmodellen analysiert werden, mit Intervention und Zentrum als Faktoren und dem MADRS Baseline Score als Kovariate. Die Analyse von kontinuierlichen sekundären Endpunkten soll identisch mit der Analyse des primären Ergebnisses erfolgen. Die Analyse der Sicherheitsparameter und der sekundären Endpunkte soll einen exploratorischen Charakter haben.
**Pharmakologisch-toxikologische Prüfung**	Sowohl Simvastatin als auch Escitalopram sind für mehrere Indikationen seit Jahrzehnten zugelassene Medikamente. Die jeweiligen aktuellsten Versionen der deutschen Fachinformationen befinden sich im *Investigator site file* (ISF).
**Mögliche Risiken, Nebenwirkungen, Kontraindikationen, Maßnahmen, die bei eventuellen Zwischenfällen zu ergreifen sind**	Simvastatin ist das IMP (*Investigational Medical Product*), welches gegen ein Placebo als Zusatztherapie zur konventionellen Antidepressiva-Standardtherapie mit Escitalopram („open label“) jedem Teilnehmer verabreicht wird. Im Folgenden sind die Risiken und möglichen unerwünschten Ereignisse für beide Medikamente aufgelistet. Siehe für weitere Informationen die aktuellste Version der deutschen Fachinformation.<br><br>**<u>Simvastatin</u>**: Simvastatin ist seit mehreren Jahrzehnten für mehrere Indikationen zugelassen und ist als sichere Medikation anerkannt. Die folgenden Risiken und unerwünschten Ereignisse sind der aktuellsten Version der deutschen Fachinformation entnommen.<br>Simvastatin ruft gelegentlich eine Myopathie hervor, die sich in Muskelschmerzen, -empfindlichkeit oder –schwäche verbunden mit Erhöhungen der Kreatinkinase (CK) äußert. Die schwerwiegendste Form von Myopathie ist Rhabdomyolyse mit oder ohne akutem Nierenversagen aufgrund von Myoglobinurie, welche sehr selten vorkommt und dosisabhängig ist. Die kombinierte Inzidenz für Myopathie/Rhabdomyolyse in 41.413 Patienten, die mind. 4 Jahre behandelt wurde, war 0,03 % für 20 mg/d, 0,08 % für 40 mg/d, und 0,61 % für 80 mg/d Simvastatin. Um die Risiken von Simvastatin zu minimieren, werden wir die mittlere Dosis (40 mg/d) und nicht die hohe Dosis (80 mg/d) verwenden. Zusätzlich zu einem Sicherheitsla-

bor, welches zum Screening, zur Baseline, Woche 4 und Woche 12 ausgeführt wird, wird explizit danach gefragt, ob der Patient unter anderweitig unerklärlichen Muskelschmerzen oder dunklem Urin als Hinweis für Rhabdomyolyse gelitten hat/leidet.

Andere unerwünschte Ereignisse werden im Folgenden aufgelistet. Sie sind der aktuellsten Version der deutschen Fachinformation entnommen und wie folgt klassifiziert:

Sehr häufig (> 1/10), häufig (> 1/100), gelegentlich (> 1/1.000), selten (> 1/10.000), sehr selten (< 1/10.000), nicht bekannt (kann auf Grundlage der verfügbaren Daten nicht abgeschätzt werden)

Sehr häufig: keine

Häufig: keine

Gelegentlich: keine

Selten: Anämie, Kopfschmerzen, Parästhesien, Schwindel, periphere Neuropathie, Obstipation, Bauchschmerzen, Flatulenz, Dyspepsie, Diarrhö, Übelkeit, Erbrechen, Pankreatitis, Hepatitis/Ikterus, Hautausschlag, Juckreiz, Alopezie, Myopathie, Rhabdomyolyse, Myalgie, Muskelkrämpfe, Asthenie, verschwommenes Sehen, Sehverschlechterung

Sehr selten: Schlaflosigkeit, Beeinträchtigung des Erinnerungsvermögens, Leberversagen, lichenoide Arzneimittelexantheme, Muskelriss, Gynäkomastie

Nicht bekannt: Depression, interstitielle Lungenkrankheit, Tendinopathie, immunvermittelte nekrotisierende Myopathie, erektile Dysfunktion

Folgende Medikamente sind kontraindiziert bei Einnahme von Simvastatin, aufgrund der CYP3A4 Inhibition: (Itraconazol, Ketoconazol, Posaconazol, Voriconazol, Erythromycin, Clarithromycin, Telithromycin, HIV Protease Inhibitoren (z.B. Nelfinavir), Boceprevir, Telaprevir, Nefazodon, Cobicistat, Ciclosporin, Danazol, Gemfibrozil).

Folgende Medikamente, bei denen von einer gleichzeitigen Anwendung mit Simvastatin (Dosis 40 mg/d) abgeraten wird, sind nicht zugelassen (Fibrates, Amiodaron, Amlodipin, Verapamil, Fluconazol, Diltiazem, Fusidinsäure, Niacin, Daptomycin, Lomitapid).

Bei Ticagrelor wird von einer gleichzeitigen Anwendung mit Dosen von Simvastatin, die größer sind als 40 mg, abgeraten. Da im Rahmen dieser Studie Simvastatin in der Dosierung von 40 mg gegeben wird, ist Ticagrelor zugelassen.

Patienten werden außerdem darauf hingewiesen, dass sie keinen Grapefruitsaft im Zeitraum der Behandlung mit Simvastatin zu sich nehmen sollen, aufgrund der Inhibition von CYP3A4 und dem korrespondierenden Anstieg der Simvastatin-Plasmakonzentration.

Medikamente, welche CYP3A4 induzieren und demnach zu erniedrigten Simvastatin-Plasmalevel führen, werden auch nicht zugelassen (Carbamazepin, Efavirenz, Nevirapin, Etravirin).

Patienten, die eine Kontraindikation bzgl. Simvastatin aufweisen, werden nicht in die Studie eingeschlossen oder bei späterem Auftreten von der Studie ausgeschlossen (siehe Einschlusskriterien).

	Im Falle eines Notfalls wird die Verblindung unmittelbar aufgehoben, und der Patient erhält eine medizinische Versorgung.<br><br>**Escitalopram**: Das bedeutendste Risiko von Escitalopram stellt eine QTc-Verlängerung im EKG dar, welche zu ventrikuläre Arrhythmien (*Torsade de Pointes*) führen kann. Um Patienten mit einer QTc-Verlängerung auszuschließen, wird vor Studieneinschluss ein EKG geschrieben. Zusätzliche Sicherheits-EKGs werden bei jeder Visite ausgeführt.<br>Andere unerwünschte Ereignisse werden im Folgenden aufgelistet. Sie sind der aktuellsten Version der deutschen Fachinformation entnommen und wie folgt klassifiziert:<br>*Sehr häufig (> 1/10), häufig (> 1/100), gelegentlich (> 1/1.000), selten (> 1/10.000), sehr selten (< 1/10.000), nicht bekannt (kann auf Grundlage der verfügbaren Daten nicht abgeschätzt werden)*<br>Sehr häufig: Kopfschmerzen, Übelkeit<br>Häufig: Verminderter Appetit, gesteigerter Appetit, Gewichtszunahme, Ängstlichkeit, Ruhelosigkeit, anormale Träume, verringerte Libido, Anorgasmie, Schlaflosigkeit, Schläfrigkeit, Schwindel, Parästhesie, Tremor, Sinusitis, Gähnen, Diarrhoe; Obstipation; Erbrechen, Mundtrockenheit, Vermehrtes Schwitzen, Arthralgie, Myalgie, Ejakulationsstörungen, Impotenz, Fieber<br>Gelegentlich: Gewichtsabnahme, Nächtliches Zähneknirschen, Agitiertheit, Nervosität, Panikattacken, Verwirrtheit, Geschmacksstörungen, Schlafstörungen, Synkope, Mydriasis, Sehstörungen, Tinnitus, Tachykardie, Nasenbluten, Gastrointestinale Blutungen, Urtikaria, Haarausfall, Ausschlag, Juckreiz, Metrorrhagie, Menorrhagie, Ödeme<br>Selten: Anaphylaktische Reaktion, Aggression, Depersonalisation, Halluzinationen, Serotonin-Syndrom, Bradykardie,<br>Sehr selten: keine<br>Nicht bekannt: Thrombozytopenie, Inadäquate ADH-Sekretion, Hyponatriämie, Anorexie, Manie, suizidale Gedanken, suizidales Verhalten, Dyskinesien, Bewegungsstörungen, Krämpfe, psychomotorische Unruhe/Akathisie, Elektrokardiogramm: QT-Verlängerungen im EKG, Orthostatische Hypotonie, Hepatitis, erhöhte Leberenzyme, Ekchymosen, Angioödem, Harnretention, Galaktorrhoe, Priapismus,<br>Medikation die bei Einnahme von Escitalopram kontraindiziert ist, wird nicht zugelassen, darunter alle irreversiblen MAO-Inhibitoren und Medikamente die mit QT-Verlängerungen assoziiert sind [Antiarrhythmika Klasse IA und III, Antipsychotika (z.B. Haloperidol), Phenothiazin, trizyklische Antidepressiva, Antibiotika (z.B. Moxifloxacin) und bestimmte Antihistaminika (z.B. Astemizol, Mizolastin)].<br>Patienten, die eine Kontraindikation bzgl. Escitalopram aufweisen, werden nicht in die Studie eingeschlossen bzw. bei späterem Auftreten aus der Studie ausgeschlossen (siehe Ausschlusskriterien).
**Risiko-Nutzen-Abwägung**	Beide Medikamente (Simvastatin, Escitalopram) sind seit Jahrzehnten für mehrere Indikationen zugelassen und haben sich insgesamt als sicher erwiesen. Alle depressiven Patienten, die in die Studie eingeschlossen sind, werden kontinuierlich mit einem Standard-Antidepressivum (Escitalopram) behandelt, welches durch die Nationale Versorgungsleitlinie als

	„Goldstandard“ für die Behandlung von MDD beschrieben wurde. Es gibt Hinweise für eine Dosis-Wirkungs-Beziehung von Escitalopram. Insbesondere konnte für Patienten mit einer schweren Depression gezeigt werden, dass sie von der höheren Standarddosierung von Escitalopram 20 mg/d profitieren [1]. Eine fixe Dosierung von 10 mg/d Escitalopram für die gesamte Studienteilnahme von 12 Wochen würde daher einem Teil der Patienten nicht die bestmögliche Therapie ermöglichen und wäre aus unserer Sicht ethisch nicht vertretbar. Eine Aufdosierung von 10 mg/d auf 20 mg/d je nach individuellem Ansprechen würde hingegen die Vergleichbarkeit der Verumgruppe mit der Plazebogruppe potentiell beeinträchtigen, da eine stärkere Abnahme der depressiven Symptomatik in der Verumgruppe im Vergleich zur Plazebogruppe möglicherweise durch den vergleichsweise höheren Anteil an Escitalopram-Aufdosierungen in der Plazebogruppe maskiert werden würde. Daher werden wir die Dosierung nach initialer Dosierung von 10 mg/d in den ersten beiden Wochen auf 20 mg/d erhöhen bis zum Studienende. Dennoch ist bei Auftreten von Verträglichkeitsproblemen eine langsamere Aufdosierung bzw. eine Dosisreduktion möglich mit folgendem Dosisänderungsschema (siehe Kapitel 7.2.4).<br><br>Simvastatin als IMP wird als Zusatztherapie verabreicht, sodass keine medikamentenfreien Patienten in der Studie eingeschlossen sind. Invasive Interventionen, zusätzlich zur Entnahme von venösem Blut, werden in dieser Studie nicht angewandt. Die Risiken, die mit einer Studienteilnahme einhergehen, beziehen sich dementsprechend hauptsächlich auf Simvastatin als IMP, welches bereits bei depressiven Patienten eingesetzt wurde und sich in Kombination mit SSRIs (*Selective Serotonin Reuptake Inhibitor*) als gut verträglich erwiesen hat [2].<br><br>Sowohl für die lipidsenkende Wirkung als auch für die pleiotropen Effekte von Statinen gibt es Hinweise, dass eine hohe Statindosis effektiver ist als die niedrige Standarddosis [3-5]. Insbesondere bei ZNS-Erkrankungen wie Multiple Sklerose und leichter kognitiver Störung wurde in bisherigen Studien die hohe Dosis von Simvastatin (60 – 80 mg/d) verabreicht [6-8]. Eine der wichtigsten Nebenwirkungen von Simvastatin ist die dosisabhängige Myopathie. Die Myopathiehäufigkeit unter Simvastatin lag annährend bei 0,03 % unter 20 mg/d, bei 0,08 % unter 40 mg/d und steigt erst unter 80 mg/d deutlich an auf 0,61 % laut aktuellster Version der deutschen Fachinformation. Um die Risiken von Simvastatin zu minimieren und gleichzeitig die pleiotropen Effekte von Simvastatin bei Depression zu untersuchen, werden wir eine mittlere Dosis von 40 mg/d und nicht eine hohe Dosis von 80 mg/d verabreichen. Zusätzlich erheben wir Sicherheitsparameter inklusive Kreatininkinase (CK) im Blut zum Screening, zur Baseline und jeweils in Woche 4, 8 und 12. Ferner wird der Patient zu jeder Visite, explizit nach unerklärlichen Muskelschmerzen und dunklem Urin gefragt als Zeichen für Rhabdomyolyse. Weiterhin schließen wir besonders gefährdete Patientengruppen (z.B. Patienten mit Nierenfunktionsstörung, unbehandelter Hypothyreose oder muskulärer Symptomatik unter Behandlung mit Statinen oder Fibraten in der Anamnese) per Ein- und Ausschlusskriterien nicht in die Studie ein. Um Patienten mit einer QTc-Verlängerung auszuschließen, wird ein Sicherheits-EKG zum Screening und bei jeder anderen Visite ausgeführt.

	Basierend auf den Ergebnissen einer kürzlich erschienenen Metaanalyse, welche kleine RCTs umfasste, ist ein vorteilhafter antidepressiver Effekt von Simvastatin zu erwarten [2]. Viele Patienten mit komorbider Adipositas könnten zusätzlich, hinsichtlich einer primären Prävention von kardiovaskulären Krankheiten, von der Einnahme von Simvastatin profitieren.<br>Zusammenfassend überwiegt der potentielle Nutzen der Studie deutlich den potentiellen Risiken, die bei einer Einnahme von Simvastatin und Escitalopram auftreten könnten. Besonders zu betonen sind die Ergebnisse einer kürzlich erschienen Literaturrecherche zum parallelen Gebrauch von SSRIs und Statinen mit folgendem Fazit: „Escitalopram, Citalopram und Paroxetin sind beinahe zweifelsfrei sicher in Kombination mit allen Statinen“ [9].

3. Flow-Chart

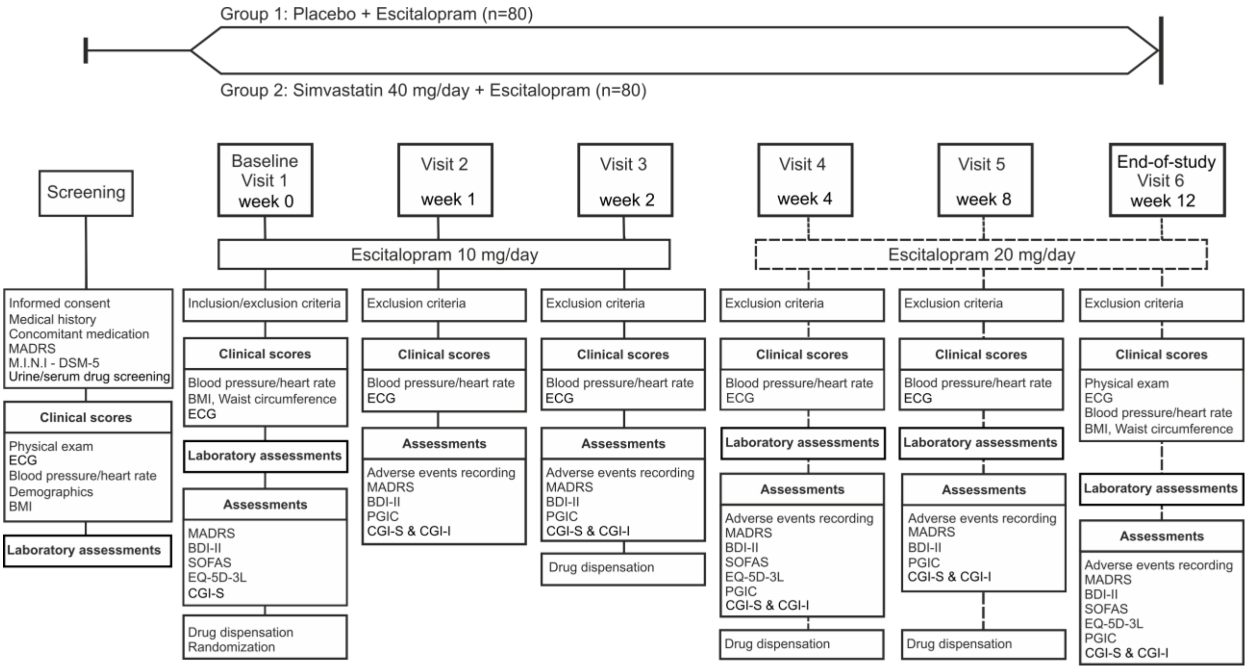

Figure 1: *Study procedure*

4. Introduction

4.1 Introduction and background

The World Health Organization's Global Burden of Disease (GBD) study just recently demonstrated that of all diseases worldwide, major depressive disorder (MDD) is among the top 5 with regard to years lived with disability (YLD) [10]. Among all psychiatric disorders, MDD is by far the leading cause of disability [11]. The 12-months prevalence of MDD in Germany is 6% (women: 8.1%; men: 3.8%) [12]. Mortality rates of MDD are twice as high compared to the general population and translate to a reduced life expectancy of 14.0 years in men and 10.1 years in women [13].

The prevalence of obesity in the German population is 23.3% in men and 23.9% in women [14]. In patients with obesity, mortality rates are elevated and they increase in relation to BMI as recently confirmed in a meta-analysis of 239 studies from four continents [15]. In the GBD study, obesity was estimated to cause 3.4 million deaths, 3.9% of years of life lost, and 3.8% of disability-adjusted life years worldwide [16].

MDD and obesity are both linked to a higher risk of cardiovascular disease and stroke, further increasing their public health and economic impact. Importantly, MDD and obesity frequently co-occur and the presence of one condition increases the risk for developing the other [17].

Statins (3-hydroxy-3-methylglutaryl Co-A reductase inhibitors) are among the most prescribed medications worldwide with well-established safety and efficacy. Recent guidelines recommend use of statins in primary prevention of cardiovascular disease [18], which has been linked to both MDD and obesity.

Moreover, statins are highly promising candidates to treat MDD as described in detail below. However, no randomized controlled study so far has tested the antidepressive potential of statins in patients with MDD and comorbid obesity. Importantly, this is a difficult-to-treat population that often exhibits a chronic course of MDD and treatment resistance [19-22]. Given the enormous public health impact of MDD and obesity and given the evidence of antidepressive effects of

statins, a controlled trial of adjunct statin treatment in comorbid patients appears clearly warranted.

4.2 Data on IMP-Application

4.2.1 Summary

Simvastatin is a lipid-altering agent and one of the components of multiple risk factor intervention in individuals at significantly increased risk for atherosclerotic vascular disease due to hypercholesterolemia. Simvastatin administration is indicated as an adjunct to diet when the response to a diet restricted in saturated fat and cholesterol and other nonpharmacologic measures alone has been inadequate. In patients with coronary heart disease (CHD) or at high risk of CHD, Simvastatin can be started simultaneously with diet. The indications listed below are taken from the prescribing information for Simvastatin (“Fachinformation SimvaHEXAL®, aktuellste Version”).

- Reductions in Risk of CHD Mortality and Cardiovascular Events. Simvastatin is indicated to
  - Reduce the risk of total mortality by reducing CHD deaths.
  - Reduce the risk of non-fatal myocardial infarction and stroke.
  - Reduce the need for coronary and non-coronary revascularization procedures.
- Hyperlipidemia. Simvastatin is indicated to
  - Reduce elevated total cholesterol (total-C), low-density lipoprotein cholesterol (LDL-C), apolipoprotein B (ApoB), and triglycerides (TG), and to increase high-density lipoprotein cholesterol (HDL-C) in patients with primary hyperlipidemia (Fredrickson type IIa, heterozygous familial and nonfamilial) or mixed dyslipidemia (Fredrickson type IIb).
  - Reduce elevated TG in patients with hypertriglyceridemia (Fredrickson type lV hyperlipidemia).
  - Reduce elevated TG and VLDL-C in patients with primary dysbetalipoproteinemia (Fredrickson type lll hyperlipidemia).
  - Reduce total-C and LDL-C in patients with homozygous familial hypercholesterolemia (HoFH) as an adjunct to other lipid-lowering treatments (e.g., LDL apheresis) or if such treatments are unavailable.
- Adolescent Patients with Heterozygous Familial Hypercholesterolemia (HeFH). Simvastatin is indicated as an adjunct to diet to reduce total-C, LDL-C, and Apo B levels in adolescent boys and girls who are at least one-year post-menarche, 10-17 years of age, with HeFH, if after an adequate trial of diet therapy the following findings are present:
  - LDL cholesterol remains ≥190 mg/dL; or
  - LDL cholesterol remains ≥160 mg/dL and
  - There is a positive family history of premature cardiovascular disease (CVD) or
  - Two or more other CVD risk factors are present in the adolescent patient.

In patients with CHD or at high risk of CHD, Simvastatin can be started simultaneously with diet. For patients at high risk for a CHD event due to existing CHD, diabetes, peripheral vessel disease, history of stroke or other cerebrovascular disease, the recommended starting dose is 40 mg/d. Lipid determinations should be performed after 4 weeks of therapy and periodically thereafter.

Due to the increased risk of myopathy, including rhabdomyolysis, particularly during the first year of treatment, use of the 80 mg/d dose of Simvastatin should be restricted to patients who have been taking Simvastatin 80 mg/d chronically (e.g., for 12 months or more) without evidence of muscle toxicity. Patients who are currently tolerating the 80 mg/d dose of Simvastatin who need

to be initiated on an interacting drug that is contraindicated or is associated with a dose cap for Simvastatin should be switched to an alternative statin with less potential for the drug-drug interaction. Due to the increased risk of myopathy, including rhabdomyolysis, associated with the 80 mg/d dose of Simvastatin, patients unable to achieve their LDL-C goal utilizing the 40 mg/d dose of Simvastatin should not be titrated to the 80 mg/d dose, but should be placed on alternative LDL-C-lowering treatment(s) that provides greater LDL-C lowering.

For adverse drug reactions and restrictions, contraindications and drug interactions see 7.2.5.

4.2.2 Physical, Chemical, and Pharmaceutical Properties and Formulation

Simvastatin is a lipid-lowering agent that is derived synthetically from a fermentation product of Aspergillus terreus. After oral ingestion, Simvastatin, which is an inactive lactone, is hydrolyzed to the corresponding β -hydroxyacid form. This is an inhibitor of 3-hydroxy-3-methylglutaryl-coenzyme A (HMG-CoA) reductase. This enzyme catalyzes the conversion of HMG-CoA to mevalonate, which is an early and rate-limiting step in the biosynthesis of cholesterol.

Simvastatin is butanoic acid, 2,2-dimethyl-,1,2,3,7,8,8a-hexahydro-3,7-dimethyl-8-[2-(tetrahydro-4- hydroxy-6-oxo-2H-pyran-2-yl)-ethyl]-1-naphthalenyl ester, [1S-[1α,3α,7β ,8β (2S*,4S*),-8aβ]]. The empirical formula of Simvastatin is C25H38O5 and its molecular weight is 418.57. Its structural formula is:

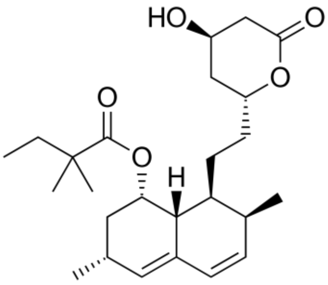

Simvastatin is a white to off-white, nonhygroscopic, crystalline powder that is practically insoluble in water, and freely soluble in chloroform, methanol and ethanol. Simvastatin for oral administration contain the following inactive ingredients: ascorbic acid, citric acid, hydroxypropyl cellulose, hypromellose, iron oxides, lactose, magnesium stearate, microcrystalline cellulose, starch, talc, and titanium dioxide. Butylated hydroxyanisole is added as a preservative.

Simvastatin should be stored between 5-25°C.

4.2.3 Nonclinical Studies

Simvastatin is a prodrug and is hydrolyzed to its active β-hydroxyacid form, simvastatin acid, after administration. Simvastatin is a specific inhibitor of 3-hydroxy-3-methylglutaryl-coenzyme A (HMG-CoA) reductase, the enzyme that catalyzes the conversion of HMG-CoA to mevalonate, an early and rate limiting step in the biosynthetic pathway for cholesterol [23]. The primary mechanism of action is the lowering of serum cholesterol through inhibiting hepatic cholesterol biosynthesis thereby upregulating the hepatic low-density lipoprotein (LDL) receptors and increasing the clearance of LDL-cholesterol (LDL-C) [24]. Simvastatin reduces very low-density lipoprotein (VLDL) and triglyceride (TG) and increases high-density lipoprotein cholesterol (HDL-C) [23].

In addition, Simvastatin may exert cardiovascular protective effects that are independent of LDL-C lowering called “pleiotropic” effects. Because statins inhibit the production of isoprenoid intermediates in the cholesterol biosynthetic pathway, the post-translational prenylation of small guanosine triphosphate binding proteins such as Rho and Rac, and their downstream effectors such as Rho kinase and nicotinamide adenine dinucleotide phosphate oxidases are also inhibited. In cell culture and animal studies, these effects alter the expression of endothelial nitric oxide synthase, the stability of atherosclerotic plaques, the production of pro-inflammatory cytokines and

reactive oxygen species, the reactivity of platelets, and the development of cardiac hypertrophy and fibrosis. [24]

Simvastatin has antidepressant and anxiolytic effects in rats [25]. In addition, statin effects in animal models on several neurobiological processes and structures with relevance for MDD such as inflammatory markers, glucocorticoids and serotonin in the hippocampus, glutamatergic N-Methyl-D-Asparte (NMDA) receptors, Brain Derived Neurotrophic Factor (BDNF) levels, and dopamine receptors have been demonstrated (for review of CNS effects of statins see [26]).

Oral absorption of Simvastatin is rapid and the bioavailability low due to an extensive first-pass metabolism. Plasma protein binding is high for Simvastatin (> 95%). Simvastatin undergoes enterohepatic circulation and is recovered mainly in feces via bile, the extent of which is species-dependent. Elimination is rapid with metabolism being the main elimination route. Metabolism varies with the animal species, particularly the beta-oxidation of the dihydroxy heptanoic side chain that occurs primarily in rodents. [27] The IC_{50} (the concentration resulting in 50% inhibition of cholesterol synthesis) value of Simvastatin varies from 1.0 to 23 nM in different experimental systems. [23]

The administration of high dosages of Simvastatin has resulted in the production of toxicity in a variety of tissues and in a number of species. However, the investigative toxicology studies have indicated that many are mechanism-based phenomena occurring only at high dosage levels of Simvastatin and are the product of a profound, prolonged inhibition of the target enzyme that is not achieved at clinical dosages in humans. [28] On the other hand, muscle injury upon statin treatment has been confirmed and it has been shown that it can be prevented in a rat model by giving mevalonate, the product of the inhibited enzyme [29]. Simvastatin was not teratogenic in rats or rabbits, but in several teratology studies in the 1980s Lovastatin was found to produce fetal malformations in rats and atorvastatin at maternally toxic doses was linked to developmental toxicity [30-32].

4.2.4 Effects in humans

Elevated levels of serum cholesterol is associated with the development of atherosclerosis and increased cardiovascular risk [33]. Simvastatin decreases cholesterol biosynthesis and decreases serum LDL-C and triglyceride levels [34]. Landmark clinical trials have demonstrated the efficacy of Simvastatin for both primary and secondary prevention of coronary heart disease (CHD) [35]. In addition, there is cumulative evidence for pleiotropic effects in humans but its contribution to clinical outcomes in addition to LDL-C lowering remains unknown [24].

Simvastatin showed antidepressive effects as adjunct treatment to Fluoxetin in one recent RCT [36] (see Figure 2). With regard to lipid levels, however, no change was reported during the course of the study (6 weeks). Concerning this, the effect of statin for prevention of depression was found to be independent of the cholesterol lowering effects of statins [37]. Furthermore, Simvastatin was superior to atorvastatin in depressed patients with heart disease [38]. According to a meta-analysis of small, randomized controlled trials with various statins, a beneficial antidepressive effect of Simvastatin is to be expected [2] (see Figure 3). In a recent Swedish national cohort study that included all individuals who were dispensed statins and were aged 15 years or older between 2006 and 2013, statin use was associated with reduced diagnoses of clinical depression [39]. However, effects of Simvastatin on depressed patients with comorbid obesity is not known to our knowledge, but it is to be expected that they might additionally benefit from Simvastatin in terms of primary prevention. Simvastatin passes the blood-brain barrier [40].

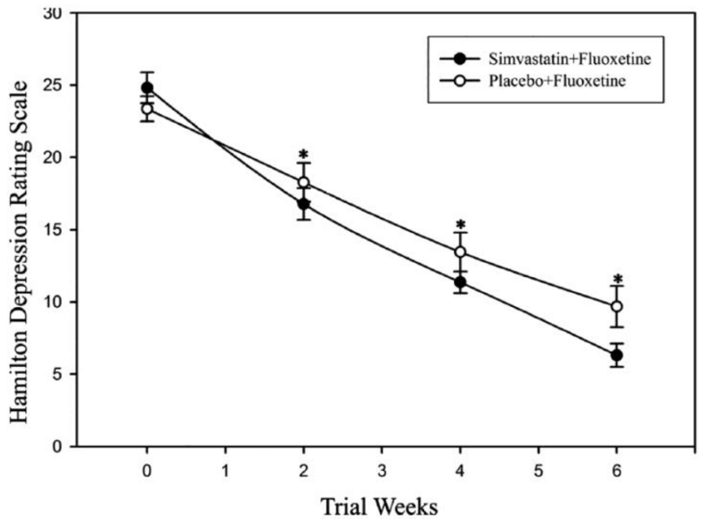

Figure 2*: Randomized controlled trial examining the effect of add-on Simvastatin vs. add-on placebo to Fluoxetine in patients with major depression (Gougol et al, J Psychopharmacol, 2015)[36].*

Simvastatin undergoes oxidative metabolism, primarily by cytochrome P450 3A4, while simvastatin acid is oxidised via CYP 3A4 and β-oxidation enzyme systems. Both the parent lactone form and the acid are normally present in very small amounts in plasma due to a high hepatic extraction ratio (~ 95%). The major active metabolites of simvastatin present in human plasma are the β-hydroxyacid of simvastatin and its 6´-hydroxy, 6´-hydroxymethyl, and 6´-exomethylene derivatives. Peak plasma concentrations of both active and total inhibitors were attained within 1.3 to 2.4 hours postdose [32]. Simvastatin is cleared predominantly by the liver. Nevertheless, the plasma concentration of HMG-CoA reductase inhibitory activity is probably higher for Simvastatin in patients with severe renal insufficiency as it is for Lovastatin. Although simvastatin is a substrate for CYP 3A4 simvastatin 80 mg inhibited neither hepatic nor intestinal CYP 3A4. Therefore, simvastatin can be given with other substrates of CYP 3A4 without concern over effects on their plasma levels. There is also no evidence that simvastatin inhibits other cytochrome isoforms. Furthermore, simvastatin is not known to cause any clinically significant change in the plasma levels of any other drug, regardless of its metabolism and clearance pathways. In addition, the plasma concentrations of simvastatin and its metabolites are too low to cause significant protein binding displacement reactions. [41]

CYP 3A4 inhibitors can produce substantial increases in the plasma concentrations of the parent lactone form and the equivalent acid, although the absolute levels remain quite low. However, there are, in addition, three downstream oxidised active metabolites of simvastatin that together account for about 75% of the HMG-CoA reductase inhibitory activity in plasma. The only known consequence of drug interactions between certain CYP 3A4 inhibitors and simvastatin is an increase in the risk of myopathy. [41]

Except for rare cases of myopathy and marked but asymptomatic increases in hepatic transaminases, none of the adverse effects of simvastatin found in animals occur at human therapeutic doses [41]. The most severe form of myopathy is rhabdomyolysis with or without acute renal failure secondary to myoglobinuria that occurs very rarely and is dose-dependent. The combined incidence for myopathy/rhabdomyolysis in 41.413 patients treated for at least 4 years was 0.03% for 20 mg/d, 0.08% for 40 mg/d, and 0.61% for 80 mg/d Simvastatin [32]. In regard to drug interactions with SSRI a recent extensive literature review concluded the following: “Escitalopram, Citalopram, and Paroxetine are almost certain to be safe with all statins” [9]. The concomitant use of SSRIs and statins was associated with a significantly lower risk for psychiatric hospital contacts due to depression and all-cause mortality and suicidal behavior [42]. Increasing age, female gender, renal insufficiency, subclinical hypothyroidism, polypharmacy, high levels of physical activity, alcohol and drug abuse, HIV infection, severe trauma, surgery with increased metabolic demands, genetic factors and inherited mitochondrial diseases have been related to

increased risk of statin-induced myopathy [43]. In a meta-analysis of randomized trials, statin therapy was found to be associated with a slightly increased risk of development of diabetes, but the risk was considered low both in absolute terms and when compared with the reduction in coronary events [44]. A systematic review on the risks of statins in pregnancy couldn't show a clear relationship of congenital anomalies with statin use in pregnancy and proposed that statins are probably not teratogenic [45].

4.2.5 Summary of Data and Guidance for the Investigator

Simvastatin is approved for several indications since decades and has in general proven to be an effective and safe medication in primary prevention of cardiovascular disease [18], which has been linked to both MDD and obesity. Preclinical and clinical data suggest that adjunctive treatment with statins could be useful for the treatment of depressive symptoms. Therefore, in our study, we want to investigate whether add-on Simvastatin to standard antidepressant medication improves depression to a greater extent than adjunct placebo in patients with major depression and comorbid obesity.

In randomized trials, statin therapy appears to cause only a slight increased risk of side effects compared with placebo, and no increased risk of discontinuation of therapy compared with placebo [46]. In 2017, a meta-analysis of 22 placebo-controlled trials on statin use involving nearly 130,000 participants showed that 13.3 % of subjects receiving a statin discontinued the drug compared with 13.9 % of subjects on placebo (odds ratio [OR] 0.99, 95% CI 0.93-1.06) over a mean follow-up of 4.1 years [47]. The most prominent risk of statin use is myopathy, associated with muscle pain, muscle weak-ness and increases of creatine kinase (CK). The most severe form of myopathy is rhabdomyolysis with or without acute renal failure secondary to myoglobinuria that occurs very rarely and is dose-dependent. Statin-induced myopathy appears to have a multi-factorial etiology that includes the effects that statins have on myocyte metabolism, the use of other concomitant drugs that increase statin blood levels, and genetic variables that predispose some patients to myopathy. Still, the risk-to-benefit ratio for most dyslipidemic patients greatly favors treatment with statins [48]. In regard to drug interaction with our standard medication Escitalopram, no clinically significant pharmacokinetic interaction is expected. Nevertheless, due to the increased risk of muscle injury and the corresponding warning from the US Food and Drug Administration we will be using a dose of 40 mg/d Simvastatin in our study. Because in pregnancy Simvastatin is contraindicated, pregnant or breastfeeding women or women with childbearing potential without acceptable form of contraception will not be included in the study and a highly sensitive serum HCG test will be conducted at screening visit, baseline, week 4, week 8 and at the end-of-study for women of childbearing potential.

4.3 <u>Rationale</u>

Evidence for antidepressive effects of statins has accumulated from four independent lines of research:

a) A meta-analysis [2] (see Figure 3) of three small pilot randomized controlled trials has found antidepressive effects of statins as adjunct therapy to standard antidepressant treatment and concluded: "Our results suggest that adjunctive treatment with statins could be useful for the treatment of depressive symptoms. Additional double-blind randomized, placebo-controlled trials are necessary to settle the matter" [2].

    Importantly, even though these pilot RCTs have shown antidepressive effects of statins, all of these studies suffered from small sample sizes and all of these studies were conducted in the same county strongly limiting the generalizability of these findings. Thus, we propose to conduct this confirmative RCT including about the same number of de-

pressed patients as in all previous RCTs combined. No competing study is currently registered at clinicaltrials.gov and the International Clinical Trials Registry Platform of the World Health Organization.

Study or Subgroup	Statin Mean	Statin SD	Statin Total	Placebo Mean	Placebo SD	Placebo Total	Weight	Std. Mean Difference IV, Random, 95% CI	Year
Ghanizadeh et al., 2013	16.32	5.04	30	20.4	5.48	31	36.8%	−0.76 [−1.29, −0.24]	2013
Haghighi et al., 2014	19.63	3.16	30	22.03	3.58	30	36.6%	−0.70 [−1.22, −0.18]	2014
Gougol et al., 2015	6.5	7.1	22	11.31	5.89	22	26.7%	−0.72 [−1.34, −0.11]	2015
**Total (95% CI)**			**82**			**83**	**100.0%**	**−0.73 [−1.05, −0.41]**	

Heterogeneity: $Tau^2 = 0.00$; $Chi^2 = 0.03$, df = 2 (P = 0.99); $I^2 = 0\%$
Test for overall effect: Z = 4.53 (P < 0.00001)

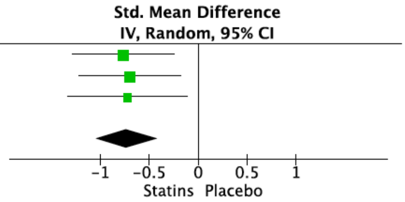

Figure 3: *Meta-analysis of three randomized controlled trials examining the effects of add-on statin vs. add-on placebo to SSRI in patients with major depression (Salagre et al, J Affect Dis, 2016)[4].*

b) A nationwide population-based registry study that included all incident SSRI users in Denmark between 1997 and 2012 (n = 872.000) found that combined use of SSRI and statins (n = 113.000) was associated with a substantially reduced risk of MDD (adjusted hazard ratio 0.64, 95% confidence interval 0.55 – 0.75) [49]. Importantly, Citalopram and Simvastatin was the most often used SSRI/statin combination and the authors concluded: "this specific combination would be a good candidate for a head-to-head test against a Citalopram-placebo combination in a randomized controlled trial". We will test the recommended combination in our study except using Escitalopram instead of Citalopram due to the lack of weight gain with Escitalopram compared to Citalopram [50].

c) A meta-analysis of prospective cohort studies [51] showed that statin users are less likely to develop MDD than non-users. One of the studies included in this meta-analysis was conducted by the first applicant and found odds of MDD to be decreased by 38% in statin users vs. non-users [52]. These results were recently corroborated by two large Danish nation-wide population-based studies, which found that statin users were less likely to develop depression after acute coronary syndrome [53] or after stroke compared to non-users of statins [54]

d) In animal models of MDD, antidepressive effects of statins have clearly been demonstrated. These studies have additionally confirmed statin effects on several neurobiological processes and structures with relevance for MDD such as inflammatory markers, glucocorticoids and serotonin in the hippocampus, glutamatergic N-Methyl-D-Asparte (NMDA) receptors, Brain Derived Neurotrophic Factor (BDNF) levels, and dopamine receptors (for review of CNS effects of statins see [26]).

In summary, given a) the enormous public health impact of depression [55, 56] and obesity [15, 16], b) the lack of remission in about 30% of depressed patients even after several antidepressive treatment attempts [57], c) the fact that obese MDD patients are especially prone to chronicity and non-response [19-21], d) the evidence of antidepressive effects of statins from four different sources (animal studies [26], meta-analysis of prospective cohort studies [51], meta-analysis of pilot controlled trials [2], nationwide cohort study [49]) and e) the established safety of statins, a confirmative controlled trial of add-on statin treatment in patients with MDD and comorbid obesity appears urgently warranted.

5. Study objectives

5.1 Primary objective

To examine whether add-on 40 mg/d Simvastatin to standard antidepressant medication (Escitalopram 20 mg/d) improves depression to a greater extent than adjunct placebo in patients with major depression and comorbid obesity

5.1.1 Primary efficacy (confirmatory) endpoint

As primary outcome variable we will use the change in Montgomery-Åsberg Depression Rating Scale (MADRS) [58] score from baseline to week 12.

5.2 Secondary objectives

To examine whether add-on 40 mg/d Simvastatin to standard antidepressant medication (Escitalopram 20 mg/d) improves response rates, remission rates, patients' impression of change, quality of life, social functioning, self-report depression, lipid values, and immunometabolism / mitochondrial function to a greater extent than adjunct placebo in patients with major depression and comorbid obesity:

5.2.1 Secondary efficacy (exploratory) endpoints

As secondary outcome variables we will use

- MADRS response (defined as 50% MADRS score reduction from baseline),
- MADRS remission (defined as MADRS score < 10), and
- MADRS minimal clinically important difference (MCID).

The MCID concept originated from studies of patient-reported outcomes, in which it represented the smallest improvement that was considered meaningful by the patient [59]. Empirically, the MCID using the MADRS has been identified as a change from baseline between 1.6 – 1.9 [60].

Also, we will assess as secondary outcome variables

- change scores in patients' self-reported Beck Depression Inventory (BDI-II) as well as
- the MCID according to BDI [61]

5.2.2 Other secondary (exploratory) endpoints

We will additionally determine

- The clinician's impression of the severity of illness (Clinical Global Impression scale – Severity of illness, CGI-S) [62],
- the clinician's impression whether and to what extent symptoms have improved (Clinical Global Impression scale – Improvement, CGI-I) [62],
- the patient's impression whether and to what extent symptoms have improved (Patients' Global Impression of Change Scale, PGIC),
- social functioning (Social and Occupational Functioning Assessment Scale, SOFAS),
- quality of life (EuroQol-5 Dimensions-3 Levels Questionnaire, with a calculated MCID [63]),
- change scores in high-density lipoprotein (HDL), low-density lipoprotein (LDL), and total cholesterol,
- and change scores in mitochondrial function (spare respiratory capacity) of immune cells and indicators of immune function.

Please see Table 1 for characteristics and definitions of primary and secondary outcome variables.

Table 1: *Characteristics and definitions of primary and secondary endpoints.*

Outcome	Instrument	Rating	Domain	Exactly defined outcome	Variable
Primary	MADRS	Clinician-rated	Severity of Depression	Mean Change from Baseline	Continuous

Secondary / Efficacy (exploratory)	MADRS	Clinician-rated	Severity of Depression	- Percentage response (>50 % reduction from baseline)<br>- Percentage remission (MADRS score < 10 post-treatment)<br>- Percentage minimal clinically important difference<br>(MCID; change from baseline score > 1.9)	Dichotomous
Secondary / Efficacy (exploratory)	BDI-II	Self-rated	Severity of Depression	Mean Change from Baseline<br>- Percentage MCID (> 17.5% change from baseline)	Continuous<br>Dichotomous
Secondary Exploratory	CGI-S	Clinician-rated	Severity of illness	Median Change from Baseline	Ordinal
Secondary Exploratory	CGI-I	Clinician-rated	Improvement/worsening of illness	Response defined as “much improved” or “very much improved” post-treatment	Dichotomous
Secondary Exploratory	PGIC	Self-rated	Subjective Improvement	Response defined as “much improved” or “very much improved” post-treatment	Dichotomous
Secondary Exploratory	SOFAS	Clinician-rated	Social Functioning	Mean Change from Baseline	Continuous
Secondary Exploratory	EQ-5D-3L	Self-rated	Quality of Life	Mean Change from Baseline<br>Percentage MCID (change from baseline score > 0.74)	Continuous<br>Dichotomous
Secondary Exploratory	HDL, LDL, total cholesterol	Laboratory values	Metabolism	Mean Change from Baseline	Continuous
Secondary Exploratory	Mitochondrial and cellular function of immune cells	Laboratory values	Immune function	Mean Change from Baseline	Continuous

The time point for the evaluation of the secondary endpoints is 12 weeks. The secondary efficacy endpoints MADRS-response and MADRS-remission within 12 weeks will be analyzed by logistic regression models with intervention and center as factors and baseline MADRS as covariate. The analyses of continuous secondary efficacy endpoint BDI-II, and continuous secondary exploratory endpoints SOFAS, and EQ-5D-3L will follow the same lines as the primary outcome. The analyses of safety parameters and all secondary endpoints will have an exploratory character.

6. Study population

6.1 Number of patients

The study will include 160 patients (8 centers located in Germany; approximately 20 to 25 patients per center) divided into 2 groups:

- Group 1 (80 patients): Placebo add-on standard-medication
- Group 2 (80 patients): IMP add-on standard-medication

6.2 Selection of patients

We will include in- and outpatients with a major depressive episode according to DSM 5 (Diagnostic and Statistical Manual of Mental Disorders 5th Edition) and comorbid obesity (body mass index ≥ 30 as measured by weight and height). Patients will be recruited from the depression outpatient clinics and inpatient wards from each study site. In addition, we will use the existing and established networks of all sites within the psychiatric community to include depressed outpatients from general practitioners and psychiatrists. Thus, we will ensure high generalizability of our study population. There will be no specific gender distribution as no gender specific differences concerning efficacy and safety of the IMP are expected.

6.2.1 Inclusion criteria

- Written informed consent is present
- The patient has the capacity to give consent (He/she is able to understand the nature and anticipated effects/side effects of the proposed medical intervention)
- The patient has a major depressive episode according to DSM 5 (Diagnostic and Statistical Manual of Mental Disorders 5th Edition)
- The patient has a score of ≥ 18 in the Montgomery-Asberg Depression Rating Scale (MADRS)
- The patient has a body mass index ≥ 30
- The patient's age is between 18 and 65 years (≥ 18 und ≤ 65)
- The patient has not given childbirth within the 6 months prior to study entry and is not breastfeeding
- In case of non-psychotropic medication:
  The patient received stable pharmacological medication for at least 14 days prior to study entry (any changes in medication dose or frequency of therapy must be answered with no)
- The patient did not take antidepressants during the last 7 days prior to study entry (discontinuation of effective medication to enable study participation is prohibited)
- The patient did not receive prior treatment with Escitalopram in index episode
- The patient had less than three (<3) trials with antidepressants in index episode
- The patient does not have a history of non-response to Escitalopram
- The patient did not receive treatment with ketamine, electroconvulsive therapy (ECT) or other stimulatory treatments in index episode
- The patient does not meet any of the following criteria:
  - schizophrenia
  - schizoaffective disorder
  - bipolar disorder
- The patient is not diagnosed with dementia and does not have moderate or severe impairment of general cognitive function according to clinical impression
- The patient does not have clinically relevant elevated liver enzymes [GOT or GPT > 3 x upper limit normal (ULN)] and does not have elevated Carbohydrate Deficient Transferrin (CDT) ≥ 2.4 %
- The patient does not meet the criteria for alcohol use disorder (DSM-5: 303.90; ICD-10: F10.20) or substance use disorder (DSM-5: 304; ICD-10: F11.20 – F19.20) in M.I.N.I. for DSM-5 and a urine/serum drug screening is negative (except for benzodiazepines and opiates)
- The patient does not have a history of suicide attempt
- The patient does not have diagnosed epilepsy or increased bleeding diathesis or a history of angle closure glaucoma or other glaucomas

- The patient did not have bariatric surgery prior to study entry
- The patient does not have a known allergy or contraindication against Escitalopram or Simvastatin
- The patient does not meet any of the following criteria:
  - hereditary muscle disease
  - known history of rhabdomyolysis
  - elevated creatine kinase (CK) outside of the sex-specific reference intervals
  - History of muscular symptoms under treatment with statins or fibrates
- The patient does not have elevated TSH level outside of the age- and sex-specific reference intervals.
- The patient does not have insulin-dependent diabetes mellitus
- The patient does not have uncontrolled hepatic disorder, renal or cardiovascular disease
- The patient does not have untreated hypothyroidism
- The patient does not have a history of myocardial infarction or stroke
- The patient does not have symptomatic peripheral arterial disease
- The patient does not have monogenic familial hypercholesterolemia
- The patient does not have clinically significant laboratory abnormalities
- The patient did not participate in other interventional trials during the 6 months before and at the time of this trial
- The patient is not an employee of the investigator study site, or a family member of the employees or the investigator, or otherwise dependent on the sponsor, the investigator or the investigator study site

Please note that having type 2 diabetes will not offend inclusion criteria (except if insulin-dependent) because large beneficial effects of Simvastatin 40 mg/d have been shown on cardiovascular endpoints in these patients [64].

6.2.2 Exclusion criteria

- The patient has current use of statins (for visits 2-6 applies: except for IMP Simvastatin)
- The patient has current use of antidepressants (for visits 2-6 applies: except for standard medication Escitalopram)
- The patient has acute suicidal tendencies (MADRS Item 10 > 4)
- The patient uses potent CYP3A4-inhibitors (e.g. clarithromycin, erythromycin, HIV protease inhibitors – see “Risks, adverse drug reactions, drug interactions, restrictions, contraindications, procedures in case of emergency”)
- The patient uses potent CYP3A4 inductors (carbamazepine, efavirenz, nevirapin, etravirine).
- The patient uses Fibrates, Amiodaron, Amlodipin, Verapamil, Fluconazol, Diltiazem, Fusidic acid, Niacin, Daptomycin or Lomitapid or BCRP-Inhibtors (e.g. Elbasvir or Grazoprevir)
- The patient uses Gemfibrozil, Ciclosporin or Danazol
- The patient has known hypersensitivity to other ingredients of Simvastatin and Escitalopram [butylated hydroxyanisole, microcrystalline celluose, citric acid, starch, lactose, magnesium stearate, hypromellose, talc, titanium dioxide, iron oxies, colloidal silicon dioxide, croscarmellose sodium, polyethylene glycol]
- The patient uses medication that is associated with QTc-prolongation [antiarrhythmica class IA and III, antipsychotics (e.g. haloperidol), phenothiazines, tricyclic antidepressants, antibiotics (e.g. moxifloxacin), and certain antihistaminergic drugs (e.g. astemizol, mizolastin)]
- The patient has clinically significant abnormalities in 12-lead ECG (e.g. QTc-prolongation

≥ 500 ms or increase ≥ 60 ms from baseline visit)

- The patient is pregnant
- The patient with childbearing potential is not willing to use an acceptable form of contraception (defined as Pearl index < 1)
- The patient has current use of psychotropic medication (e.g. antipsychotics, anticonvulsants, lithium or St. John's Wort) except for benzodiazepines, non-benzodiazepines and opiates
- The patient uses nonselective, irreversible monoamine oxidase (MAO) inhibitor (e.g. Tranylcypromine) or selective, reversible inhibitor of monoamine oxidase A (e.g. Moclobemide) or the nonselective, reversible monoamine oxidase inhibitor Linezolid
- The patient is unwilling to consent to saving, processing and propagation of pseudonymized medical data for study reasons
- The patient is legally detained in an official institution
- The patient has active SARS-CoV-2 infection

6.2.3 Contraception

Highly effective contraception in women (defined as pearl index < 1), or complete abstinence of intercourse (during study participation).

Female patients who are not either surgically sterile (tubal ligation/obstruction or removal of ovaries or uterus) or post-menopausal (no spontaneous menstrual periods for at least one year confirmed by a negative hormone panel) must commit to using one highly effective method (such as intrauterine device, sterilisation of one of the partners, hormonal birth control methods) for the duration of the study.

Birth control methods which are considered as highly effective (low failure rate less than 1% per year) when used consistently and correctly include:

- Established use of oral, injected or implanted hormonal methods of contraception.
- Placement of an intrauterine device or intrauterine system.
- Female bilateral tubal ligation.
- Sexual abstinence: When this is in line with the preferred and usual lifestyle of the patient.

Note: Periodic abstinence (e.g. calendar ovulation, symptothermal, post-ovulation methods) and withdrawal are not highly effective methods of contraception

Pregnancy testing: For women of childbearing potential, a highly sensitive serum HCG test will be conducted at screening visit, baseline, week 4, week 8 and at the end-of-study.

6.2.4 Follow up of pregnancy

A patient must immediately inform the investigator if she becomes pregnant during the study. The investigator will report all pregnancies to the Sponsor/Sponsor representative on the form provided within 24 h after knowledge. Every pregnant female will be excluded from the trial immediately and referred to outpatient obstetrics and gynecology for further medical care. Following local regulation monitoring of the subject will continue until the conclusion of the pregnancy and until 3 months after the birth of the baby. For further information on premature termination of the individual participant see 11.1.

6.2.5 Methods of avoiding simultaneous enrolment in other trials

Patients who participated in other interventional trials during the 6 months before and at the time of this trial will not be included in the study. It is the duty of the recruiting investigator to rule out

current participation in another trial. Patient information will explicitly state the impossibility to partake if already participating in another trial.

6.2.6 Screening failures

Patients who sign an informed consent form but fail to meet the eligibility criteria are defined as screening failures. For all screening failures, the investigator is asked to maintain a screening log that documents the patient number, reason(s) for screening failure and justification for rescreening if applicable. A copy of this log should be retained in the investigator site file. The end of study form and other forms containing information needed to examine the inclusion- and exclusion criteria (e.g. demography, MADRS scale) leading to screening failure will be completed and documented using eCRF – secuTrial at each study site. In case of screening failures further treatment will be offered in specialized outpatient clinics for depression and obesity. If screening failures occur due to medical reasons patients will be referred to specialized out- or inpatient clinics for the treatment of this medical condition.

7. Plan for medical treatment

7.1 Study design

We will conduct a randomized, placebo-controlled, double-blind multicenter trial with parallel-group design (Phase II). Subjects will be randomized 1:1 to IMP or placebo based on separate randomization lists for each study site provided by the biostatistician. The randomization will be based on a permuted block procedure stratified by center.

Subinvestigators and patients will be blinded to the treatment. Simvastatin is an effective serum lipid-lowering drug that can decrease lipid levels. In order to ensure continued blinding, laboratory assessment will be divided into safety laboratory, laboratory (lipid assessment) and laboratory (immunometabolism/mitochondrial function). Laboratory (lipid assessment) will be conducted only at V1 and V6 to minimize the risk for unblinding of investigators/subinvestigators by revealing the lipid profile. Particularly LDL-cholesterol is expected to be lower in the treatment group. At the same time, not assessing the lipid status of patients during the entire study participation would not represent current practice where lipid status is an important part of cardiovascular risk assessment. No unblinding of the subinvestigators due to specific side effects of Simvastatin treatment is to be expected (see 9.5).

7.2 Description of study medication

Each patient in this study will be treated with Escitalopram - a well-established standard-antidepressant, which is readily available because it is commonly used as generic drug in Germany. Adjunct placebo serves as the control condition for adjunct Simvastatin (40 mg/d fixed dose). Simvastatin and placebo will be provided by the pharmacy of the Charité and will be administered in a double-blind fashion. Patients will receive IMP or placebo at baseline (visit 1), at visit 4 and visit 5. Patients will receive 10 mg Escitalopram at baselie (visit 1) and then 20 mg Escitalopram at visit 3, visit 4, and visit 5 (see 7.2.4 for treatment schedule and instructions for dose adjustments). Please see **Figure 1** for scheme of intervention.

7.2.1 Investigational Medical product (IMP)

Among the available stains, we decided to use Simvastatin (SimvaHEXAL®) in a dosage of 40 mg/d based on the following considerations: Simvastatin was used in this dose in one of three RCTs showing evidence vs. placebo as adjunct treatment to SSRI [36]; it was superior to atorvastatin in depressed patients with heart disease [38]; it passes the blood-brain barrier [40], and it is

available as generic drug (costs for 40 mg/d Simvastatin = 0.24 Euro/day = 87.6 Euro/year). We decided against using a dose of 80 mg/d Simvastatin due to the increased risk of muscle injury and the corresponding warning from the US Food and Drug Administration [65]. See for further information on risk-benefit assessment 10.

7.2.2 Standard medication

Escitalopram will be provided by the pharmacy of the Charité, stored and dispensed at each study site in a non-blinded fashion.

Current treatment with antidepressants is an exclusion criterion. Study participation may be permitted in case of wash-out of prior antidepressants for at least 7 drug-free days prior to inclusion. This will be permitted only if the depression severity had not sufficiently improved under the prior antidepressant regime as shown by a MADRS score ≥ 18 at screening visit. Discontinuation of effective medication to enable study participation is prohibited.

7.2.3 Control (Placebo)

IMP and placebo indistinguishable in form, color, and taste will be provided by the pharmacy of the Charité (see 7.2.8) and will be administered in a double-blind fashion.

In order to measure blinding, we will ask patients which treatment they believe they have been assigned at the end of study.

7.2.4 Treatment schedule / Dose regimen

Instructions for application:
IMP - Simvastatin: The majority of cholesterol synthesis appears to occur at night, presumably reflecting the effects of a fasting state. For this reason, it is typically recommended that the statins with shorter half-lives including Simvastatin be administered in the evening or at bedtime. Patients should not drink grapefruit juice during Simvastatin treatment. IMP will be administered orally in a fixed dosage of 40 mg once a day at bedtime. IMP can be taken with or without food, but patients are recommended to take it with a glass of water.

Control - Placebo: Placebo will be administered orally once a day at bedtime. Placebo can be taken with or without food, but patients are recommended to take it with a glass of water.

Standard medication - Escitalopram: Escitalopram will be administered orally in a fixed dosage of 10 mg once a day in the morning for the first two weeks, then increased to 20 mg once a day in the morning until the end of study. Escitalopram can be taken with or without food, but patients are recommended to take it with a glass of water.

Table 2 *treatment schedule*

	**morning**	**lunchtime**	**evening**	**night**
**Simvastatin, orally**	0	0	0	40 mg
**Placebo**	0	0	0	1
**Escitalopram, orally**	20 mg (10 mg for first two weeks)	0	0	0

Instructions for dose adjustment:
Simvastatin (IMP): fixed dose 40 mg/d during 12 weeks without dose-adjustment

Placebo: not applicable

Escitalopram (standard antidepressant that each patient will receive):

- 10 mg/d for first two weeks, then increase to 20 mg/d until end of study
- If side effects occur in the first two weeks, 10 mg/d Escitalopram can be reduced to 5 mg/d until the next visit, then ideally increased to 10 mg/d again unless side effects persists.
- If side effects occur after the increase to 20 mg/d, Escitalopram can be reduced to 10 mg/d until next visit, then ideally increased to 20 mg/d again unless side effects persists.

7.2.5 Adverse drug reactions and restrictions, contraindications, drug interactions

In randomized trials, statin therapy appears to cause only a slight increased risk of side effects compared with placebo, and no increased risk of discontinuation of therapy compared with placebo [46]. In 2017, a meta-analysis of 22 placebo-controlled trials on statin use involving nearly 130,000 participants showed that 13.3 % of subjects receiving a statin discontinued the drug compared with 13.9 % of subjects on placebo (odds ratio [OR] 0.99, 95% CI 0.93-1.06) over a mean follow-up of 4.1 years [47].

Simvastatin is the IMP that will be tested versus placebo as add-on to standard antidepressant medication with Escitalopram, which will be given open label to each patient. In the following, we list the risks and potential adverse drug effects for both medications.

Simvastatin

Simvastatin is approved for several indications since decades and has in general proven to be a safe medication. The risks and adverse effects listed below are all taken from the latest version of German “Fachinformation”.

The most prominent risk of statin use is myopathy (rarely), associated with muscle pain, muscle tenderness, muscle weakness and increases of creatine kinase (CK). The most severe form of myopathy is rhabdomyolysis with or without acute renal failure secondary to myoglobinuria that occurs very rarely and is dose-dependent. The combined incidence for myopathy/rhabdomyolysis in 41.413 patients treated for at least 4 years was 0.03% for 20 mg/d, 0.08% for 40 mg/d, and 0.61% for 80 mg/d Simvastatin. Thus, to minimize the risk of Simvastatin, we will use it in a medium dose (40 mg/d) and not a high dose (80 mg/d). In addition to safety laboratory values at screening, baseline, week 4, and week 12, it will be explicitly asked if the patient experienced unexplained muscle pain or dark urine as a sign of rhabdomyolysis.

Other adverse effects are listed below, taken from the latest version of German “Fachinformation” and classified as follows:
Very often: (>1/10); often (> 1/100); sometimes (> 1/1000), rarely (> 1/10.000), very rarely (< 1/10.000); not known *(cannot be determined on basis of available data)*

Very often: none
Often: none
Sometimes: none
Rarely: Anemia, headache, dizziness, paresthesia, peripheral neuropathy, obstipation, stomach ache, flatulence, dyspepsia, diarrhea, nausea, emesis, pancreatitis, hepatitis/icterus, rash, pruritus, alopecia, myopathy, rhabdomyolysis, myalgia, muscle cramps, asthenia, vision blurred, visual impairment
Very rarely: insomnia, memory complaints, liver failure, lichenoid drug eruptions, muscle rupture, gynecomastia
Not known: depression, interstitial lung disease, tendinopathy, immune-mediated myopathy, erectile dysfunction

Medication that is contraindicated with Simvastatin due to inhibition of CYP3A4 will not be permitted (Itraconazol, Ketoconazol, Posaconazol, Voriconazol, Erythromycin, Clarithromycin, Telithromycin, HIV protease inhibitors (e.g. Nelfinavir), Boceprevir, Telaprevir, Nefazodon, Cobicistat, Ciclosporin, Danazol, Gemfibrozil).

Further, medication that is not recommended to use in parallel to Simvastatin in a dosage of 40 mg/d will not be permitted (Fibrates, Amiodaron, Amlodipin, Verapamil, Fluconazol, Diltiazem, fusidic acid, Niacin, Daptomycin, Lomitapid).

Ticagrelor is not recommended to use in parallel to Simvastatin in a dosage greater than 40 mg/d. Since in this study Simvastatin is given in a dosage of 40 mg/d, Ticagrelor will be permitted.

Finally, patients will be noticed that they should not drink grapefruit juice during Simvastatin treatment due to inhibition of CYP3A4 and corresponding increases in Simvastatin plasma levels.

In turn, medication that induces CYP3A4 leading to decreased Simvastatin levels will also not be permitted (Carbamazepine, Efavirenz, Nevirapin, Etravirine).

Patients with contraindications for Simvastatin will not be included or excluded from the study in case of later occurrence (see under inclusion criteria).

Escitalopram

The most prominent risk of Escitalopram is QTc-Prolongation in the ECG that can lead to ventricular arrhythmia (torsade des pointes). To exclude patients with QTc-prolongation an ECG will be conducted at screening. Furthermore, an additional safety ECG will be conducted at every visit.

Other adverse effects are listed below, taken from the latest version of German “Fachinformation” and classified as follows:
Very often: (>1/10); often (> 1/100); sometimes (> 1/1000), rarely (> 1/10.000), very rarely (< 1/10.000); not known (cannot be determined on basis of available data)

Very often: headache, nausea
Often: decreased appetite, increased appetite, weight gain, anxiety, restlessness, nightmares, decreased libido, anorgasmia, insomnia, fatigue, dizziness, paresthesia, tremor, sinusitis, yawning, diarrhea, obstipation, emesis, dry mouth, hyperhidrosis, arthralgia, myalgia, delayed ejaculation, erectile dysfunction, fever
Sometimes: weight loss, bruxism, agitation, nervousness, panic attacks, confusion, disturbed taste, insomnia, syncope, mydriasis, blurred vision, tinnitus, tachycardia, epistaxis, gastrointestinal bleeding, urticarial, alopecia, rash, pruritus, metrorrhagia, menorrhagia, edema
Rarely: anaphylaxis, aggression, depersonalization, hallucination, serotonin syndrome, bradycardia
Very rarely: none
Not known: thrombocytopenia, inadequate ADH-secretion, hyponatremia, anorexia, mania, suicidality, dyskinesia, seizures, akathisia (psychomotor restlessness), QTc-prolongation in the ECG, orthostasis, hepatitis, increased liver enzymes, ecchymosis, angioedema, urinary retention, galactorrhea, priapism

Medication that is contraindicated with Escitalopram will not be permitted, which includes all irreversible MAO-inhibitors and medication that is associated with QTc-prolongation [antiarrhythmica class IA and III, antipsychotics (e.g. haloperidol), phenothiazines, tricyclic antidepressants, antibiotics (e.g. moxifloxacin), and certain antihistaminergic drugs (e.g. astemizol, mizolastin)].

Patients with contraindications for Escitalopram will not be included (please see under inclusion criteria).

7.2.6 Procedures in case of emergency

In case of emergency supposedly due to application of the medication, they will be immediately discontinued. The blinding will be broken immediately, and the patient will receive immediate medical care (see 7.2.9). No specific antidote to Simvastatin or Escitalopram are currently known, therefore treatment is limited to symptom management. Regulatory reporting duties apply.

7.2.7 Randomization

We will conduct a randomized, placebo-controlled, double blind multicenter study (RCT). Subjects will be randomized 1:1 to Simvastatin or placebo based on separate randomization lists for each study site provided by the biostatistician. The randomization will be based on a permuted block procedure stratified by center. We will additionally collect data on prognostic variables (e.g. chronicity of depression, number of prior treatment attempts, etc.) as recommended by EMA [66] but will not stratify for these variables because a) there are a several predictive variables and it is not feasible to stratify randomization for all of them and b) we expect that these predictive variables will not systematically differ between the two treatment groups because of randomization. The Charité pharmacy will be instructed in proper allocation concealment. Based on the randomization codes, the pharmacy will centrally provide for each center sequentially numbered, tamper-proof containers, which are equal in weight and similar in appearance containing IMP or placebo. The pharmacy keeps the randomization lists unavailable to investigators and the Sponsor/Sponsor representative until the end of study.

7.2.8 Packaging, labelling, and blinding procedures

Charité Pharmacy encapsulates and packages IMP and placebo in identical vials with a medication ID. The IMP/placebo vials will be provided by the Charité pharmacy to study sites. Medication is prepared and concealed by Charité Pharmacy. Concealment will not be broken until completion of the study, therefore double-blinding remains intact.

Emergency envelopes for the unblinding are enclosed in the packages sent to the investigator site and are stored by the local investigator. Envelopes are only to be opened in case of emergency and if treatment decisions depend on knowledge of application of IMP. Patient information contains contact information to ensure possibility of unblinding after treatment if necessary.

7.2.9 Unblinding

In case of an emergency the blinding will be broken immediately, and the patient will receive immediate medical care.

Premature Unblinding
Premature unblinding happens at the discretion of the local investigator in case of a medical emergency, where treatment is based on the knowledge whether IMP has been applied. When emergency envelopes are opened prematurely, date, time, reason for opening and name of the person opening have to document in source data and CRF. After unblinding the patient has to be immediately excluded from the study.

An identical copy of the emergency envelopes for the unblinding will be sent to the Sponsor representative and stored securely at the coordinating site (Charité - Universitätsmedizin Berlin). This way the coordinating staff is additionally available for immediate unblinding in case of a medical emergency, where treatment is based on the knowledge whether IMP has been applied. The same rules apply as for the premature unblinding by the local investigator.

Regular Unblinding
After completion of the study and data base closure, Charité pharmacy is requested by the Sponsor/Sponsor representative to send the randomization lists and all participants will be unblinded.

Furthermore, the Data Monitoring Committee (DMC) is granted access to limited randomization lists of the subjects they are currently reviewing.

7.2.10 Storage requirements and administration (shipping and destruction)

Study medication will be delivered to the study sites by the Charité pharmacy for each individual participant after successful screening visit. Receipt will be confirmed via an acknowledgement of receipt dated and signed by site study staff, which will be stored in the investigator site file (ISF). At the investigator sites, IMP/placebo must be kept separate from regular medication in a lockable cupboard with access only to study staff.

Upon distribution/application of study medication (IMP/placebo, and standard medication) to the patient, exact amount, date, medication ID and randomization number are documented in the ISF (drug account). It is the investigator's obligation to assure correct documentation. The same procedure applies if medication is returned. The monitor will regularly check the drug account logs. Only after this check unused medication (IMP/placebo) will be returned to Charité pharmacy and destroyed. Charité pharmacy documents retrieval and destruction of IMP not applied. This documentation will be filed in the TMF.

7.2.11 Treatment compliance

Patients will receive study medication for four weeks at baseline and then at week 4, and at week 8. Standard medication will be distributed for two weeks at baseline and then at week 2, at week 4, and at week 8. Patients are instructed to bring back the medication package and compliance will be determined by count of the remaining pills and patients will be considered compliant if they report taking more than 90 % of their drug (as in previous studies with Simvastatin in central nervous system diseases [7, 67]).

8. Concomitant medication / concomitant therapy

Medication that is contraindicated with Simvastatin, and Escitalopram will not be permitted (see 7.2.5).

- Use of potent CYP3A4-inhibitors (e.g. clarithromycin, erythromycin, HIV protease inhibitors).
- Use of potent CYP3A4 inductors (carbamazepine, efavirenz, nevirapin, etravirine).
- Medication that is associated with QTc-prolongation [antiarrhythmica class IA and III, antipsychotics (e.g. haloperidol), phenothiazines, tricyclic antidepressants, antibiotics (e.g. moxifloxacin), and certain antihistaminergic drugs (e.g. astemizol, mizolastin)].
- Use of nonselective, irreversible monoamine oxidase inhibitor (e.g. Tranylcypromine) or selective, reversible inhibitor of monoamine oxidase A (e.g. Moclobemide) or the nonselective, reversible monoamine oxidase inhibitor Linezolid.

Concomitant treatment with antidepressants other than Escitalopram will not be permitted. In case of current treatment with antidepressants at study entry a wash-out of prior antidepressants for at least 7 drug-free days prior to inclusion may be permitted. However, discontinuation of effective medication to enable study participation is prohibited.

Other psychotropic drugs (including antipsychotics, anticonvulsants, lithium, or St. John's Wort) are not permitted during the study period with the following exceptions.

- In case of sleep difficulties, benzodiazepine or nonbenzodiazepine hypnotics (e.g. zolpidem, zopiclone) will be allowed on an "as needed basis".
- In case of acute anxiety or agitation, a benzodiazepine (preferably lorazepam) may be prescribed not exceeding 3 mg/d (other benzodiazepines at the equivalent dosage).
- Opiates for pain relief.

Any clinical exacerbation requiring a higher benzodiazepine regimen or prescription of antipsychotics (“rescue medication”) will be considered as a serious adverse event.

In case of non-psychotropic medication stable pharmacological medication for at least 14 days prior to study entry will be permitted. Patients with any changes in medication dose or frequency of therapy during the 14 days prior to study entry will not participate in the study (see 6.2.1). Concomitant use of statins will not be permitted.

9. Study procedures

To ensure that the study is conducted according to the study protocol, to current laws and to guidelines, a training of all investigators/subinvestigators will be conducted prior to initiation. The trial will be initiated by a monitor.

The subinvestigator will perform the following evaluations: informed consent and medical history, treatment status, inclusion/exclusion criteria, concomitant medication, physical exam, ECG interpretation, assessment of blood pressure and heart rate, adverse events recording, all efficacy assessments (see 9.5), all safety assessments (see 9.6), drug dispensation, laboratory assessment (lipid and immuno-metabolism/mitochondrial function), and other procedures (see 9.7.3).

For all patients without proof of complete vaccination upon arriving at study center, a SARS-CoV-2 Rapid Antigen Test will be performed by study staff.

9.1 Recruitment / Screening procedures

Based on the power calculation described in chapter 16, we aim to recruit a total of 160 patients at several study sites within Germany. The time allotted for recruitment is 39 months. All centers intended for participation are specialized tertiary depression clinics, where large numbers of depressed in- and outpatients are routinely seen in both clinical care and research contexts. Patients will be recruited from the depression outpatient clinics and inpatient wards from each study site. In addition, we will use the existing and established networks of all sites within the psychiatric community to include depressed outpatients from general practitioners and psychiatrists. All contacted investigators have extensive experience with screening, recruiting, and assessing patients with MDD for clinical trials. Recruitment estimations are based on current reviews of the hospital records of patients with MDD at each of these institutions. Before initiation of the trial, recruitment capabilities will again be assessed during scheduled pre-trial visits to all sites, after which a final decision for inclusion of a given site in the trial will be made.

9.2 Informed consent

Patients will be informed about the conduction of the trial in a personal conversation by the investigator/subinvestigator. They will receive the written patient information (“Information zur Studie”) and be given time to read it thoroughly and without haste. If further questions arise, the investigator/subinvestigator will answer them openly and correctly. The patients’ consent to participate in the study will be documented by signature and dating of the consent form (“Einwilligungserklärung”) which will be countersigned by the investigator/subinvestigator.

9.3 Patient identification / Generation of pseudonym

Patient related data will be recorded in study files (source data) under the participant`s real name. The information required by the protocol will be entered onto the electronic case report forms (eCRF - secuTrail) under a pseudonym. Pseudonyms will be generated by secuTrail and will be comprised of a combination of numbered and lettered sequence without the use of initials and date of birth. Every patient will receive a patient number / pseudonym which will be unique for

this individual patient. The investigator will compile a confidential list (patient identification log), which relates these patient numbers to the patient's full name. This list will only be accessible to the study staff and the monitor. The investigator must print the patient identification log and store it with other study documents, e.g. the protocol, and any protocol amendments, in a secure place. The databank will contain only pseudonymized data. Re-identification of patients will only be possible through the confidential list.

9.4 Visits schedule and evaluations

***Table 3**: Overview and frequencies of trial examinations*

Assessments	Screen-ing	Base-line (Visit 1)	Visit 2	Visit 3	Visit 4	Visit 5	End-of-study (Visit 6)
		week 0	week 1	week 2	week 4	week 8	week 12
Screening and consent							
Informed consent	X						
Inclusion criteria		X					
Exclusion criteria		X	X	X	X	X	X
Medical history	X						
Treatment status		X					
M.I.N.I – DSM 5	X						
Urine/serum drug screening	X						
Concomitant medication	X						
Safety							
SARS-CoV-2 Rapid Antigen Test (for all patients without proof of complete vaccination)							
Physical exam	X						X
ECG	X	X	X	X	X	X	X
Blood pressure & heart rate	X	X	X	X	X	X	X
Safety laboratory (incl. Pregnancy test - women only)	X	X			X	X	X
Adverse events recording			X	X	X	X	X
Effectiveness							
MADRS	X	X	X	X	X	X	X
BDI-II		X	X	X	X	X	X
Social functioning and quality of life							
SOFAS		X			X		X
EQ-5D-3L		X			X		X
Other							
Demographics	X						
BMI	X	X					X
Waist circumference		X					X
PGIC			X	X	X	X	X
CGI-S		X	X	X	X	X	X

CGI-I		X	X	X	X	X
Laboratory (lipid assessment)	X					X
Laboratory (immunometabolism / mitochondrial function)	X			X	X	X
Drug dispensation	X		X	X	X	

Note: Electrocardiography (ECG), Body-Mass-Index (BMI), Mini International Neuropsychiatric Interview (MINI), Montgomery-Asberg Depression Scale (MADRS), Beck-Depressions-Inventar (BDI), Patient Global Impression of Change (PGIC), Clinicians' Global Impression of Severity of illness (CGI-S), Clinicians' Global Impression of Improvement (CGI-I), Social and Occupational Functioning Assessment Scale (SOFAS), Generic Quality of Life Questionnaire (EQ-5D-3L).

Maximum variation of ± 3 days is permitted for the scheduling of each study visit.

9.4.1 Screening visit

After informed consent, we will assess the severity of depressive episode, duration of the depression and of the index episode, number of episodes, previous treatment as requested by the EMA guideline on clinical trials in depression [66].

During screening the following procedures will be performed:

- Medical history, concomitant medication, and physical examination
- M.I.N.I for DSM-5
- Body weight, height (for determination of body mass index)
- Blood pressure, heart rate
- ECG
- Laboratory (safety)
- Pregnancy test (women only)
- Urine/serum drug screening
- Demographics
- MADRS

9.4.2 Randomization visit (Baseline) - Drug dispensation

Patients will be examined for inclusion and exclusion criteria in the randomization visit. Patients fulfilling all inclusion criteria and no exclusion criterion based on information assessed at the screening visit will be randomized in the two treatment groups (IMP vs. placebo). Randomization is carried out by the biostatistician. Patients will not be explicitly asked to pursuit weight loss during the study participation. We will assess the treatment status of the patient (inpatient/daycare/outpatient).

During Baseline (**visit 1 – week 0**) the following procedures will be performed:

- Assessment of in- and exclusion criteria
- Blood pressure, heart rate
- ECG
- Laboratory (safety)
- Pregnancy test (women only)
- MADRS
- BDI-II
- SOFAS, EQ-5D-3L
- CGI-S
- Determination of body mass index and measurement of waist circumference
- Laboratory (lipid assessment)
- Laboratory (immunometabolism / mitochondrial function)

Furthermore, patients will receive **IMP or placebo** for four weeks and **Escitalopram** 10 mg/d for two weeks.

9.4.3 Examinations during trial

Visit 2 (week 1)
During **visit 2 – week 1** the following procedures will be performed:
The exclusion criteria will be reassessed.
Safety examinations during the trial include:

- Blood pressure, heart rate
- ECG
- Adverse events recording

Efficacy examinations during the trial include:

- MADRS
- BDI-II

Other examinations during the trial include:

- PGIC
- CGI-S
- CGI-I

Visit 3 (week 2) - Drug dispensation
During **visit 3 – week 2** the following procedures will be performed:
The exclusion criteria will be reassessed.
Safety examinations during the trial include:

- Blood pressure, heart rate
- ECG
- Adverse events recording

Efficacy examinations during the trial include:

- MADRS
- BDI-II

Other examinations during the trial include:

- PGIC
- CGI-S
- CGI-I

Patients will receive **Escitalopram** 20 mg/d for two weeks.

Visit 4 (week 4) - Drug dispensation
During **visit 4 – week 4** the following procedures will be performed:
The exclusion criteria will be reassessed.
Safety examinations during the trial include:

- ECG
- Blood pressure, heart rate
- Laboratory (safety)
- Pregnancy test (women only)
- Adverse events recording

Efficacy examinations during the trial include:

- MADRS
- BDI-II

Other examinations during the trial include:

- SOFAS, EQ-5D-3L, and PGIC
- CGI-S
- CGI-I
- Laboratory (immunometabolism / mitochondrial function)

Patients will receive **IMP or placebo** and **Escitalopram** 20 mg/d for four weeks.

Visit 5 (week 8) - Drug dispensation
During **visit 5 – week 8** the following procedures will be performed:
The exclusion criteria will be reassessed.
Safety examinations during the trial include:

- Blood pressure, heart rate
- ECG
- Laboratory (safety)
- Pregnancy test (women only)
- Adverse events recording

Efficacy examinations during the trial include:

- MADRS
- BDI-II

Other examinations during the trial include:

- PGIC
- CGI-S
- CGI-I
- Laboratory (immunometabolism / mitochondrial function)

Patients will receive **IMP or placebo** and **Escitalopram** 20 mg/d for four weeks.

9.4.4 Final examination (End-of-study)

During Final examination (**visit 6 – week 12**) the following procedures will be performed:
The exclusion criteria will be reassessed.
Safety examinations during the trial include:

- Physical examination
- ECG
- Blood pressure, and heart rate
- Laboratory (safety)
- Pregnancy test (women only)
- Adverse events recording

Efficacy examinations during the trial include:

- MADRS
- BDI-II

Other examinations during the trial include:

- SOFAS, EQ-5D-3L, and PGIC
- CGI-S
- CGI-I
- Determination of body mass index and measurement of waist circumference
- Laboratory (lipid assessment)
- Laboratory (immunometabolism / mitochondrial function)

9.5 Evaluations / Efficacy assessments

9.5.1 *MADRS*

As primary outcome variable we will use the change in Montgomery-Åsberg Depression Rating Scale (MADRS) score from baseline to week 12. All subinvestigators will be trained to use the MADRS according to the published structured interview guide to enhance inter-rater reliability [68].

The MADRS is a clinician rated interview to measure the severity of depression within the last seven days [58]. It consists of 10 items that can be ranked on a 7-point scale ranging from 0 to 6. This variable is explicitly recommended by the European Medicines Agency (EMA) as a primary outcome to determine efficacy in the treatment of depression: “Improvement should be documented as the difference between baseline and post-treatment score in signs and/or symptoms, but should also be expressed as the proportion of responders” (page 9 of EMA guideline on clinical investigation of medicinal products in the treatment of depression)[66]. The German version of the MADRS has been shown to be a valid instrument with high internal consistency and high sensitivity for change [69]. All subinvestigators will be trained to use this scale and interviews will be conducted according to the specific interview guide that has been developed for the MADRS [68]. MADRS will be conducted at every visit during the study by the subinvestigator.

9.5.2 *BDI-II*

The Beck Depression Inventory (BDI)-II is a well-established self-report questionnaire to measure depressive symptoms [70]. Patients will be asked to fill out the BDI-II at every visit except for screening.

9.6 Evaluations / Safety assessments

9.6.1 Clinical examination

Physical examination
The physical examination comprises a routine medical examination including neurological and cognitive assessments. The following body systems will be examined: HEENT (head, eyes, ear, nose, and throat), urological, cardiovascular, respiratory, lymphatic, gastrointestinal, musculoskeletal, dermatological, and venous system. The physical examination will take place at screening and at end-of-study by the subinvestigator.

Blood pressure, and heart rate (vital signs)
The following vital signs will be assessed at each visit by the subinvestigator:

- Systolic (SBP) and diastolic (DBP) blood pressure (mmHg)
- Heart rate (beats per minute [bmp])

Blood pressure (SBP and DBP) and heart rate will be measured using a semi-automatic blood pressure recording device with an appropriate cuff size. Supine blood pressure and heart rate will be measured after the patient has rested in a supine position for at least 5 minutes.

9.6.2 Laboratory safety

Safety laboratory will be performed at screening, baseline, visit 4, visit 5 and at the end-of-study. Components of the laboratory panels include tests for the following:

- Blood count (leukocytes, erythrocytes, hemoglobin, hematocrit, MCV, MCH, MCHC, RDW-CV, thrombocytes, MPV) and differential blood count (neutrophiles, granulocytes, lymphocytes, monocytes, eosinophiles, basophiles, neutrophiles)
- Clinical chemistry [sodium, potassium, creatinine, estimated GFR, GOT (AST), GPT (ALT), gamma-GT
- Creatine kinase (CK)
- CRP
- Basal glucose and HbA1c
- Beta-HCG (women only).

At screening visit thyroid-stimulating hormone (TSH) and Carbohydrate-deficient Transferrin (CDT) will be additionally determined.

Sample collection will take place at each investigator site by the subinvestigator. The sample is obtained by using a needle to draw off blood from veins into collection tubes and then immediately transported to the local laboratory. Blood samples will be stored until all analyses are completed. Safety laboratory results will be evaluated by the subinvestigator.

9.6.3 Electrocardiogram

12-lead ECG will be obtained at every visit. After the subject has rested in a supine position for at least 5 minutes, 12-lead ECG will be performed and interpreted by the subinvestigator. Measurements that deviate substantially from previous readings will be reported. The most prominent risk of Escitalopram is QTc-Prolongation in the ECG that can lead to ventricular arrhythmia (torsade des pointes). To exclude patients with QTc-prolongation an ECG will be conducted at every visit. If QTc reaches or exceeds 500 ms or increased ≥ 60 ms from baseline, subject will be immediately discontinued [71].

9.6.4 MINI International Neuropsychiatric Interview

MINI International Neuropsychiatric Interview (German for Germany Translation Version 7.0.2) will be performed at screening visit by the subinvestigator to assess psychiatric comorbidities and evaluate inclusion and exclusion criteria [72].

9.7 Evaluations / Other assessments

9.7.1 Patient reported outcome measures

PGIC
The Patient Global Impression of Change (PGI-C) measures the patient's subjective improvement [73]. Patients will be asked to fill out the PGIC at every visit except for screening and baseline.

EQ-5D-3L
Quality of life will be determined with the EuroQol-5 Dimensions-3 Levels Questionnaire [74]. Patients will be asked to fill out the EQ-5D-3L at baseline, week 4 and end-of-study.

9.7.2 Laboratory assessments

Lipid assessment
Sample collection will take place at each investigator site by the subinvestigator. The sample is obtained by using a needle to draw off blood from veins into collection tubes and then immediately transported to the local laboratory. Blood samples will be stored until all analyses are completed.

- High-density lipoprotein (HDL)
- Low-density lipoprotein (LDL)
- Total cholesterol

Immunometabolism / mitochondrial function
At selected sites in Hamburg and Berlin, additional blood samples (80 ml heparin blood samples and 5 ml serum sample) for assessments of immunometabolism / mitochondrial function will be analyzed within the internal research laboratories and will be stored for a maximum of 10 years.

Heparin blood samples (80 ml) will be immediately processed using established procedures (see [75, 76]) for isolation and cryopreservation of peripheral blood mononuclear cells (PBMCs). The protocols for sample processing are harmonized between the labs. PBMCs will be frozen at a concentration of 10^7 cells/ml at -80°C in a pre-cooled freezing container. After 24-48 hours, cells were transferred to liquid nitrogen and stored at -196°C until analysis. Serum samples (5 ml) will be centrifuged, aliquoted, and stored at -20°C until analysis.

- Mitochondrial function of monozytes and T cells
- Cell-specific gene expression relevant for intracellular stress hormone signaling by qRT-PCR
- Phenotyping of monozytes and T cells by fluorescence activated cell sorting (FACS)
- Serum concentrations of cytokines incl. IL-6, TNF, IL-1beta

9.7.3 Other procedures

CGI
The CGI consists of 2 sub-scales: CGI-S and CGI-I. Clinical Global Impression scale – Severity of illness (CGI-S) and Clinical Global Impression scale - Improvement (CGI-I) will be used to assess patient’s overall severity and improvement/worsening of depression [62]. CGI-I of the current visit will be assessed compared to a CGI-I score of the last visit. CGI-S will be conducted at visit 1, 2, 3, 4, 5 and 6, whereas CGI-I will be conducted at visit 2, 3, 4, 5 and 6 by the subinvestigator.

SOFAS
The Social and Occupational Functioning Assessment Scale (SOFAS) will be used to determine social functioning [77] and will be conducted at baseline, week 4 and end-of-study by the subinvestigator.

BMI, measurement of waist circumference
Body-Mass-Index will be determined by measuring weight at screening, baseline and end-of-study by study staff. Height will be measured only once at screening. Waist circumference will be measured at baseline and end-of-study by study staff.

10. Risk-benefit-assessment

Both medications (Simvastatin, Escitalopram) are approved for several indications since decades and have overall been proven to be safe. All depressed patients enrolled in the trial will be continuously treated with a gold standard antidepressant (Escitalopram) as described by the National

Disease Management Guideline ("Nationale Versorgungsleitlinie") for the treatment of MDD. For Escitalopram a dose-response relationship has been suggested. Especially for patients with severe depression the high standard dose of 20 mg/d was shown to be superior compared to 10mg/d [1]. A fixed-dose of 10 mg/d Escitalopram for the entire study duration of 12 weeks would therefore not allow the optimal treatment for some patients. Thus, we consider this option ethically not justifiable. A dose increase from 10 mg/d to 20 mg/d based on individual patient response would however interfere with comparability of the treatment group and placebo group, because a strong decrease in depressive symptoms in the treatment group compared to placebo group could potentially be masked by a higher percentage of Escitalopram dose escalation in the placebo group. Therefore, we will increase Escitalopram to 20 mg/d after giving a low standard dose of 10 mg/d in the first two weeks. In case of adverse reactions related to Escitalopram, we will follow the instructions for dose adjustment described in 7.2.4 Treatment schedule / Dose regimen.

IMP Simvastatin is offered in an "add-on" design, thus there will be virtually no drug-free patients in this trial. Invasive interventions beyond ordinary sampling of peripheral venous blood will not be performed within this trial, therefore the risks associated with trial participation are mainly attributable to IMP Simvastatin that has already been shown in depressed patients to be safe and well tolerated in combination with an SSRI [2]. In a recent Swedish national cohort study statin use was associated with reduced diagnoses of clinical depression [39]. However, antidepressive effects of Simvastatin in patients with comorbid obesity and major depression is not known to our knowledge.

It has been suggested that both for the lipid-lowering effects and for the pleiotropic effects of statins a high dose is more effective than a low dose [3-5]. Especially in studies examining effects of statins on diseases of the central nervous system such as multiple sclerosis and mild cognitive disorder a high dose of Simvastatin (60 – 80 mg/d) has been administered [6-8]. One of the most important side effects of Simvastatin is dose-dependent risk of myopathy. The combined incidence for myopathy/rhabdomyolysis under Simvastatin was 0.03% for 20 mg/d, 0.08% for 40 mg/d and markedly went up to 0.61% for 80 mg/d according to latest version of German "Fachinformation". To minimize the risk of Simvastatin and examine the pleiotropic effects of Simvastatin in depression, we will use it in a medium dose (40 mg/d) and not a high dose (80 mg/d). In addition to assessing safety laboratory values including creatine kinase (CK) at screening, baseline, at week 4, week 8 and week 12, it will be explicitly asked during each visit if the patient experienced unexplained muscle pain or dark urine as a sign of rhabdomyolysis. Furthermore, we will not include particularly vulnerable patients (e.g. patients with renal impairment, untreated hypothyroidism or history of muscle toxicity under statins or fibrates) to our study. To exclude patients with QTc-prolongation an ECG will be conducted at screening. Furthermore, additional safety ECGs will be conducted at every visit.

According to a meta-analysis of small, randomized controlled trials, a beneficial antidepressive effect of Simvastatin is to be expected [2]. Many depressed patients with comorbid obesity might additionally benefit from Simvastatin in terms of primary prevention of cardiovascular disease.

In summary, the potential benefits of the study by far outweigh the risks associated with Escitalopram and Simvastatin. Importantly, a recent extensive literature review on parallel SSRI and statin use concluded the following: "Escitalopram, Citalopram, and Paroxetine are almost certain to be safe with all statins" [9].

11. Termination and subsequent treatment

11.1 Premature termination of the individual participant

Patients are free to withdraw from participation in the study without specifying any reasons. An investigator may discontinue or withdraw a participant from the study for the following reasons:

- If any clinical adverse event (AE), laboratory abnormality, or other medical condition occurs such that continued participation in the study would not be in the best interest of the participant. In particular the following AE, laboratory abnormality, or other medical condition will lead to immediate discontinuation of the individual participant:
    - report of any unexplained muscle pain, tenderness or weakness if accompanied by malaise or fever
    - hyperbilirubinemia or jaundice
    - interstitial lung disease
    - QTc prolongation ≥ 500 ms or ≥ 60 increase from baseline visit
    - seizures
    - activation of mania/hypomania
    - abnormal bleeding event (e.g. ecchymoses, purpura)
    - serotonin syndrome
    - angle closure glaucoma
    - clinically significant worsening of depression severity operationalized as a CGI-I change score of 6 or 7 (“much worse” or “very much worse”) in two successive visits.
    - clinically significant laboratory abnormalities such as
        - elevated creatine kinase greater than 5fold upper limit of normal (ULN)
        - elevated GOT or GPT greater than 3fold upper limit of normal (ULN)
        - severe symptomatic hyponatremia (serum sodium [S_{Na}] <125 mmol/L)
    - acute suicidal ideation (MADRS item 10 > 4)
    - pregnancy
    - active SARS-CoV-2 infection

In the case of premature termination, the reason for withdrawal must be entered on the appropriate case report form (eCRF) page and the patient must be followed for safety and efficacy until the end of the study (week 12).

11.2 Premature termination of the clinical study

According to the German Fachinformation for Simvastatin, the combined incidence for myopathy/rhabdomyolysis in 41.413 patients treated for at least 4 years was 0.08% for 40mg/d Simvastatin. Severe symptomatic hyponatremia (serum sodium [S_{Na}] <125 mmol/L) is a rare but potentially fatal complication of Escitalopram. Signs and symptoms of hyponatremia include headache, difficulty concentrating, memory impairment, confusion, weakness, and unsteadiness and can be as severe as hallucination, syncope, seizure, coma, respiratory arrest, and death. Therefore, the whole study may be discontinued at the occurrence of the treatment-related serious adverse event (SAE) “myopathy/rhabdomyolysis” as defined by the German Fachinformation for Simvastatin [muscle pain, tenderness or weakness with creatine kinase (CK) above ten times the upper limit of normal (ULN)] in more than 5 participants or the treatment-related SAE “severe symptomatic hyponatremia” (serum sodium [S_{Na}] <125 mmol/L) in more than 5 participants. In addition, an early discontinuation of the trial may be decided if new scientific data during the course of the

trial changes the risk-benefit-balance significantly. If such data emerges, recruitment and treatment of currently treated patients will be paused immediately. A final decision on continuation or termination of the trial will then made by the Sponsor/PI based on the recommendation of the Data and Safety Monitoring Board (DSMB). Furthermore, the clinical study will be prematurely terminated if the relevant Ethics Committees withdraws the ethics approval or a required adjustment of the maximum amount of patient insurance is not possible.

11.3 Follow-up and continuing treatment after regular / premature termination

After completing all the protocol treatment and visits, patients will continue with regular visits according to usual practice. In case of response or remission, Escitalopram will be continued while the study medication Simvastatin (IMP) will be discontinued in any event.

In case of non-response or non-remission, Esctalopram will be definitely discontinued. The investigator will ensure a gradual dose reduction of Escitalopram over at least one or two weeks because of potential symptoms associated with abrupt discontinuation of Escitalopram. Patients with non-response or non-remission will be offered at least one additional clinical appointment at the psychiatric outpatient clinic of the study center after termination of the individual study participation for the gradual discontinuation of Escitalopram.

12. Adverse events

12.1 Definitions

12.1.1 Adverse Event (AE)

An adverse event is an untoward medical occurrence in a patient or clinical trial subject administered a medicinal product and which does not necessarily have a causal relationship with this treatment. An AE could be diseases, signs or symptoms which occur or worsen after enrolment of the patient in the clinical trial.

12.1.2 Adverse Reactions (AR)

Adverse reactions are all untoward and unintended responses to an investigational medicinal product related to any dose administered.

12.1.3 Serious Adverse Event (SAE) / Serious Adverse Reaction (SAR)

A serious adverse event or serious adverse reaction is an untoward medical occurrence or effect that at any dose

- results in death,
- is life-threatening,
- requires hospitalisation or prolongation of existing hospitalisation,
- results in persistent or significant disability or incapacity,
- or is a congenital anomaly or birth defect;
- requires a higher benzodiazepine regimen or prescription of antipsychotics (“rescue medication”).

12.1.4 Exceptional rules

In this clinical trial the following AEs/SAEs are excluded from the notification requirement:

- Serious or unexpected events which occur after enrolment, but before treatment was initiated
- Events with hospitalization planned before the inclusion to the trial (i.e. scheduled surgery)

12.1.5 Suspected Unexpected Serious Adverse Reactions (SUSAR)

A Suspected Unexpected Serious Adverse Reaction (SUSAR) is any suspected adverse reaction related to the study treatment that is both serious and unexpected.

“Unexpected” means that the nature and severity of the adverse reaction are not consistent with the information about the study medication in question set out in the reference safety information.

12.2 Treatment of (S)AEs

All AEs should be treated appropriately. Such treatment may include changes in study drug treatment including possible interruption or discontinuation, starting or stopping concomitant treatments, changes in the frequency or nature of assessments, hospitalization or any other medically required intervention.

12.3 Assessment of AEs and SAEs

As far as possible, each AE should be evaluated to determine:

- the severity grade (assessment of intensity)
- whether it constitutes a SAE (see 12.1.3)
- its relationship to the study drug (assessment of causality)
- its duration (start and end dates or if continuing at final exam)
- action taken (no action taken; study drug dosage adjusted/temporarily interrupted; study drug permanently discontinued due to this AE; hospitalization)

12.3.1 Assessment of intensity

We will apply the following definitions according to the Common Terminology Criteria for Adverse Events (CTCAE) grading:

Grade 1 Mild; asymptomatic or mild symptoms; clinical or diagnostic observations only; intervention not indicated.

Grade 2 Moderate; minimal, local or noninvasive intervention indicated; limiting age-appropriate instrumental ADL (Activities of daily living).

Grade 3 Severe or medically significant but not immediately life-threatening; hospitalization or prolongation of hospitalization indicated; disabling; limiting self-care ADL.

Grade 4 Life-threatening consequences; urgent intervention indicated.

Grade 5 Death related to AE.

Seriousness (severity grade) shall be determined according to the definition above. Furthermore, medical and scientific judgment should be exercised in deciding whether expedited reporting is appropriate in other situations, such as important medical events that may not be immediately life-threatening or result in death or hospitalization but may jeopardize the subject or may require intervention to prevent one of the other outcomes listed in the definition above. These should also usually be considered serious. Examples of such events are intensive treatment in an emergency room, or at home for allergic bronchospasm, blood dyscrasias or convulsions that do not result in hospitalisation, or development of drug addiction or drug abuse.

12.3.2 Assessment of causality

To assess causality between administration of the IMP and the Adverse Event the following definitions apply:

Sure: The reaction comprehensively follows the administration of the IMP in the right timeframe or can be measured in body tissues or fluids or represents a known or expected

response to the study medication or disappears after discontinuation or dose reduction and reoccurs after re-exposure.

Probable: The reaction comprehensively follows the application of the IMP in the right timeframe or represents a known or expected response to the study medication or disappears after discontinuation or dose reduction and cannot be explained by known characteristics of the patient's disease.

Possible: The reaction comprehensively follows the application of the IMP in the right timeframe or represents a known or expected response to the study medication, but could easily be caused by other factors.

Not related: Adequate Information supporting the assumption that there is no causality

Cannot be evaluated: The causality cannot be determined.

12.4 Documentation of AEs and SAEs

All AE and SAEs will be documented, no matter if the investigator suspects a causal connection to the IMP or not. The event will be recorded in the eCRF.

Out of normal range laboratory data and ECG abnormalities will be analyzed concerning their clinical relevance by the subinvestigator – and if relevant documented as an AE itself. Related signs, symptoms, and laboratory changes will be summarized to a specific disease. The documentation will include the type of event, start, duration, severity, causality, and action taken.

SAEs need to be documented on a separate SAE form (see 12.5.1). All SAEs need to be followed until they subside or stabilize.

The Sponsor/Sponsor representative will carefully document all Adverse Events (AEs) reported by the investigator.

12.5 Reporting of SAEs and SUSARs

12.5.1 Reporting of SAEs / Contact for SAE reporting

Every event will be documented on a record form and will immediately be sent to the given address. If at that point all required information is not available, succeeding records will be sent. In the event of death, a copy of the autopsy record should be added.

The investigator shall promptly (at the latest within 24 h) notify the Sponsor/Sponsor representative of the occurrence of a Serious Adverse Event after becoming aware and subsequently provide the Sponsor/Sponsor representative with a detailed written report[3].
Contact details for SAE reports:

Charité Universitätsmedizin Berlin
Koordinierungszentrum für klinische Studien /KKS Charité
Central Pharmacovigilance KKS Charité
Fax number: 030 4507553856
Email: pharmacovigilance-kks@charite.de

Personal data shall be pseudonymized before transmission using the identification code of the data subject.
The report must include the following minimal criteria:
- Patient-ID
- Name of reporter/investigator

[3] GCP-V §12 (4), and 2011//C 172/01 (CT-3), 4.3.1, 29.

- Information on event (term, case narrative, duration)
- Assessment of severity and causality (relationship to study drug/study procedure/concomitant medication/other circumstances)

12.5.2 Reporting of Suspected Unexpected Serious Adverse Reactions (SUSARs)

Before reporting a SUSAR (Suspected Unexpected Serious Adverse Reactions) the subject will be unblinded. The Sponsor/Sponsor representative will report all suspicious cases of SUSARs which had been occurred in one of clinical trials conducted by the same Sponsor with the same drug substance/IMP to the relevant Ethics Committee, the relevant regulatory authorities and to relevant regulatory authorities of other European member states and other contracting states of the EWR agreement, if the study is run in their territory immediately, at the latest 15 days after it becomes known. He will also inform all investigators involved in the trial.

In case of a fatal or life threatening SUSAR the Sponsor/Sponsor representative will report all information relevant for judging the event immediately, at the latest 7 days after the event becomes known to the relevant Ethics Committee, the relevant regulatory authorities and to relevant regulatory authorities of other European member states and other contracting states of the EWR agreement, if the study is run in their territory as well as to all investigators involved in the trial. After a further 8 days all further relevant information must be available.

12.5.3 Other safety issues requiring expedited reporting

The Sponsor/Sponsor representative will immediately, at the latest 15 days after it becomes known report all circumstances that require a revision of the risk-benefit analysis to the relevant Ethics Committee, the relevant regulatory authorities and to relevant regulatory authorities of other European member states and other contracting states of the EWR agreement, if the study is run in their territory. This especially includes:

- Singular cases of expected severe adverse events with an unexpected outcome.
- Increased incidence of expected severe adverse events that are judged as being clinically relevant.
- Events related to study procedures or development of the study medication, which could affect a subject’s safety.

All person-related data will always be transmitted pseudonymized.

12.6 Follow-up of adverse events

Once an AE is detected, it will be followed until its resolution or stabilization, and assessment will be made at each visit (or more frequently, if necessary) of any changes in severity, the suspected relationship to the study, the interventions required to treat it and the outcome.

Follow-up information is sent to the same address to which the original SAE Report Form was sent, using a new SAE Report Form stating that this is a follow-up to the previously reported SAE and giving the date of the original report. Each re-occurrence, complication, or progression of the original event will be reported as a follow-up to that event regardless of when it occurs. The follow-up information will describe whether the event has resolved or continues, if and how it was treated, whether the patient continued or withdrew from study participation.

For a follow-up report to the authorities, the investigator may be required to collect further information for a detailed description and a final evaluation of the case, including copies of hospital reports, autopsy reports, or other relevant documents.

12.7 Data Monitoring Committee

We will seek advice from independent experts in the field who have agreed to serve on the Data and Safety Monitoring Board (DSMB):

- Prof. Dr. Brenda Penninx (methods expert/epidemiologist), Vrije Universiteit Amsterdam
- Prof. Dr. Falk Kiefer (clinician), Zentralinstitut für Seelische Gesundheit, Mannheim
- Prof. Dr. Carsten Spitzer (clinician), Klinik für Psychosomatik und Psychotherapeutische Medizin Universitätsmedizin Rostock
- Mrs. Francoise Margue (patient representative), Deutsche Depressionsliga

To ensure that the patients' perspective will be present at all DSMB meetings as recommended by the European Patient Academy [78], Mrs. Francoise Margue, vice-chair of the "Deutsche Depressionsliga" has kindly agreed to serve on the DSMB. The DSMB will regularly (once a year) assess safety data during the course of this study and their primary interest is the safety of study participants and integrity and validity of collected data.

13. Documentation

13.1 Source Data and Study Files

The investigator must maintain source documents for each patient in the study (see 15.1).

13.2 Case Report Forms (CRF)

Investigators or their designee must enter the information required by the protocol onto the electronic case report forms - eCRF (see 15.2).

13.3 Investigator Site File (ISF)

All essential documents will be kept in the Investigator Site File, which will be stored at the study sites in accordance with ICH GCP chapter 8.

13.4 Drug accountability / Flow of study medication

Study medication will be provided by Charité pharmacy. Receipt of study medication will be documented in each study site (date, number of medication received, charge-ID, etc.) by the investigator. The individual patient will receive the medication from the subinvestigator. This will be documented in the drug account (date, charge-ID). Medication that is not used and empty medication boxes will be stored until final closure of the drug account by the clinical study monitor.

14. Quality management

14.1 Control of trial progress and data quality

The study sites will be monitored by a clinical study monitor to ensure the quality of the data collected. The objectives of the monitoring procedures are to ensure that the trial subject's safety and rights as a study participant are respected, that accurate, valid and complete data are collected, and that the trial is conducted in accordance with the trial protocol, the principles of GCP and local legislation.

14.1.1 Monitoring, Data Quality Assurance

All investigators agree that the monitor regularly visits the study site and assure that the monitor will receive appropriate support in his activities at the study site. The declaration of informed consent includes a statement to the effect that the monitor has the right – while observing the provisions of data protection legislation – to compare the case report forms (eCRFs) with the trial subject’s study files (doctor’s notes, ECGs, laboratory printouts etc.). The investigator will secure access for the monitor to all necessary documentation for trial-related monitoring. The aims of the monitoring visits are as follows:

- To check the declarations of informed consent.
- To monitor trial subject safety (occurrence and documentation/reporting of AEs and SAEs).
- To check the completeness and accuracy of entries on the eCRF.
- To validate the entries on the eCRF against those in the source documents (source data verification, SDV).
- To evaluate the progress of the trial.
- To evaluate compliance with the trial protocol.
- To assess whether the trial is being performed according to GCP at the study site.
- To discuss with the investigator aspects of trial conduct and any deficiencies found.

A monitoring visit report is prepared for each visit describing the progress of the clinical trial and any problems (e.g. refusal to give access to documentation).

The investigators allow the monitor to have access to any or all of the study materials needed for source data verification and proper review of the study progress. At all times, the Sponsor/investigators/monitors will maintain the confidentiality of the study documents. Furthermore, problems with inconsistent and incomplete data will be discussed. By signing the declaration of informed consent, the participants allow access to their documents.

14.1.2 Audits / Inspections

Authorized representatives of the Sponsor, a regulatory authority, or an Independent Ethics Committee (IEC) may visit the center to perform audits or inspections, including source data verification. The purpose of a Sponsor audit or inspection is to systematically and independently examine all study related activities and documents to determine whether these activities were conducted, and data were recorded, analyzed, and accurately reported according to the protocol, Good Clinical Practice (GCP), guidelines of the International Conference on Harmonisation (ICH), and any applicable regulatory requirements.

With the signature in the protocol, the investigators confirm that auditors and health authority inspectors may have access to the study documentation and accordant medical records. Auditors and inspectors are bound by professional confidentiality and may not pass on any personal information that comes to their knowledge. In the course of audits or inspections, data in the case report forms will be compared with the data for medical records. All the documentation held by the investigators within the scope of the clinical trial, as well as the drug logs of the study medications will be verified.

14.2 Standardisation and validation

All rating scales have previously been standardized and validated.

14.3 Reference institutions

Not applicable.

15. Data entry und data management

Patient related data will be recorded in study files (source data) under the participant`s real name. The information required by the protocol will be entered onto the electronic case report forms (eCRF - secuTrial) under a pseudonym. Every patient will receive a patient number / pseudonym which will be unique for this individual patient (see 9.3).

15.1 Source Data and Study Files

The investigator must maintain source documents for each patient in the study, consisting of all demographic and medical information, including laboratory data, electrocardiograms, and all assessments and keep them together with the signed informed consent form in designated study files. The information in original documents and records defined as Source Data will be reviewed by the Monitor for Source Data Verification.

15.2 Data collection / Case Report Form (CRF)

Investigators or their designee must enter the information required by the protocol onto the electronic case report forms (eCRF - secuTrial®). Data items from the eCRF are entered into the study database. All entries to the eCRF must be made as described in the electronic case report form manual. All information on eCRF must be traceable to source documents in the study file.

A clinical study monitor will review the eCRF and compare the content to the source data. In addition, the investigator or designated colleague will provide access to designated Sponsor representative(s) for the periodic review of source documents (*e.g.* hospital and clinic records) to assure accuracy and completeness of the eCRF.

15.3 Data management

Data required for the analysis will be acquired and transferred electronically to a central database at the KKS Charité by means of an electronic data capture system (EDC) - secuTrial®.

The secuTrial® Software solution of interActive Systems (iAS) is a worldwide reachable web-based system (remote data entry [RDE]). The system operates according to the principle of online data capture and is in compliance with the FDA 21 CFR Part 11 and ICH-GCP. In addition, it contains further functions to perform plausibility, consistency and range checks of the study data.

The log-in in the system requires a previous authentication for access and runs by a secure data transfer-protocol (SSL) to exclude any improper manipulation of data by unauthorized parties. The system will automatically keep an audit trail of all entries and corrections to the eCRF.

The secuTrial® Software solution is based on an Oracle database that does not allow direct access. All access rights of study staff will be defined according to their trial functions. The defined access rights will *e.g.* allow to read or to enter study data depending on their functions (Monitor, clinical investigator, etc.).

Query management is an integrated part of this RDE system. This function allows the communication between the Monitor and study staff. In case of errors or improper data, the responsible person of study staff will get queries (messages) to correct the errors or to check improper data on the eCRF.

The trial data will be stored digitally on a remote server with daily backups. After end of study all data will be exported for checks of consistency and plausibility. After performing the checks, the data matrix will be transferred in pseudonymized form for the statistical evaluation.

16. Statistical Analysis

16.1 Hypotheses

16.1.1 Primary hypothesis

Simvastatin (40 mg/d) add-on to the standard antidepressant Escitalopram (20 mg/d) improves depression to a greater extent over 12 weeks than add-on placebo in patients with comorbid obesity and major depression

16.1.2 Secondary hypotheses

Add-on Simvastatin (40 mg/d) to standard antidepressant Escitalopram (20 mg/d) will improve:

1) response rates, and remission rates,

2) patients' impression of change, clinician's impression of severity and change, quality of life, social functioning, self-report depression,

3) lipid values,

4) mitochondrial and cellular function of of immune cells

to a greater extent over 12 weeks than adjunct placebo in patients with major depression and comorbid obesity.

16.2 Sample size estimation

So far, there are three small pilot RCTs in depressed patients (sample sizes ranging from n = 48 to n = 68) that have all found statistically significant beneficial effects of add-on statins vs. placebo with large, clinically relevant effect sizes. A meta-analysis [2] of these trials has found a standardized mean difference between add-on statins and add-on placebo of -0.73 (95 % confidence interval -1.04 to -0.42, p < 0.001). However, it is well-known that initial studies tend to overestimate the true effect size [79]. Therefore, we conservatively assume a standardized treatment difference (also known as Cohen's d) of 0.5, which is supported by the findings of all pilot RCTs [36, 80, 81]. Given a standard deviation of 6-8 points on the MADRS, as commonly observed in RCTs in this population [82-84], a standardized effect size of 0.5 translates into a mean difference of 3-4 points, which is considered clinically relevant because it is about twice as large as the minimal clinically important difference of 1.6 to 1.9 points that has been reported [85]. Thus, a sample size of 64 patients per group gives a power of 80% for a comparison of the mean changes in MADRS score from baseline to week 12 between the two treatment groups using a two-sample t-test at the usual two-sided level of 5%. As the analyses will be adjusted for baseline scores the actual power is likely to be higher; this will be confirmed in a blinded sample size review once 50% of the patients have reached week 12 [86]. Accounting for about 20% dropout, we aim to recruit 80 patients per group (i.e. 160 patients in total). Based on sex-specific prevalence rates and recruitment in our earlier studies [87, 88], we expect that about two thirds of the sample will be women. This will allow gender-specific analyses.

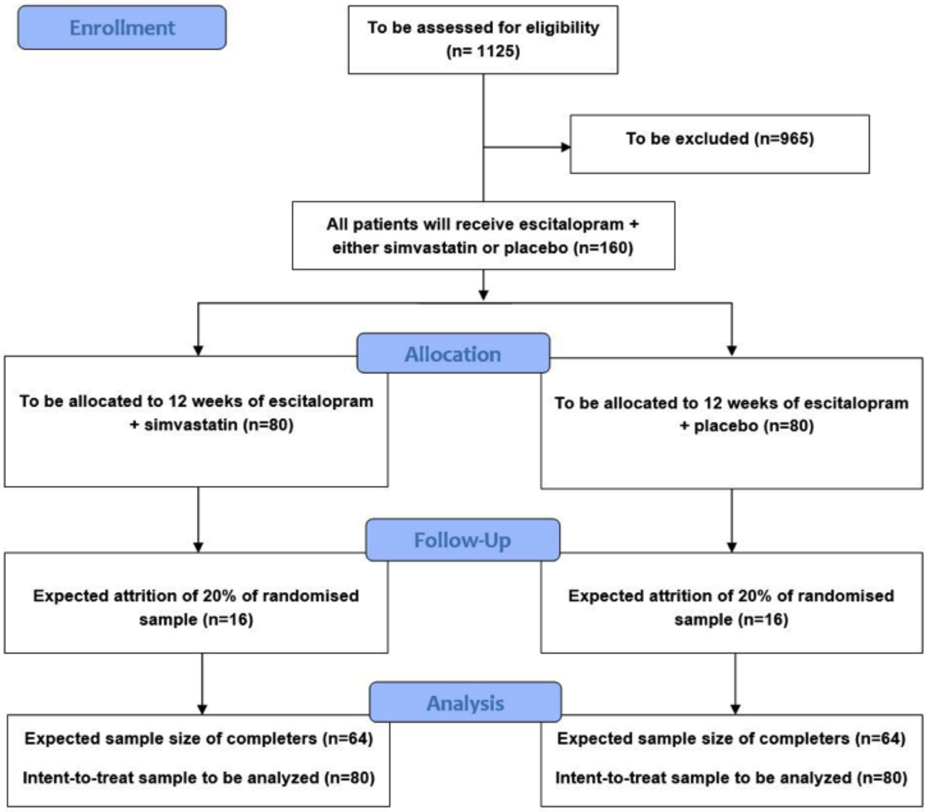

Figure 4: *Study flow chart according to CONSORT. We assume that we need to screen N = 1125 patients aged 18 - 65 with major depression according to Diagnostic and Statistical Manual of Mental Disorders (DSM) 5 and obesity (body mass index ≥ 30) for eligibility at 8 recruiting centers to randomize n = 160 participants. The sample size and assumed dropout rate (20%) is conservatively based on three earlier pilot RCTs of add-on statin to SSRI treatment in depressed patients [1-3].*

16.3 Definition of population for analysis

The primary analysis population will be the intention-to-treat (ITT) population. The ITT population will include all randomized patients. We will repeat the analyses in the per-protocol sample.

16.4 Analysis of primary and secondary endpoints

The primary analysis will compare MADRS changes from baseline to week 12 between the Simvastatin and placebo group by Gaussian linear models for repeated measures (so-called MMRM) with intervention, center, time (week 1, 2, 4, 8, and 12), and intervention-by-time interaction as factors, and baseline MADRS score as covariate. The error terms are assumed to follow a multivariate normal distribution with unstructured covariance. Least squares mean changes from baseline will be reported for both groups with 95% confidence interval (CI) as well as the difference between the least squares treatment group means with 95% CI and p-value testing the null hypothesis of no treatment effect. Although the model described above is robust to a certain extend to missing data, sensitivity analyses will be performed as supporting analyses including multiple imputation and last-observation-carried-forward (LOCF). The latter will also facilitate comparison with previous trials.

The analyses of all secondary endpoints and safety parameters will have an exploratory character and will therefore not be adjusted for multiple testing. The secondary efficacy endpoints MADRS-response and MADRS-remission, and MADRS-MCID within 12 weeks will be analyzed by logistic regression models with intervention and center as factors and baseline MADRS as covariate. The treatment effects will be reported as odds ratios with 95% CI and p-values testing the null hypotheses of no treatment effect. The analyses of continuous secondary efficacy endpoint as well as SOFAS, and EQ-5D-3L will follow the same lines as the primary outcome.

We will repeat the analyses in the per-protocol sample.

All details of the statistical analyses including definitions of the analyses populations will be specified in the statistical analysis plan, which will be finalized before database lock and unblinding. All statistical analyses will be carried out using SAS software (SAS Institute Inc., Cary, NC, USA) or the R package.

16.5 Analysis of adverse events

All adverse events (AE; any undesirable experience occurring to a participant during the trial, whether or not considered related to the study medication) reported by the subject or observed by the investigators will be recorded at each visit. Standard procedures for reporting of (serious) adverse events will be used. (Serious) adverse events will be summarized as frequencies and percentages by treatment group. For recurrent events and to account for variable follow-up times event rates will be reported with rate ratios comparing Simvastatin with add-on placebo control and 95% confidence intervals. We will use Poisson regression models with adjustment for over dispersion (or negative binomial regression models) with offset for follow-up time and possibly a mixture component to account for zero-inflation. For events of particular interest Kaplan-Meier curves stratified by treatment group will be computed and compared by log-rank tests.

16.6 Interim analyses and endpoints for premature study termination

As the analyses will be adjusted for baseline scores the actual power is likely to be higher than 80 %. To confirm this in an interim analysis, a blinded sample size review will be carried out once 50 % of the patients have reached week 12. Based on blinded estimates of the mean square error and the dropout rate the required sample size will be recalculated and the sample size will be adjusted up to a maximum total sample size of 240 patients, if necessary.

17. Reporting

17.1 Statistical report

The statistical analysis and the biometrical report will be provided by the biostatistician Prof. Dr. Tim Friede, Universitätsklinikum Göttingen, in cooperation with the Sponsor representative / Principal investigator. All data in this report is confidential.

17.2 Final report

The composition of a final integrated report will be conducted in accordance with ICH E3: Structure and Contents of Clinical Study Reports. After termination of the biometrical analysis, the biostatistician will compose an integrated report. This report contains a clinical record, a statistical record, single value tables and conclusions. It will be signed by the principal investigator and the biostatistician.

17.3 Publication (policy)

The study results will be published irrespective of the study outcome.

18. Ethical, legal and regulatory aspects

18.1 ICH-GCP-guidelines

This trial will be conducted in accordance with the current ICH-GCP-guidelines. Good Clinical Practice (GCP) is an international ethical and scientific quality standard for designing, conducting, recording and reporting trials that involve the participation of human subjects. Compliance with this standard provides public assurance that the rights, safety and well-being of trial subjects are protected, consistent with the principles that have their origin in the Declaration of Helsinki, and that the clinical trial data are credible.

18.2 Legal requirements of the study

18.2.1 Approval of Ethics Committee

Study protocol, patient information (Information zur Studie) and consent form (Einwilligungserklärung) will be presented to the relevant Ethics Committees for survey. The study will only start after ethics approval has been granted. The Ethics Committee will immediately be informed (by the Sponsor/Sponsor representative) of all changes to the protocol (according to GCP-V § 10) and of all events that could affect a patient’s safety. The Ethics Committee will also be informed of all suspected SUSARs and of regular or premature termination of the study. We will register our study in the ClinicalTrials.gov database and German Clinical Trials Register (DRKS) before we enroll any patients.

18.2.2 Approval of competent authority

The trial will be submitted to the relevant federal authorities (BfArM) for approval. The trial will only start after approval has been granted.

18.2.3 Notification to regional authorities

The trial will be submitted to the relevant state authorities by the Sponsor representative / Principal investigator before we enroll any patients. The Sponsor/Sponsor representative and all investigators will be reported by full name.

18.2.4 Patient information and informed consent

Patient Information
Before enrolment every patient will receive full oral and written information about the nature, purpose, expected advantages and possible risks of the trial.

Consent to participation in the trial:
The patient will agree to participation in the trial by signing the informed consent form. Patients must be given an opportunity to enquire about details of the study. After a sufficient period of time for the individual’s consideration and decision, comprehension and consent shall be documented on the consent form by the dated signature of the patient and the investigator/subinvestigator. Design and language will be adjusted to the study site’s needs. The final versions of patient information and consent will be presented to the Ethics Committee. Both the patient information and the patient consent form are prepared in duplicate. One of each form for the investigator, a duplicate will be handed to the patient.

18.2.5 Patient insurance

For participating patients, an insurance (according to AMG § 40 Absatz 1 Satz 3 No. 8) was

obtained with the

HDI Global SE
HDI-Platz 1, 30659 Hannover
Tel. 0211 - 74820
Policy number 43125625 03680

The insurance is limited to a maximum of 500.000 Euro per patient and 50.000.000 Euro for the whole study. A copy of the insurance policy and the insurance terms and conditions will be handed to the participants.

18.2.6 Data Privacy and confidentiality

The participants' data will be saved in a pseudonymous form, which will neither contain initials nor full date of birth. All regulative requirements applying to data protection will be met. Re-identification of a participant subject's name is possible from the patient identification log, which is kept in a locked research office at the study site where access is only possible by the investigator or persons authorized by the investigator.

Patients will be informed that their disease-related data will be saved for scientific purpose (publication, etc.) using a pseudonym. Consenting patients have got the right to be informed about the data recorded. Patients will also be informed that their pseudonymized data will be forwarded to the federal authorities (BfArM) and to the Ethics Committee responsible, in accordance with legal notification obligation for drug safety. Patients, who disagree with this process of data transfer, are not allowed to participate in this study.

Blood samples will be pseudonymized and sent to the local laboratories for analysis. They will be destroyed after analysis (laboratory safety, laboratory assessment lipid) or will be stored for a maximum of 10 years (laboratory assessment immunometabolism / mitochondrial function). The pseudonymized data will be transferred to the study site.

18.3 Archiving of data / Access to records

Essential documents, as listed below, must be retained by the investigator for as long as needed to comply with national and international regulations. The investigator agrees to adhere to the document retention procedures by signing the protocol. Essential documents include:

- IRB/IEC approvals for the study protocol and all amendments
- All source documents and laboratory records
- Patient identification log
- CRF copies (paper copies or electronic copies on a CD-ROM)
- Signed patient informed consent forms
- Any other pertinent study document.

Originals of all study-related report (incl. original data of study patient - medical records) forms will be stored in each study site for at least 10 years after completion of the trial.

A list allowing patient identification will be kept for 10 years.

18.4 Financing

The study is funded by the *Bundesministerium für Forschung und Bildung* (BMBF; Federal Ministry of Education and Research) within the call „Klinische Studien mit besonderer Relevanz für die Patientenversorgung“

19. References

1. Bech, P., et al., *Escitalopram dose-response revisited: an alternative psychometric approach to evaluate clinical effects of escitalopram compared to citalopram and placebo in patients with major depression.* Int J Neuropsychopharmacol, 2004. **7**(3): p. 283-90.
2. Salagre, E., et al., *Statins for the treatment of depression: A meta-analysis of randomized, double-blind, placebo-controlled trials.* J Affect Disord, 2016. **200**: p. 235-42.
3. Wang, C.-Y., P.-Y. Liu, and J.K. Liao, *Pleiotropic effects of statin therapy: molecular mechanisms and clinical results.* Trends in molecular medicine, 2008. **14**(1): p. 37-44.
4. de Lemos, J.A., et al., *Early intensive vs a delayed conservative simvastatin strategy in patients with acute coronary syndromes: phase Z of the A to Z trial.* Jama, 2004. **292**(11): p. 1307-16.
5. Wu, C.K., et al., *Statin use reduces the risk of dementia in elderly patients: a nationwide data survey and propensity analysis.* J Intern Med, 2015. **277**(3): p. 343-352.
6. Vollmer, T., et al., *Oral simvastatin treatment in relapsing-remitting multiple sclerosis.* Lancet, 2004. **363**(9421): p. 1607-8.
7. Chataway, J., et al., *Effect of high-dose simvastatin on brain atrophy and disability in secondary progressive multiple sclerosis (MS-STAT): a randomised, placebo-controlled, phase 2 trial.* Lancet, 2014. **383**(9936): p. 2213-21.
8. ClinicalTrials.gov. *Identifier NCT00842920, Trial of Simvastatin in Amnestic Mild Cognitive Impairment (MCI) Patients (SIMaMCI).* 2019 May 23]; Available from: https://clinicaltrials.gov/ct2/show/NCT00842920.
9. Andrade, C., *Selective serotonin reuptake inhibitor drug interactions in patients receiving statins.* J Clin Psychiatry, 2014. **75**(2): p. e95-9.
10. Abajobir, A.A., et al., *Global, regional, and national incidence, prevalence, and years lived with disability for 328 diseases and injuries for 195 countries, 1990–2016: a systematic analysis for the Global Burden of Disease Study 2016.* The Lancet. **390**(10100): p. 1211-1259.
11. Whiteford, H.A., et al., *Global burden of disease attributable to mental and substance use disorders: findings from the Global Burden of Disease Study 2010.* Lancet, 2013. **382**(9904): p. 1575-86.
12. Busch, M.A., et al., *[Prevalence of depressive symptoms and diagnosed depression among adults in Germany: results of the German Health Interview and Examination Survey for Adults (DEGS1)].* Bundesgesundheitsblatt Gesundheitsforschung Gesundheitsschutz, 2013. **56**(5-6): p. 733-9.
13. Laursen, T.M., et al., *Mortality and life expectancy in persons with severe unipolar depression.* J Affect Disord, 2016. **193**: p. 203-7.
14. Truthmann, J., et al., *Metabolic Health in Relation to Body Size: Changes in Prevalence over Time between 1997-99 and 2008-11 in Germany.* PLoS One, 2016. **11**(11): p. e0167159.
15. Global BMI Mortality Collaboration, et al., *Body-mass index and all-cause mortality: individual-participant-data meta-analysis of 239 prospective studies in four continents.* Lancet, 2016. **388**(10046): p. 776-86.
16. Ng, M., et al., *Global, regional, and national prevalence of overweight and obesity in children and adults during 1980-2013: a systematic analysis for the Global Burden of Disease Study 2013.* Lancet, 2014. **384**(9945): p. 766-81.
17. Luppino, F.S., et al., *Overweight, obesity, and depression: a systematic review and meta-analysis of longitudinal studies.* Arch Gen Psychiatry, 2010. **67**(3): p. 220-9.
18. Robinson, J.G. and N.J. Stone, *The 2013 ACC/AHA guideline on the treatment of blood cholesterol to reduce atherosclerotic cardiovascular disease risk: a new paradigm supported by more evidence.* Eur Heart J, 2015. **36**(31): p. 2110-2118.
19. Otte, C., et al., *Major depressive disorder.* Nat Rev Dis Primers, 2016. **2**: p. 16065.

20. Vogelzangs, N., et al., *Metabolic depression: a chronic depressive subtype? Findings from the InCHIANTI study of older persons.* J Clin Psychiatry, 2011. **72**(5): p. 598-604.
21. Vogelzangs, N., et al., *Inflammatory and metabolic dysregulation and the 2-year course of depressive disorders in antidepressant users.* Neuropsychopharmacology : official publication of the American College of Neuropsychopharmacology, 2014. **39**(7): p. 1624-34.
22. Kloiber, S., et al., *Overweight and Obesity Affect Treatment Response in Major Depression.* Biological Psychiatry, 2007. **62**(4): p. 321.
23. Shitara, Y. and Y. Sugiyama, *Pharmacokinetic and pharmacodynamic alterations of 3-hydroxy-3-methylglutaryl coenzyme A (HMG-CoA) reductase inhibitors: drug-drug interactions and interindividual differences in transporter and metabolic enzyme functions.* Pharmacol Ther, 2006. **112**(1): p. 71-105.
24. Oesterle, A., U. Laufs, and J.K. Liao, *Pleiotropic Effects of Statins on the Cardiovascular System.* Circ Res, 2017. **120**(1): p. 229-243.
25. Kilic, F.S., et al., *Acute antidepressant and anxiolytic effects of simvastatin and its mechanisms in rats.* Neurosciences (Riyadh), 2012. **17**(1): p. 39-43.
26. McFarland, A.J., et al., *Molecular mechanisms underlying the effects of statins in the central nervous system.* Int J Mol Sci, 2014. **15**(11): p. 20607-37.
27. Reinoso, R.F., et al., *Preclinical pharmacokinetics of statins.* Methods Find Exp Clin Pharmacol, 2002. **24**(9): p. 593-613.
28. Gerson, R.J., et al., *Animal safety and toxicology of simvastatin and related hydroxy-methylglutaryl-coenzyme A reductase inhibitors.* Am J Med, 1989. **87**(4A): p. 28S-38S.
29. Smith, P.F., et al., *HMG-CoA reductase inhibitor-induced myopathy in the rat: cyclosporine A interaction and mechanism studies.* J Pharmacol Exp Ther, 1991. **257**(3): p. 1225-35.
30. Minsker, D.H., et al., *Mevalonate supplementation in pregnant rats suppresses the teratogenicity of mevinolinic acid, an inhibitor of 3-hydroxy-3-methylglutaryl-coenzyme a reductase.* Teratology, 1983. **28**(3): p. 449-56.
31. Dostal, L.A., J.L. Schardein, and J.A. Anderson, *Developmental toxicity of the HMG-CoA reductase inhibitor, atorvastatin, in rats and rabbits.* Teratology, 1994. **50**(6): p. 387-94.
32. Merck & Co., I., *Zocor (simvastatin): Highlights of prescribing information*. 2018.
33. Lewington, S., et al., *Blood cholesterol and vascular mortality by age, sex, and blood pressure: a meta-analysis of individual data from 61 prospective studies with 55,000 vascular deaths.* Lancet, 2007. **370**(9602): p. 1829-39.
34. Kolovou, G.D., et al., *Simvastatin: two decades in a circle.* Cardiovasc Ther, 2008. **26**(2): p. 166-78.
35. *Randomised trial of cholesterol lowering in 4444 patients with coronary heart disease: the Scandinavian Simvastatin Survival Study (4S).* Lancet, 1994. **344**(8934): p. 1383-9.
36. Gougol, A., et al., *Simvastatin as an adjuvant therapy to fluoxetine in patients with moderate to severe major depression: A double-blind placebo-controlled trial.* J Psychopharmacol, 2015. **29**(5): p. 575-81.
37. Young-Xu, Y., et al., *Long-term statin use and psychological well-being.* J Am Coll Cardiol, 2003. **42**(4): p. 690-7.
38. Abbasi, S.H., et al., *Simvastatin versus atorvastatin for improving mild to moderate depression in post-coronary artery bypass graft patients: A double-blind, placebo-controlled, randomized trial.* Journal of affective disorders, 2015. **183**: p. 149-155.
39. Molero, Y., et al., *Associations between statin use and suicidality, depression, anxiety, and seizures: a Swedish total-population cohort study.* The Lancet Psychiatry, 2020. **7**(11): p. 982-990.
40. Vuletic, S., et al., *Statins of different brain penetrability differentially affect CSF PLTP activity.* Dement Geriatr Cogn Disord, 2006. **22**(5-6): p. 392-8.

41. Pedersen, T.R. and J.A. Tobert, *Simvastatin: a review.* Expert Opin Pharmacother, 2004. **5**(12): p. 2583-96.
42. Kohler, O., et al., *The Effect of Concomitant Treatment With SSRIs and Statins: A Population-Based Study.* Am J Psychiatry, 2016. **173**(8): p. 807-15.
43. Apostolopoulou, M., A. Corsini, and M. Roden, *The role of mitochondria in statin-induced myopathy.* Eur J Clin Invest, 2015. **45**(7): p. 745-54.
44. Sattar, N., et al., *Statins and risk of incident diabetes: a collaborative meta-analysis of randomised statin trials.* Lancet, 2010. **375**(9716): p. 735-42.
45. Karalis, D.G., et al., *The risks of statin use in pregnancy: A systematic review.* J Clin Lipidol, 2016. **10**(5): p. 1081-90.
46. Kashani, A., et al., *Risks associated with statin therapy: a systematic overview of randomized clinical trials.* Circulation, 2006. **114**(25): p. 2788-97.
47. Riaz, H., et al., *Meta-analysis of Placebo-Controlled Randomized Controlled Trials on the Prevalence of Statin Intolerance.* Am J Cardiol, 2017. **120**(5): p. 774-781.
48. Harper, C.R. and T.A. Jacobson, *Evidence-based management of statin myopathy.* Curr Atheroscler Rep, 2010. **12**(5): p. 322-30.
49. Kohler, O., et al., *The Effect of Concomitant Treatment With SSRIs and Statins: A Population-Based Study.* Am J Psychiatry, 2016: p. appiajp201615040463.
50. Serretti, A. and L. Mandelli, *Antidepressants and body weight: a comprehensive review and meta-analysis.* J Clin Psychiatry, 2010. **71**(10): p. 1259-72.
51. Parsaik, A.K., et al., *Statins use and risk of depression: a systematic review and meta-analysis.* Journal of affective disorders, 2014. **160**: p. 62-7.
52. Otte, C., S. Zhao, and M.A. Whooley, *Statin use and risk of depression in patients with coronary heart disease: longitudinal data from the Heart and Soul Study.* J Clin Psychiatry, 2012. **73**(5): p. 610-5.
53. Wium-Andersen, I.K., et al., *Anti-inflammatory treatment and risk of depression in 91,842 patients with acute coronary syndrome and 91,860 individuals without acute coronary syndrome in Denmark.* Int J Cardiol, 2017. **246**: p. 1-6.
54. Wium-Andersen, I.K., et al., *Anti-inflammatory treatment and risk for depression after first-time stroke in a cohort of 147 487 Danish patients.* J Psychiatry Neurosci, 2017. **42**(5): p. 160244.
55. Wittchen, H.U., et al., *The size and burden of mental disorders and other disorders of the brain in Europe 2010.* Eur Neuropsychopharmacol, 2011. **21**(9): p. 655-79.
56. Murray, C.J., et al., *Global, regional, and national disability-adjusted life years (DALYs) for 306 diseases and injuries and healthy life expectancy (HALE) for 188 countries, 1990-2013: quantifying the epidemiological transition.* Lancet, 2015.
57. Rush, A.J., et al., *Acute and longer-term outcomes in depressed outpatients requiring one or several treatment steps: a STAR*D report.* Am J Psychiatry, 2006. **163**(11): p. 1905-17.
58. Montgomery, S.A. and M. Asberg, *A new depression scale designed to be sensitive to change.* Br J Psychiatry, 1979. **134**: p. 382-9.
59. Brozek, J.L., G.H. Guyatt, and H.J. Schunemann, *How a well-grounded minimal important difference can enhance transparency of labelling claims and improve interpretation of a patient reported outcome measure.* Health Qual Life Outcomes, 2006. **4**: p. 69.
60. Duru, G. and B. Fantino, *The clinical relevance of changes in the Montgomery–Asberg Depression Rating Scale using the minimum clinically important difference approach.* Current medical research and opinion, 2008. **24**(5): p. 1329-1335.
61. Button, K.S., et al., *Minimal clinically important difference on the Beck Depression Inventory--II according to the patient's perspective.* Psychol Med, 2015. **45**(15): p. 3269-79.

62. Guy, W., *ECDEU Assessment Manual for Psychopharmacology*. 1976, Rockville, MD: US Department of Health, Education, and Welfare Public Health Service Alcohol, Drug Abuse, and Mental Health Administration.
63. Walters, S.J. and J.E. Brazier, *Comparison of the minimally important difference for two health state utility measures: EQ-5D and SF-6D.* Qual Life Res, 2005. **14**(6): p. 1523-32.
64. Heart Protection Study Collaborative, G., *MRC/BHF Heart Protection Study of cholesterol lowering with simvastatin in 20,536 high-risk individuals: a randomised placebo-controlled trial.* Lancet, 2002. **360**(9326): p. 7-22.
65. http://www.fda.gov/ForConsumers/ConsumerUpdates/ucm257884.htm.
66. European Medicines Agency. 2013 [cited 2016 September 2nd]; Available from: http://www.ema.europa.eu/docs/en_GB/document_library/Scientific_guideline/2013/05/WC500143770.pdf.
67. Chan, D., et al., *Effect of high-dose simvastatin on cognitive, neuropsychiatric, and health-related quality-of-life measures in secondary progressive multiple sclerosis: secondary analyses from the MS-STAT randomised, placebo-controlled trial.* Lancet Neurol, 2017. **16**(8): p. 591-600.
68. Williams, J.B. and K.A. Kobak, *Development and reliability of a structured interview guide for the Montgomery Asberg Depression Rating Scale (SIGMA).* Br J Psychiatry, 2008. **192**(1): p. 52-8.
69. Schmidtke, A., et al., *[Studies of the reliability and validity of the German version of the Montgomery-Asberg Depression Rating Scale (MADRS)]*. Schweiz Arch Neurol Psychiatr (1985), 1988. **139**(2): p. 51-65.
70. Beck, A., et al., *An inventory for measuring depression.* Archives of General Psychiatry, 1961. **4**: p. 561-571.
71. Drug and Therapeutics Bulletin, *QT interval and drug therapy*. BMJ, 2016. **353**: p. i2732.
72. Sheehan, D.V., et al., *The Mini-International Neuropsychiatric Interview (M.I.N.I.): the development and validation of a structured diagnostic psychiatric interview for DSM-IV and ICD-10.* J Clin Psychiatry, 1998. **59 Suppl 20**: p. 22-33;quiz 34-57.
73. Guy, W., *ECDEU Assessment Manual for Psychopharmacology*. 1976, Rockville: National Institue of Mental Health.
74. Rabin, R. and F. de Charro, *EQ-5D: a measure of health status from the EuroQol Group.* Ann Med, 2001. **33**(5): p. 337-43.
75. Hasselmann, H., et al., *Pro-inflammatory monocyte phenotype and cell-specific steroid signaling alterations in unmedi-cated patients with major depressive disorder.* Front Immunol, in press.
76. Patas, K., et al., *T Cell Phenotype and T Cell Receptor Repertoire in Patients with Major Depressive Disorder.* Front Immunol, 2018. **9**: p. 291.
77. Morosini, P.L., et al., *Development, reliability and acceptability of a new version of the DSM-IV Social and Occupational Functioning Assessment Scale (SOFAS) to assess routine social functioning.* Acta Psychiatr Scand, 2000. **101**(4): p. 323-9.
78. https://www.eupati.eu/de/. accessed September 10th 2017.
79. Leon, A.C., L.L. Davis, and H.C. Kraemer, *The role and interpretation of pilot studies in clinical research.* J Psychiatr Res, 2011. **45**(5): p. 626-9.
80. Ghanizadeh, A. and A. Hedayati, *Augmentation of fluoxetine with lovastatin for treating major depressive disorder, a randomized double-blind placebo controlled-clinical trial.* Depress Anxiety, 2013. **30**(11): p. 1084-8.
81. Haghighi, M., et al., *In a randomized, double-blind clinical trial, adjuvant atorvastatin improved symptoms of depression and blood lipid values in patients suffering from severe major depressive disorder.* J Psychiatr Res, 2014. **58**: p. 109-14.
82. Cipriani, A., et al., *Duloxetine versus other anti-depressive agents for depression.* Cochrane Database Syst Rev, 2012. **10**: p. CD006533.

83. Galizia, I., et al., *S-adenosyl methionine (SAMe) for depression in adults.* Cochrane Database Syst Rev, 2016. **10**: p. CD011286.
84. Koesters, M., et al., *Vortioxetine for depression in adults.* Cochrane Database Syst Rev, 2017. **7**: p. CD011520.
85. Duru, G. and B. Fantino, *The clinical relevance of changes in the Montgomery-Asberg Depression Rating Scale using the minimum clinically important difference approach.* Curr Med Res Opin, 2008. **24**(5): p. 1329-35.
86. Friede, T. and M. Kieser, *Blinded sample size recalculation for clinical trials with normal data and baseline adjusted analysis.* Pharm Stat, 2011. **10**(1): p. 8-13.
87. Otte, C., et al., *Modulation of the mineralocorticoid receptor as add-on treatment in depression: a randomized, double-blind, placebo-controlled proof-of-concept study.* Journal of Psychiatric Research, 2010. **44**(339-46).
88. Otte, C., et al., *Mineralocorticoid receptor stimulation improves cognitive function and decreases cortisol secretion in depressed patients and healthy individuals.* Neuropsychopharmacology : official publication of the American College of Neuropsychopharmacology, 2015. **40**(2): p. 386-93.

20. Appendices

20.1 Stellungnahme zur Geschlechterverteilung

Based on sex-specific prevalence rates and recruitment in our earlier studies [87, 88], we expect that about two thirds of the sample will be women. This will allow gender-specific analyses.

20.2 Stellungnahme zur Weiterbehandlung nach Studienende

After completing all the protocol treatment and visits, patients will continue with regular visits according to usual practice. In case of response or remission, Escitalopram will be continued while the study medication Simvastatin (IMP) will be discontinued in any event. In case of non-response or non-remission, Esctalopram will be definitely discontinued. The investigator will ensure a gradual dose reduction of Escitalopram over at least one or two weeks because of potential symptoms associated with abrupt discontinuation of Escitalopram. Patients with non-response or non-remission will be offered at least one additional clinical appointment at the psychiatric outpatient clinic of the study center after termination of the individual study participation for the gradual discontinuation of Escitalopram.

In the case of premature termination, the reason for withdrawal must be entered on the appropriate case report form (eCRF) page and the patient must be followed for safety and efficacy until the end of the study (week 12).

20.3 Zusammenfassende Nutzen-Risiko-Abwägung

See 10. Risk-benefit-assessment

20.4 Ergänzende Informationen zum Prüfpräparat

See 4.2 Data on IMP-Application

UNIVERSITÄTSMEDIZIN GÖTTINGEN UMG

Statistical Analysis Plan

Version: 1.0 Date: 24/07/2024

Simvastatin add-on to Escitalopram in patients with comorbid obesity and major depression: A multicenter, randomized double-blind, placebo-controlled trial

SIMCODE

**Study Protocol**	Version 1.4, Date 24/09/2021
**EudraCT No.**	2018-002947-27
**Clinicaltrials.gov-No.**	NCT04301271
**Internal Protocol ID No.**	Internal protocol number at the CTU-UMG
**NCT-No.**	NCT04301271
**SAP Version**	Version 1.0, Date 24/07/2024
**SAP Revisions**	*[SAP revision history, Timing of SAP revision]*
**Development Phase**	Phase II
**Sponsor**	Charité - Universitätsmedizin Berlin<br>Charitéplatz 1<br>10117 Berlin
**Coordinating Investigator**	Prof. Dr. Christian Otte<br>Charité - Universitätsmedizin Berlin, Klinik für Psychiatrie und Psychotherapie<br>Campus Benjamin Franklin, Hindenburgdamm 30<br>12203 Berlin

Approval of the Statistical Analysis Plan

**Simvastatin add-on to Escitalopram in patients with comorbid obesity and major depression: A multicenter, randomized double-blind, placebo-controlled trial**	
**EudraCT No.:**	2018-002947-27
**Protocol Version No:**	Version 1.4, Date 24/09/2021

Coordinating Investigator
Prof. Dr. Christian Otte

Date	Signature

Biostatistician

Prof. Dr. Tim Friede

Date	Signature

Table of Contents

Statistical Analysis Plan 1
Approval of the Statistical Analysis Plan 2
Table of Contents 3
List of Figures 4
List of Tables 4
List of Abbreviations 5
1 Introduction 6
1.1 Background and Rationale 6
1.2 Objectives and Endpoints 6
1.3 Explorative objectives and endpoints 7
2 Study methods 8
2.1 Trial design 8
2.2 Randomization 8
2.3 Sample Size 8
2.4 Framework 8
2.5 Statistical Interim Analyses and Stopping Guidance 8
2.6 Timing of the Final Analysis 9
2.7 Timing of Outcome Assessments 9
3 Statistical Principles 10
3.1 Confidence intervals and p-values 10
3.2 Adherence and protocol deviations 10
3.3 Analysis populations 10
4 Trial population 11
4.1 Screening data 11
4.2 Eligibility 11
4.3 Recruitment 13
4.4 Withdrawal/follow-up 13
4.5 Baseline patient characteristics 14
5 Analysis 14
5.1 eCRF Forms 14
5.2 Outcome definitions 15
5.3 Analysis methods 16
5.4 Missing data 17
5.5 Additional analyses 18
5.6 Safety endpoints 18
5.7 Subgroup analyses 18
5.8 Statistical software 19
5.9 References 19

List of Figures

No table of figures entries found.

List of Tables

Table 1 Objectives and related endpoints 7
Table 2 Visit schedule 9

List of Abbreviations

AE	Adverse Event
BDI	Beck Depression Inventory
CGI	Clinical Global Impression Scale
CI	Confidence interval
HDL	high-density lipoprotein
ITT	intention-to-treat
IQR	inter-quartile range
LDL	low-density lipoprotein
MADRS	Montgomery–Åsberg Depression Rating Scale
MCID	Minimally clinically important difference
MDD	Major depressive disorder
MMRM	mixed-model repeated measures
PGIC	Patients’ Global Impression of Change Scale
SOFAS	Social and Occupational Functioning Assessment Scale
WHO	World Health Organization

1 Introduction

This document has been written based on the study protocol version 1.4 dated 24 September 2021. A design paper was published, Otte *et al,* 2022 [1].

1.1 Background and Rationale

Major depressive disorder (MDD) and obesity are major contributors to impaired health worldwide. Statins are among the most prescribed medications with well-established safety and efficacy. Statins are recommended in primary prevention of cardiovascular disease, which has been linked to both MDD and obesity. Moreover, statins are promising candidates to treat MDD because a meta-analysis of pilot randomized controlled trials has found antidepressive effects of statins as adjunct therapy to antidepressants. However, no study so far has tested the antidepressive potential of statins in patients with MDD and comorbid obesity.

1.2 Objectives and Endpoints

Primary objective and endpoint

To examine whether add-on 40 mg/d Simvastatin to standard antidepressant medication (Escitalopram 20 mg/d) improves depression to a greater extent than add-on placebo to standard antidepressant medication (Escitalopram 20 mg/d) in patients with major depression and comorbid obesity. As primary outcome variable the change in Montgomery-Åsberg Depression Rating Scale (MADRS) [2] score from baseline to week 12 was used.

Secondary objectives and endpoints

To examine whether add-on 40 mg/d Simvastatin to standard antidepressant medication (Escitalopram 20 mg/d) improves response rates, remission rates, patients' impression of change, clinicians impression of severity and change, quality of life, social functioning, self-report depression, lipid values, and immunometabolism / mitochondrial function to a greater extent than adjunct placebo in patients with major depression and comorbid obesity.

As secondary outcome variables the following will be used

- MADRS response (defined as 50% MADRS score reduction from baseline), and
- MADRS remission (defined as MADRS score < 10).

Also, as the following secondary outcome variables are assessed

- change scores in patients' self-reported Beck Depression Inventory (BDI-II) as well as
- the MCID according to BDI [4], i.e. 17.5%.

The MCID concept originated from studies of patient-reported outcomes, in which it represented the smallest improvement that was considered meaningful by the patient [3].

Table 1 Objectives and related endpoints

	Objective	Endpoint
**Primary**	Assessment of **depressive symptoms** after intervention	Change score from baseline to week 12 in Montgomery-Asberg-Depression Rating Scale (MADRS)
**Key secondary**	Assessment of **depressive symptoms** after intervention	Change scores in patients' self-reported Beck Depression Inventory (BDI-II)
**Secondary**	Improvement of response and remission rates	MADRS-response (50 % MADRS score reduction from baseline),<br>MADRS-remission (MADRS score < 10)
	Assessment of **depressive symptoms** after intervention	MCID according to BDI (> 17.5% change from baseline)
**Exploratory**	Clinician's impression of the severity of illness	Clinical Global Impression scale – Severity of illness, CGI-S: Change from baseline
	Clinician's impression whether and to what extent symptoms have improved	Clinical Global Impression scale – Improvement, CGI-I: Response defined as "much improved" or "very much improved" post-treatment
	Patient's impression whether and to what extent symptoms have improved	Patients' Global Impression of Change Scale, PGIC: Response defined as „much improved" or „very much improved" post-treatment
	Assessment of **social functioning**	Social and Occupational Functioning Assessment Scale, SOFAS: Change from baseline
	Assessment of **quality of life**	EuroQol-5 Dimensions-3 Levels Questionnaire: Change from baseline and calculated MCID (change from baseline score > 0.74)
	Assessment of changes in **laboratory parameters**	High-density lipoprotein (HDL), low-density lipoprotein (LDL), and total cholesterol: Change from baseline

1.3 Exploratory objectives and endpoints

Additionally the following exploratory objectives and endpoints were/ will be determined

- the clinician's impression of the severity of illness (Clinical Global Impression scale – Severity of illness, CGI-S) [5],
- the clinician's impression whether and to what extent symptoms have improved (Clinical Global Impression scale – Improvement, CGI-I) [5],
- the patient's impression whether and to what extent symptoms have improved (Patients' Global Impression of Change Scale, PGIC),

- social functioning (Social and Occupational Functioning Assessment Scale, SOFAS),
- quality of life (EuroQol-5 Dimensions-3 Levels Questionnaire, with a calculated MCID [4]),
- change scores in high-density lipoprotein (HDL), low-density lipoprotein (LDL), and total cholesterol,
- change scores in mitochondrial function (spare respiratory capacity) of immune cells and indicators of immune function. The evaluation of this endpoint will be performed separately in a dedicated analysis.

2 Study methods

2.1 Trial design

The study was designed as a multi-centre, randomised, double-blind, placebo-controlled, parallel group trial (Phase II).

2.2 Randomization

Patients were randomized in a 1:1 ratio to either intervention (Simvastatin) or control (placebo) in addition to standard antidepressant medication (Escitalopram 20 mg/d). Block-randomization was performed with random block length, stratified for center. Based on the randomization codes, the pharmacy centrally provided for each center sequentially numbered, tamper-proof containers, which were equal in weight and similar in appearance containing IMP or placebo.

2.3 Sample Size

Sample size estimation was done using a literature-based meta-analysis, cf. the study protocol. The mean difference is expected to be of 3−4 points at a standard deviation of 6−8 points. A standardised effect size of 0.5 is considered to be clinically relevant and used to derive the sample size. A sample size of 64 patients per group was determined to be sufficient to achieve a power of 80% for a comparison of the mean changes in MADRS score from baseline to week 12 between the two treatment groups using a two-sample t-test at the usual two-sided level of 5%. Accounting for about 20% dropout, we aim to recruit 80 patients per group, i.e. 160 patients in total. Based on sex-specific prevalence rates and recruitment in our earlier studies [6, 7], we expect that about two thirds of the sample will be women. This will allow sex-specific analyses.

2.4 Framework

Unless stated otherwise, all endpoints will be tested for superiority of the intervention (Simvastatin) over control (placebo).

2.5 Statistical Interim Analyses and Stopping Guidance

No interim analyses are planned.

2.6 Timing of the Final Analysis

The final analysis will take place when all outcomes have been collected, a blinded review of the data was performed and the database is locked.

2.7 Timing of Outcome Assessments

The final analysis will take place when all outcomes have been collected and the database is locked. Table 2 displays the assessment of variables within visit time points.

Table 2 Visit schedule

	SCREENING	BASELINE/VISIT 1	VISIT 2	VISIT 3	VISIT 4	VISIT 5	VISIT 6
		V1	V2	V3	V4	V5	V6
**Week**	-1	0	1	2	4	8	12
Eligibility screen		X					
Randomisation		X					
Anamnesis	X						
Physical data, ECG	X	X	X	X	X	X	X
Physical Examination	X						X
Internal medical status							X
Lab values	X	X			X	X	X
MADRS	X	X	X	X	X	X	X
M.I.N.I.	X						
BDI-II		X	X	X	X	X	X
SOFAS, EQ-5D		X			X		X
PGIC			X	X	X	X	X
CGI		X	X	X	X	X	X
Study medication							X
Exclusion criteria			X	X	X	X	X

3 Statistical Principles

3.1 Confidence intervals and p-values

All hypothesis tests will be performed at a two-sided significance level of 5% or a one-sided level of 2.5%. Confidence intervals (CI) will be reported with two-sided 95% confidence level.

P-values ≥ 0.0001 will be reported to 4 decimal places; p-values less than 0.0001 will be reported as “<0.0001”. The mean, standard deviation, and any other statistics other than quantiles, will be reported to one decimal place greater than the original data. Quantiles, such as median, or minimum and maximum will use the same number of decimal places as the original data. Estimated parameters, not on the same scale as raw observations (e.g. regression coefficients) will be reported to 3 significant figures, and will be accompanied by 95% confidence intervals whenever possible.

3.2 Adherence and protocol deviations

All efforts were made and measures taken to collect complete outcome data from randomization to the end-of-study visit for each subject randomized in this study regardless of compliance.

Numbers of non-compliant patients, early discontinuations including withdrawal, lost to follow-up patients will be summarized by frequencies and percentages. Treatment compliance was defined as taking at least 90% of the treatment medication which is assessed as the difference between the number of given and returned pills. For patients who did not return the treatment packaging treatment compliance could not be assessed and was considered as missing value. We perform a sensitivity analysis where these patients are considered as non-compliant.

3.3 Analysis populations

The efficacy analyses will be based on the **full analysis set (FAS)** following the intention-to-treat (ITT) principle and will include all correctly randomized patients with at least one post-baseline documented data point of the primary endpoint.

Safety analyses will be based on the **safety set (SAF)** including all randomized patients according to the treatment received considering also the timing of intervention.

Sensitivity analysis will be performed on all patients completing the study adherent to the study protocol. This will be referred to as the per-protocol (PP) population. Moreover, we will perform an analysis on the set of all participants completing all assessments (complete case analysis).

4 Trial population

4.1 Screening data

Screening took place within the participating centres. Available data on patients assessed for eligibility will be included within a CONSORT flow diagram.

4.2 Eligibility

Details are provided in the study protocol. In brief, patients had to fulfill the following inclusion criteria and had not to fulfill any of the exclusion criteria listed below at the same time.

Inclusion criteria:

- Written informed consent is present
- The patient has the capacity to give consent (He/she is able to understand the nature and anticipated effects/side effects of the proposed medical intervention)
- The patient has a major depressive episode according to DSM 5 (Diagnostic and Statistical Manual of Mental Disorders 5th Edition)
- The patient has a score of ≥ 18 in the Montgomery-Asberg Depression Rating Scale (MADRS)
- The patient has a body mass index ≥ 30
- The patient's age is between 18 and 65 years (≥ 18 und ≤ 65)
- The patient has not given childbirth within the 6 months prior to study entry and is not breastfeeding
- In case of non-psychotropic medication: The patient received stable pharmacological medication for at least 14 days prior to study entry (any changes in medication dose or frequency of therapy must be answered with no)
- The patient did not take antidepressants during the last 7 days prior to study entry (discontinuation of effective medication to enable study participation is prohibited)
- The patient did not receive prior treatment with Escitalopram in index episode
- The patient had less than three (<3) trials with antidepressants in index episode
- The patient does not have a history of non-response to Escitalopram
- The patient did not receive treatment with ketamine, electroconvulsive therapy (ECT) or other stimulatory treatments in index episode
- The patient does not meet any of the following criteria:
  - Schizophrenia
  - Schizoaffective disorder
  - Bipolar disorder
- The patient is not diagnosed with dementia and does not have moderate or severe impairment of general cognitive function according to clinical impression

- The patient does not have clinically relevant elevated liver enzymes [GOT or GPT > 3 x upper limit normal (ULN)] and does not have elevated Carbohydrate Deficient Transferrin (CDT) ≥ 2.4 %
- The patient does not meet the criteria for alcohol use disorder (DSM-5: 303.90; ICD-10: F10.20) or substance use disorder (DSM-5: 304; ICD-10: F11.20 – F19.20) in M.I.N.I. for DSM-5 and a urine/serum drug screening is negative (except for benzodiazepines and opiates)
- The patient does not have a history of suicide attempt
- The patient does not have diagnosed epilepsy or increased bleeding diathesis or a history of angle closure glaucoma or other glaucomas
- The patient did not have bariatric surgery prior to study entry
- The patient does not have a known allergy or contraindication against Escitalopram or Simvastatin
- The patient does not meet any of the following criteria:
  - hereditary muscle disease
  - known history of rhabdomyolysis
  - elevated creatine kinase (CK) outside of the sex-specific reference intervals
  - History of muscular symptoms under treatment with statins or fibrates
- The patient does not have elevated TSH level outside of the age- and sex-specific reference intervals.
- The patient does not have insulin-dependent diabetes mellitus
- The patient does not have uncontrolled hepatic disorder, renal or cardiovascular disease
- The patient does not have untreated hypothyroidism
- The patient does not have a history of myocardial infarction or stroke
- The patient does not have symptomatic peripheral arterial disease
- The patient does not have monogenic familial hypercholesterolemia
- The patient does not have clinically significant laboratory abnormalities
- The patient did not participate in other interventional trials during the 6 months before and at the time of this trial
- The patient is not an employee of the investigator study site, or a family member of the employees or the investigator, or otherwise dependent on the sponsor, the investigator or the investigator study site.

Exclusion criteria:

- The patient has current use of statins (for visits 2-6 applies: except for IMP Simvastatin)
- The patient has current use of antidepressants (for visits 2-6 applies: except for standard medication Escitalopram)
- The patient has acute suicidal tendencies (MADRS Item 10 > 4)

- The patient uses potent CYP3A4-inhibitors (e.g. clarithromycin, erythromycin, HIV protease inhibitors – see "Risks, adverse drug reactions, drug interactions, restrictions, contraindications, procedures in case of emergency")
- The patient uses potent CYP3A4 inductors (carbamazepine, efavirenz, nevirapin, etravirine).
- The patient uses Fibrates, Amiodaron, Amlodipin, Verapamil, Fluconazol, Diltiazem, Fusidic acid, Niacin, Daptomycin or Lomitapid or BCRP-Inhibtors (e.g. Elbasvir or Grazoprevir)
- The patient uses Gemfibrozil, Ciclosporin or Danazol
- The patient has known hypersensitivity to other ingredients of Simvastatin and Escitalopram [butylated hydroxyanisole, microcrystalline celluose, citric acid, starch, lactose, magnesium stearate, hypromellose, talc, titanium dioxide, iron oxies, colloidal silicon dioxide, croscarmellose sodium, polyethylene glycol]
- The patient uses medication that is associated with QTc-prolongation [antiarrhythmica class IA and III, antipsychotics (e.g. haloperidol), phenothiazines, tricyclic antidepressants, antibiotics (e.g. moxifloxacin), and certain antihistaminergic drugs (e.g. astemizol, mizolastin)]
- The patient has clinically significant abnormalities in 12-lead ECG (e.g. QTc-prolongation ≥ 500 ms or increase ≥ 60 ms from baseline visit)
- The patient is pregnant
- The patient with childbearing potential is not willing to use an acceptable form of contraception (defined as Pearl index < 1)
- The patient has current use of psychotropic medication (e.g. antipsychotics, anticonvulsants, lithium or St. John's Wort) except for benzodiazepines, non-benzodiazepines and opiates
- The patient uses nonselective, irreversible monoamine oxidase (MAO) inhibitor (e.g. Tranylcypromine) or selective, reversible inhibitor of monoamine oxidase A (e.g. Moclobemide) or the nonselective, reversible monoamine oxidase inhibitor Linezolid
- The patient is unwilling to consent to saving, processing and propagation of pseudonymized medical data for study reasons
- The patient is legally detained in an official institution
- The patient has active SARS-CoV-2 infection

4.3 Recruitment

Recruitment numbers will be reported and described within a CONSORT flow diagram.

4.4 Withdrawal/follow-up

Participants were allowed to leave the study at any time or withdraw consent. Reasons for study withdrawal will be documented using a CONSORT flowchart.

4.5 Baseline patient characteristics

All baseline patient characteristics will be summarized using descriptive statistics (e.g. mean, standard deviation, median, interquartile range (IQR) for continuous variables and frequencies (percentages) for categorical variables) and appropriate graphical methods (e.g. boxplots, histograms, barplots) depending on the data type.

5 Analysis

5.1 eCRF Forms

Details are provided in the study protocol; see Section 15.

5.2 Outcome defintions

A full list of outcomes and their timing is described in Section 2.7. For a detailed description of all outcomes see study protocol Section 5.

Each patient had to undergo a **physical examination** where several body systems were examined. **Vital signs** such as blood pressure or heart rate were assessed and blood samples were taken which were analysed in a **laboratory**. Moreover, at each visit an **electrocardiogram** was obtained. For the clinical parameters acquired during this procedure we refer to the study protocol.

MADRS is a ten-item questionnaire with a 7-points Likert scale from 0 to 6. The score is the sum of all items and ranges from 0 to 60 [2].

Depressive symptoms are measured with the **Mini-International Neuropsychiatric Interview (M.I.N.I.)**. M.I.N.I. is a short structured diagnostic interview for the major Axis I psychiatric disorders in DSM-IV and ICD-10 [8]. The number of patients with a clinical diagnosis of current major depressive disorder according to M.I.N.I. will be analysed.

Beck depression inventory (BDI-II) [9] is a 21-question self-reported inventory for measuring the severity of depression. Each question has a set of at least four possible responses, ranging in intensity. To calculate the score a value of 0 to 3 is assigned for each answer. The total score is the sum of all item values. A higher total score indicates more severe depressive symptoms. The interpretation of these scores is:

- 0-13: minimal depression,
- 14-19: mild depression,
- 20-28: moderate depression,
- 29-63: severe depression.

The **EQ-5D-3L** descriptive system comprises the following five dimensions: mobility, self-care, usual activities, pain/discomfort and anxiety/depression. Each dimension has 3 levels: no problems, some problems, and extreme problems. The patient is asked to indicate his/her health state by ticking the box next to the most appropriate statement in each of the five dimensions. This decision results into a 1-digit number that expresses the level selected for that dimension. The digits for the five dimensions can be combined into a 5-digit number that describes the patient's health state.

The **EQ VAS** records the patient's self-rated health on a vertical visual analogue scale from 0 to 100 where the endpoints are labelled 'Worst imaginable health state' and 'Best imaginable health state'. The VAS can be used as a quantitative measure of health outcome that reflects the patient's own judgement.

The **Patient Global Impression of Change (PGI-C)** measures the patient's subjective improvement [10] on a 7-point Likert scale ranging from 1 (a lot better) to 7 (a lot worse).

The **Social and Occupational Functioning Assessment Scale (SOFAS)** will be used to determine social functioning [11]. It is assessed on a numerical scale raning from 0 (inadequate information) over 10 (persistent inability to maintain minimal personal hygiene) and 50 (serious impairment in social, occupational or school functioning) to 100 (superior functioning in a wide range of activities).

The **Clinical Global Impression scale (CGI)** consists of 2 sub-scales: CGI-S and CGI-I. Clinical Global Impression scale – Severity of illness (CGI-S) and Clinical Global Impression scale - Improvement (CGI-I) will be used to assess patient's overall severity and improvement/worsening of depression [5]. CGI-S is assessed on an 8-point Likert-scale from 0 (not assessable) and 1 (patient is not at all ill) to 7 (patient is one of the extremely seriously ill). CGI-I is assessed on an 8-point Likert-scale from 0 (not assessable) and 1 (state is a lot better) to 7 (state is a lot worse).

5.3 Analysis methods

Primary endpoint

The primary analysis will compare MADRS changes from baseline to week 12 between the intervention (Simvastatin) and control (placebo) group by Gaussian linear models for repeated measures (so-called MMRM) with intervention group, center, time (week 1, 2, 4, 8, and 12), and intervention-by-time interaction as factors, and baseline MADRS score as covariate. The error terms are assumed to follow a multivariate normal distribution with unstructured covariance. Least squares mean changes from baseline will be reported for both groups with 95% confidence interval (CI) as well as the difference between the least squares treatment group means with 95%-CI and p-value testing the null hypothesis of no treatment effect. Degrees of freedom are approximated using the Kenward-Roger method.

```
library(mmrm)
library(emmeans)

model <- mmrm(madrs_change~ intervention + centre + time + intervention * time +
                   madrs_baseline + us(time | id), data = data)

res <- car::Anova(model, type = "3", test.statistic = "F")
res

# report least square group differences stratified by time
tmt_means <- emmeans(model, specs = "intervention", by = "time")
pairs(tmt_means)
```

Secondary endpoints

If the primary endpoint is statistically significant, the key secondary endpoint will be tested in a confirmatory fashion. If the key secondary endpoint is statistically significant, further secondary endpoints will be tested in a confirmatory fashion by application of the Hochberg procedure [12]. The familywise type I error rate will be controlled using hierarchically order hypotheses and the Hochberg procedure. Analyses of exploratory and safety endpoints have an exploratory character and will therefore not be adjusted for multiple testing.

The secondary efficacy endpoints MADRS-response and MADRS-remissionwithin 12 weeks will be analyzed by logistic regression models with intervention and center as factors and baseline MADRS as covariate. The treatment effects will be reported as odds ratios with 95%-CI and p-values testing the null hypotheses of no treatment effect. The analyses of continuous secondary efficacy endpoint as well as SOFAS, and EQ-5D-3L will follow the same lines as the primary outcome.

We will repeat the analyses in the per-protocol set and the complete case set.

```
library(emmeans)

model <- glm(madrs_response~ intervention + centre + madrs_baseline, data = data,
             family = binomial)

# report least square group differences stratified by time
tmt_means <- emmeans(model, specs = "intervention", type = "response")

summary(tmt_means)
pairs(tmt_means)
```

Exploratory endpoints

Analyses of exploratory endpoints have an exploratory character and will therefore not be adjusted for multiple testing.

Exploratory variables will be summarized using descriptive statistics (e.g. mean, standard deviation, median, interquartile range for continuous variables and frequencies (percentages) for categorical variables) and appropriate graphical methods (e.g. boxplots, histograms, barplots) depending on the data type.

5.4 Missing data

Although the MMRM approach described above is robust to a certain extend to missing data, sensitivity analyses will be performed as supporting analyses including multiple imputation and last-observation-carried-forward (LOCF). The latter will also facilitate comparison with previous trials.

Multiple imputation

Endpoints will be imputed multiply using predictive mean matching [13, Section 3.4] based on patient‘s study arm, centre and baseline value. The number of imputed datasets was set to m = 10.

```
library(mice)
library(dplyr)
library(emmeans)

# multiple imputation
imp <- mice(data, m = n_imputations, seed = 42)

models_mi <- with(data = imp, exp = glm(madrs_response~ intervention + centre +
                                        madrs_baseline, family = binomial))

emmeans(models_mi, specs = "intervention", type = "response") %>%
  pairs() %>%
  confint()
```

LOCF

Additionally, imputation by last-observatio-carried-forward analyses will be performed for all post-randomisation assessments. In case of no available baseline assessment, corresponding patients will be excluded from the analyses.

```
library(dplyr)
library(emmeans)
library(zoo)

# last observation carried forward
data_locf <- data_prim %>%
  group_by(id) %>%
  mutate_at("madrs_response", ~ na.locf(., na.rm = FALSE))

model_locf <- glm(madrs_response~ intervention + centre + madrs_baseline,  data =
                  data_locf, family = binomial))

emmeans(model_locf, specs = "intervention", type = "response") %>%
  pairs() %>%
  confint()
```

5.5 Additional analyses

Blinding check: The degree of blinding successfulness will be measured using Cohen‘s kappa (κ) as proposed in [14]. A κ value of 0 indicated successful blinding, and a k value of 1 reveals that all the patients/ assessors could correctly identify patients’ treatment so that the blinding was totally broken. Moreover, we will test the estimated probability of guessing the treatment arm correctly being 50% using a Chi-Square-Test. The “don’t know” response will be assigned proportionally by the number of active-treatment and control responses.

Mitochondrial function: The change from baseline in mitochondrial function (spare respiratory capacity) of immune cells and indicators of immune function will be assessed in an ancillary substudy. Further details regarding the evaluation will be specified in a dedicated document.

5.6 Safety endpoints

All adverse events (AE; any undesirable experience occurring to a participant during the trial, whether or not considered related to the study medication) reported by the subject or observed by the investigators were recorded at each visit. Standard procedures for reporting of (serious) adverse events were used. Additionally, for event types muscle pain and increase in depression (i.e. decrease in MADRS from baseline to study end of at least 1.9 points) logistic regression models will be fitted and study groups will be compared using Chi-Square-Tests.

(Serious) adverse events will be summarized as frequencies and percentages by treatment group. For recurrent events and to account for variable follow-up times, event rates will be reported with rate ratios comparing Simvastatin with add-on placebo control and 95% confidence intervals. We will use Poisson regression models with adjustment for over dispersion (or negative binomial regression models) with offset for follow-up time and possibly a mixture component to account for zero-inflation.

Analyses of safety endpoints have an exploratory character and will therefore not be adjusted for multiple testing.

5.7 Subgroup analyses

Subgroup analyses concerning the primary endpoints are planned to explore a possible heterogeneity of the treatment effect concerning the following baseline characteristics:

- Sex [male/ female]
- Age [years]
- Severity of obesity [30 − <35, ≥35 − <40, ≥40]
- Metabolic syndrome [yes/ no]
- Severity of depression with respect to MADRS [15] [moderate (≥18 − <34)/ severe (≥34)]
- Inflammatory status [CRP > 3/ ≤ 3]
- Chronicity of depression [yes/ no]

To this end, mixed linear models will be fitted for the primary endpoint with the above mentioned covariates as fixed effects and random individual effect to account for repeated measures. We will report expected marginal means and pairwise contrasts, respectively.

5.8 Statistical software

Statistical analysis will be performed using R version 4.3.3 or higher or SAS version 9.4 or higher. Employed R packages and the specific version will be documented within the statistical report. Multiple imputation will be conducted using the R package *mice*. For last-observation-carried-forward imputation, we will employ the R package *zoo*.

5.9 References

1. Otte, C., Chae, W. R., Nowacki, J., Kaczmarczyk, M., Piber, D., Roepke, S., ... & Gold, S. M. (2020). Simvastatin add-on to escitalopram in patients with comorbid obesity and major depression (SIMCODE): study protocol of a multicentre, randomised, double-blind, placebo-controlled trial. BMJ open, 10(12), e040119.

2. Montgomery, SA (1979). Åsberg M. *A new depression scale designed to be sensitive to change. Br J Psychiatry*, *134*, 382-389.

3. Brozek, J.L., G.H. Guyatt, and H.J. Schunemann, How a well-grounded minimal important difference can enhance transparency of labelling claims and improve interpretation of a patient reported outcome measure. Health Qual Life Outcomes, 2006.4: p. 69.

4. Button, K.S., et al., Minimal clinically important difference on the Beck Depression Inventory--II according to the patient's perspective. Psychol Med, 2015. 45(15): p. 3269-79.

5. Guy, W., ECDEU Assessment Manual for Psychopharmacology. 1976, Rockville:National Institue of Mental Health.

6. Otte, C., et al., Modulation of the mineralocorticoid receptor as add-on treatment in depression: a randomized, double-blind, placebo-controlled proof-of-concept study. Journal of Psychiatric Research, 2010. 44(339-46).

7. Otte, C., et al., Mineralocorticoid receptor stimulation improves cognitive function and decreases cortisol secretion in depressed patients and healthy individuals. Neuropsychopharmacology : official publication of the American College of Neuropsychopharmacology, 2015. 40(2): p. 386-93.

8. Sheehan, D. V., Lecrubier, Y., Sheehan, K. H., Amorim, P., Janavs, J., Weiller, E., Hergueta, T., Baker, R., & Dunbar, G. C. (1998). The Mini-International Neuropsychiatric Interview (M.I.N.I): The development and validation of a structured diagnostic psychiatric interview for DSM-IV and ICD-10. *The Journal of Clinical Psychiatry, 59*(Suppl 20), 22–33.

9. Beck, A. T., Steer, R. A., & Brown, G. K. (1996). *Manual for the Beck depression inventory-II*. San Antonio, TX: Psychological Corporation.

10. Guy, W., ECDEU Assessment Manual for Psychopharmacology. 1976, Rockville, MD: US Department of Health, Education, and Welfare Public Health Service Alcohol, Drug Abuse, and Mental Health Administration.

11. Morosini, P.L., et al., Development, reliability and acceptability of a new version of the DSM-IV Social and Occupational Functioning Assessment Scale (SOFAS) to assess routine social functioning. Acta Psychiatr Scand, 2000. 101(4): p. 323-9.

12. Hochberg, Y. (1988). A sharper Bonferroni procedure for multiple tests of significance. Biometrika, 75(4), 800-802.

13. Van Buuren, S. (2018). Flexible imputation of missing data. CRC press.

14. Lin, Y. H., Sahker, E., Shinohara, K., Horinouchi, N., Ito, M., Lelliott, M., ... & Furukawa, T. A. (2022). Assessment of blinding in randomized controlled trials of antidepressants for depressive disorders 2000–2020: A systematic review and meta-analysis. EClinicalMedicine, 50.

15. Williams, J. B., & Kobak, K. A. (2008). Development and reliability of a structured interview guide for the Montgomery-Åsberg Depression Rating Scale (SIGMA). The British Journal of Psychiatry, 192(1), 52-58.
