# Supplementary material for: Simvastatin as Add-On Treatment to Escitalopram in Patients With Major Depression and Obesity: A Randomized Clinical Trial
Source: JAMA Psychiatry. 2025 Jun 4;82(8):759–67. doi: 10.1001/jamapsychiatry.2025.0801 (PMC12138799; doi:10.1001/jamapsychiatry.2025.0801)
Supplement: Supplement 2. — eMethods. eTable 1. Recruitment by Site and Group Allocation eTable 2. Pill Count Overall and by Treatment Group eTable 3. Escitalopram Dosage at End of Study (week 12) Overall and by Treatment Group eTable 4. Concurrent Medication Given as Proportions of the Population Taking at Least 1 Medication From the Corresponding Categories eTable 5. Baseline and Posttreatment (Week 12) Values for Primary, Secondary, and Exploratory Outcomes eTable 6. (Serious) Adverse Events Overall and by Treatment Group eTable 7. Change in Body Weight and Body Mass Index Overall and by Treatment Group eFigure 1. PRISMA Flow Diagram eFigure 2. Probability for MADRS Response and Remission After 12 Weeks With Corresponding 95% CIs eFigure 3. Prevalence of LDL, Total Cholesterol, and CRP Over a Predefined Cutoff With Corresponding 95% CIs, Stratified by Visit and Treatment Group eFigure 4. Subgroup Analyses eFigure 5. Suicidal Symptoms, Assessed by MADRS Item 10, at Screening, Baseline, and Throughout the Study by Treatment Group eFigure 6. Meta-Analysis of All Available Evidence From RCTs Evaluating the Efficacy of Statins for Depression Severity in Adults With MDD eReferences. [file jamapsychiatry-e250801-s002.pdf]

Supplementary Online Content

Otte C, Chae WR, Dogan DY, et al. Simvastatin as add-on treatment to escitalopram in patients with major depression and obesity: a randomized clinical trial. *JAMA Psychiatry*. Published online June 4, 2025. doi:10.1001/jamapsychiatry.2025.0801

eMethods.

eTable 1. Recruitment by Site and Group Allocation

eTable 2. Pill Count Overall and by Treatment Group

eTable 3. Escitalopram Dosage at End of Study (week 12) Overall and by Treatment Group

eTable 4. Concurrent Medication Given as Proportions of the Population Taking at Least 1 Medication From the Corresponding Categories

eTable 5. Baseline and Posttreatment (Week 12) Values for Primary, Secondary, and Exploratory Outcomes

eTable 6. (Serious) Adverse Events Overall and by Treatment Group

eTable 7. Change in Body Weight and Body Mass Index Overall and by Treatment Group

eFigure 1. PRISMA Flow Diagram

eFigure 2. Probability for MADRS Response and Remission After 12 Weeks With Corresponding 95% CIs

eFigure 3. Prevalence of LDL, Total Cholesterol, and CRP Over a Predefined Cutoff With Corresponding 95% CIs, Stratified by Visit and Treatment Group

eFigure 4. Subgroup Analyses

eFigure 5. Suicidal Symptoms, Assessed by MADRS Item 10, at Screening, Baseline, and Throughout the Study by Treatment Group

eFigure 6. Meta-Analysis of All Available Evidence From RCTs Evaluating the Efficacy of Statins for Depression Severity in Adults With MDD

eReferences.

This supplementary material has been provided by the authors to give readers additional information about their work.

© 2025 Otte C et al. *JAMA Psychiatry*.

eMethods

Inclusion and exclusion criteria

The main inclusion criteria for the trial were: (i) Written informed consent; (ii) Major depressive episode according to DSM-5 (Diagnostic and Statistical Manual of Mental Disorders 5th Edition); (iii) Montgomery-Åsberg Depression Rating Scale (MADRS) score ≥ 18; (iv) BMI ≥ 30 kg/m2; (v) Age between 18 and 65 years (≥ 18 and ≤ 65); (vi) non-psychotropic medication had to be kept stable for at least 14 days prior to study entry; (vii) No antidepressant intake during the last 7 days prior to study entry (discontinuation of effective medication to enable study participation was prohibited); (viii) No prior treatment with escitalopram during the index episode; (ix) Less than three trials with antidepressants during the index episode; (x) No treatment with ketamine, electroconvulsive therapy or other stimulatory treatments such as repetitive transcranial magnetic stimulation (rTMS) or vagus nerve stimulation during the index episode; (xi) None of the following disorders: schizophrenia, schizoaffective disorder, bipolar disorder. In addition, patients with any severe, unstable general medical condition or contraindications for simvastatin or escitalopram were not included in the study.

Main exclusion criteria were: (i) Current use of statins; (ii) Current use of antidepressants; (iii) Acute suicidal ideation (MADRS Item 10 > 4); (iv) Pregnancy, breastfeeding or women with childbearing potential without acceptable form of contraception (defined as Pearl index < 1); (v) Current use of psychotropic medication (e.g. antipsychotics, anticonvulsants, lithium or St. John's wort) except for benzodiazepines, non-benzodiazepines and opiates (vi) Clinically significant abnormalities in 12-lead electrocardiogram (ECG; e.g. QTc-prolongation ≥ 500 ms or increase ≥ 60 ms from baseline visit). In addition, there were several other exclusion criteria including medication that is contraindicated with simvastatin or escitalopram. Complete inclusion and exclusion criteria are provided in the Trial Protocol (supplementary material).

Exploratory outcomes

Exploratory outcomes included changes from baseline in the Clinical Global Impression Scale for Severity (CGI-S) and Improvement (CGI-I) (36), the Patient Global Impression of Change (PGIC) (36), social functioning assessed by the Social and Occupational Functioning Assessment Scale (37), and quality of life measured by the EuroQol-5 Dimensions-3 Levels Questionnaire, with a calculated minimally clinically important difference (MCID) (38). Additional exploratory outcomes included laboratory parameters such as high-density lipoprotein (HDL), low-density lipoprotein (LDL), total cholesterol levels, and high-sensitivity C-reactive protein (hsCRP).

Statistical analysis

The statistical analysis plan (SAP) (see Supplementary Materials) was finalized and signed on July 24, 2024, before unblinding of the data. Statistical analyses were started on August 30, 2024, following database lock.

The error terms were assumed to follow a multivariate normal distribution with unstructured covariance. Least squares mean changes from baseline are reported for both groups with 95% confidence intervals (95%-CI) as well as the difference between the least squares treatment group means with 95%-CI and p-value for testing the null hypothesis of no treatment effect. Degrees of freedom were approximated using the Kenward-Roger method.

Effect sizes are reported as Cohen's d including 95%-CI by calculating the difference in means between the treatment and control groups, divided by the pooled standard deviation, which was computed using the square root of the weighted variances of both groups. The 95%-CI for Cohen's d was derived using standard error estimates based on group sample sizes and variances.

Additional analyses included descriptive statistics (e.g., mean, standard, deviation, median, interquartile range for continuous variables and frequencies for categorical variables) and a blinding check using Cohen's kappa as previously proposed by (39). Moreover, we checked the estimated probability of guessing the treatment arm correctly being 50% using a Chi-Square-Test.

Updated systematic review and meta-analysis

The protocol defined a priori the review question, search strategy, inclusion and exclusion criteria, risk of bias assessment, and data synthesis plan in accordance with the Preferred Reporting Items for Systematic Reviews and Meta-Analyses (PRISMA) protocol statement. The protocol was prospectively registered with PROSPERO (CRD42024588640) on September 10, 2024 (1). A separate review protocol was not prepared. There was no financial or non-financial support for this review.

Search strategy: PubMed and EMBASE were searched on September 10, 2024 using the search terms: "(statin* OR statin) AND depress* AND random*". This approach was based on a recent analysis indicating that searching

© 2025 Otte C et al. *JAMA Psychiatry*.

at least two databases captures approximately 94.1% of relevant articles, with the inclusion of a third database, such as CENTRAL, providing a marginal increase in coverage to 98.5% (2). The search was performed without restrictions on the publication time frame.

Selection criteria: Studies were selected based on criteria according to the following PICOs: **P**: adults with major depressive disorder (MDD); **I**: treatment with a statin (ATC group C10AA HMG CoA reductase inhibitors; any product or dose) as monotherapy or add-on to antidepressants / standard of care; planned treatment for at least 4 weeks; **C**: placebo; **O**: depression severity as measured by a clinician rating scale; **s**: randomized controlled trials.

Screening and data extraction: Titles and abstracts were screened by at least two reviewers independently (WRC, DYD, SMG) and conflicts were resolved by discussion involving an additional reviewer (JB) who had not initially screened the abstract. Then, full texts were examined and screened for applicability based on the predefined inclusion and exclusion criteria by WRC, DYD, and SMG independently. Data extraction and quality assessment using the revised Cochrane risk-of-bias tool for randomized trials (RoB 2) were performed by pairs of independent reviewers (WRC / JB and DYD / SMG, respectively) following PRISMA guidelines. Conflicts were resolved by discussion involving a third author (CO). Data on sample size, statin type and dose, duration of treatment for primary endpoint, demographic variables (sex, age), and depression severity (e.g. HDRS, MADRS) including means and standard deviations, for groups receiving statins or placebo were extracted. Assumptions on missing or unclear information were made using other literature such as published meta-analysis of included studies. Risk of bias was assessed using the revised Cochrane risk-of-bias tool for randomized trials (RoB 2).

Statistical analysis: Random-effects meta-analyses with inverse-variance weighting were conducted on the aggregate data. Due to varying outcome scales across studies, standardized mean differences were used as effect measures. The Knapp-Hartung approach was used for type-I-error. The Paule-Mandel estimator was applied to account for between-study heterogeneity. Between-trial heterogeneity was visually explored in forest plots; 95% prediction intervals are included to indicate the range of expected true study effects. For analyses the R package metafor (4) was employed. All analyses were carried out using R version 4.3.3.

Results: We screened 390 abstracts/titles and identified seven eligible, published RCTs evaluating the efficacy of statins in adults with MDD. Including our present trial, eight trials were thus available for the meta-analysis (see Supplementary Figure 1 for the PRISMA flowchart).

© 2025 Otte C et al. *JAMA Psychiatry*.

Supplementary Tables and Figures

eTable 1: Recruitment by site and group allocation.

Centre	Overall, N = 160	Placebo, N = 79	Treatment, N = 81
**Berlin Charité, Psychiatrie**	62 (39%)	31 (39%)	31 (38%)
**Berlin Charité, Psychosomatik**	22 (14%)	11 (14%)	11 (14%)
**Frankfurt**	7 (4.4%)	4 (5.1%)	3 (3.7%)
**Greifswald**	5 (3.1%)	2 (2.5%)	3 (3.7%)
**Hamburg**	18 (11%)	9 (11%)	9 (11%)
**Hannover**	8 (5.0%)	4 (5.1%)	4 (4.9%)
**Leipzig**	25 (16%)	12 (15%)	13 (16%)
**Lübeck**	4 (2.5%)	2 (2.5%)	2 (2.5%)
**Stralsund**	9 (5.6%)	4 (5.1%)	5 (6.2%)

eTable 2: Pill count overall and by treatment group.

	Overall	Escitalopram + Placebo	Escitalopram + Simvastatin
**Simvastatin/ Placebo medication**			
Mean no. of pills taken (n)	78 ± 14	79 ± 16	78 ± 12
Mean no. of pills taken (%)	80 ± 10	81 ± 9	80 ± 12
**Escitalopram medication**			
Mean no. of pills taken (n)	80 ± 15	82 ± 16	79 ± 15
Mean no. of pills taken (%)	67 ± 15	68 ± 14	66 ± 16

eTable 3: Escitalopram dosage at end of study (week 12) overall and by treatment group.

Dosage	Total	Escitalopram + Placebo	Escitalopram + Simvastatin
**10 mg**	3 (2%)	2 (3%)	1 (1%)
**20 mg**	145 (98%)	73 (97%)	72 (99%)

Escitalopram dosage at the end of the study (week 12) was missing for six participants; therefore, data from only 148 participants are presented here.

© 2025 Otte C et al. *JAMA Psychiatry*.

eTable 4: Concurrent medication given as proportions of the population taking at least one medication from the corresponding categories.

Medication category	Frequency
Antiinflammatory and antirheumatic drugs	25 (29%)
Blood pressure medication	22 (25%)
Hormon preparations	22 (25%)
Other analgetics	19 (22%)
Proton pump inhibitors	10 (11%)
Benzodiazepines/Benzodiazepine-related drugs	7 (8%)
Antidiabetic drug	6 (7%)
Antibiotics	6 (7%)
Antihistamines	6 (7%)
Hypnotics and other sedatives	5 (6%)
Asthma medication	4 (5%)
Antidepressants	2 (2%)
Diuretics	2 (2%)
Others	58 (67%)

*In two patients who discontinued treatment with Escitalopram + Simvastatin/Placebo but continued follow-up until the end of the study, other antidepressants (i.e., Venlafaxine) were used as antidepressant therapy after discontinuation. Consistent with the intention-to-treat principle, these medications have been included as concurrent medications in this table.

eTable 5: Baseline and post treatment (week 12) values for primary, secondary, and exploratory outcomes.

		**Baseline**	**Week 12**
		Mean (SD)	Mean (SD)
**MADRS**			
	Treatment	25.8 (4.8)	12 (8)
	Placebo	25.2 (5.2)	12 (9)
**BDI-II**			
	Treatment	30 (10)	16 (12)
	Placebo	30 (10)	17 (12)
**SOFAS**			
	Treatment	57 (12)	70 (14)
	Placebo	58 (11)	70 (15)
**EQ-5D (VAS)**			
	Treatment	48 (17)	66 (19)
	Placebo	49 (19)	64 (21)
**CGI-S**			
	Treatment	4.75 (0.65)	3.32 (1.22)
	Placebo	4.73 (0.65)	3.32 (1.19)
**HDL**			
	Treatment	49 (11)	49 (13)
	Placebo	50 (13)	50 (14)
**LDL**			
	Treatment	127 (33)	87 (35)
	Placebo	124 (33)	120 (31)
**Total cholesterol**			
	Treatment	197 (38)	159 (46)
	Placebo	195 (39)	191 (40)

© 2025 Otte C et al. *JAMA Psychiatry*.

eTable 6: (Serious) Adverse Events overall and by treatment group.

	Total	Escitalopram + Placebo	Escitalopram + Simvastatin	P-value
**Number of patients with AEs (%)**	123	62 (78%)	61 (75%)	0.634
**Number of patients with SAEs (%)**	2	1 (1%)	1 (1%)	>0.999
**Adverse events**				
**Headache**	39	19	20	
**Nausea**	26	11	15	
**Minor upper respiratory tract infection**	16	9	7	
**Fatigue**	13	6	7	
**Elevated creatine kinase (CK)**	12	7	5	
**Restlessness**	12	6	6	
**Dizziness**	11	2	9	
**Diarrhea**	10	8	2	
**Sleep disturbances**	9	5	4	
**Hyperhidrosis**	9	6	3	
**Delayed ejaculation**	6	1	5	

All adverse events occurring more than five times, per group, are shown.

eTable 7: Change in body weight and body mass index overall and by treatment group.

	Overall	Escitalopram + Placebo	Escitalopram + Simvastatin	p-value
**Change in Weight [kg]**	−0.9 ± 4.1	−0.2 ± 3.9	−0.4 ± 4.3	0.742
**Change in BMI [kg/m²]**	−0.09 ± 1.39	−0.06 ± 1.35	−0.12 ± 1.44	0.800

© 2025 Otte C et al. *JAMA Psychiatry*.

eFigure 1: PRISMA Flow Diagram.

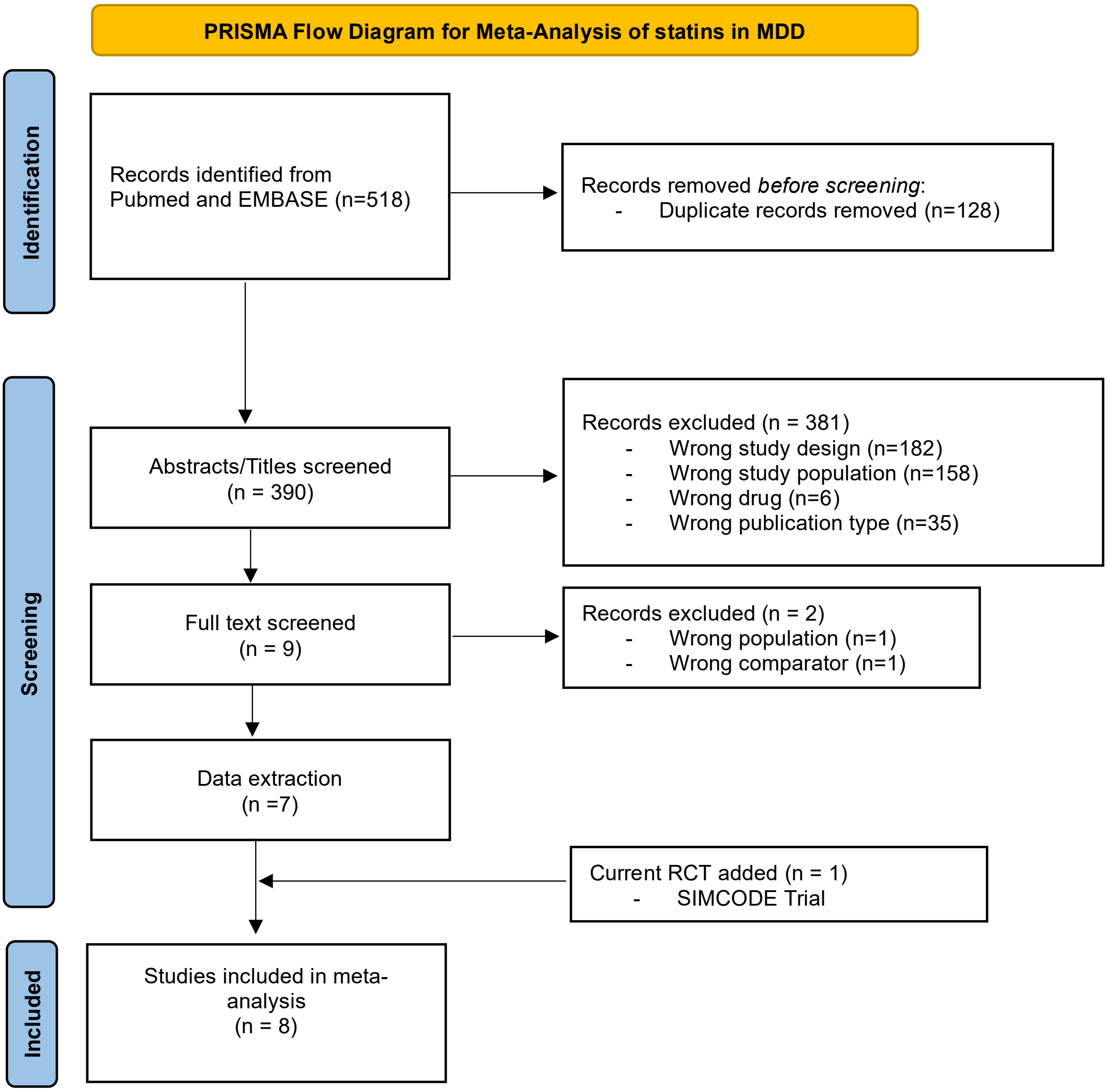

© 2025 Otte C et al. *JAMA Psychiatry*.

eFigure 2A-B: Probability for MADRS response and remission after 12 weeks with corresponding 95%-CI (error bars).

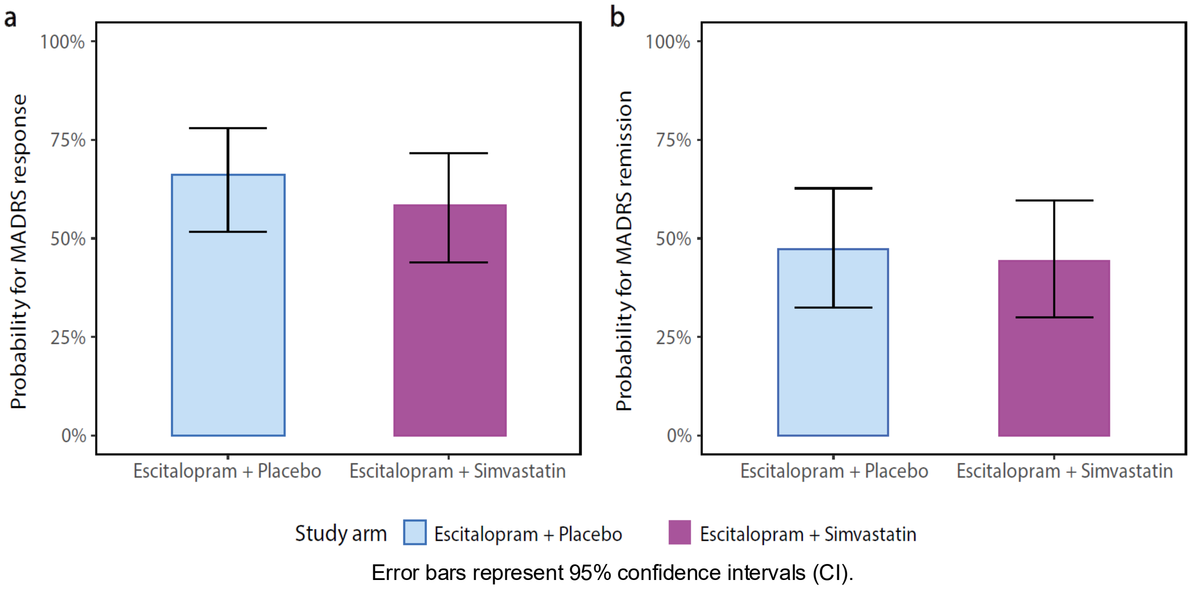

eFigure 3A-C: Prevalence of LDL (> 100 mg/dl), total cholesterol (> 200 mg/dl) and CRP (> 3 mg/l) over a predefined cut-off with corresponding 95%-CI, stratified by visit and treatment group. LDL and total cholesterol were only assessed at baseline and after 12 weeks in order to not compromise blinding.

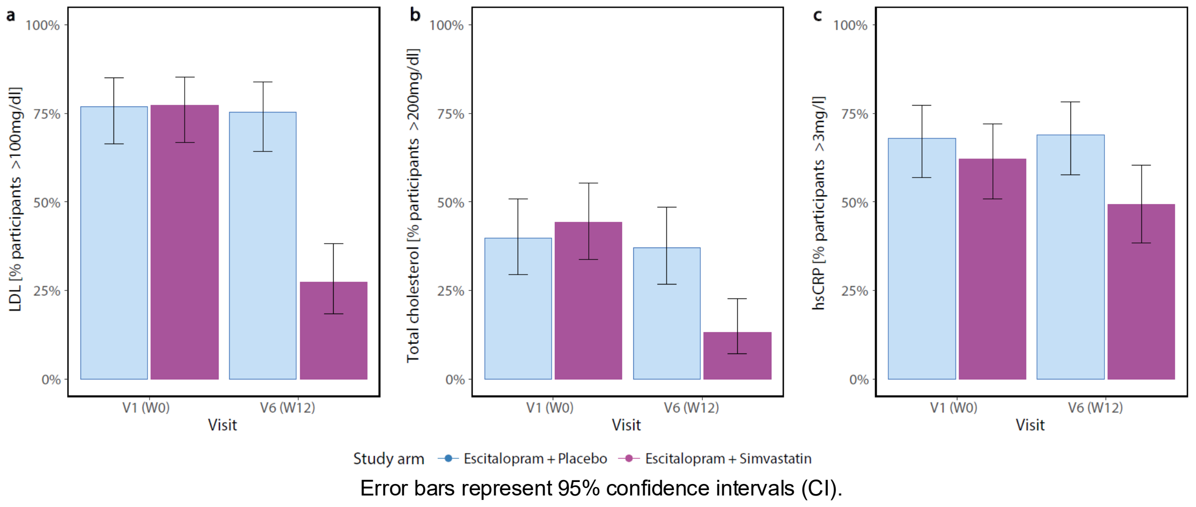

© 2025 Otte C et al. *JAMA Psychiatry*.

eFigure 4: Subgroup analyses.

Subgroup	N	Escitalopram + Placebo	Escitalopram + Simvastatin
**Sex**	160		
Male		16 (20%)	18 (22%)
Female		63 (80%)	63 (78%)
**Severity of obesity**	158		
Class I		19 (25%)	27 (33%)
Class II		25 (32%)	20 (25%)
Class III		33 (43%)	34 (42%)
**Metabolic syndrome**	156		
No		50 (65%)	51 (65%)
Yes		27 (35%)	28 (35%)
**Severity of depression**	156		
Moderate		71 (92%)	73 (92%)
Severe		6 (7.8%)	6 (7.6%)
**Chronicity of depression**	147		
No		56 (79%)	64 (84%)
Yes		15 (21%)	12 (16%)
**Inflammatory status**	157		
≤ 3 mg/L		25 (32%)	30 (38%)
> 3 mg/L		53 (68%)	49 (62%)

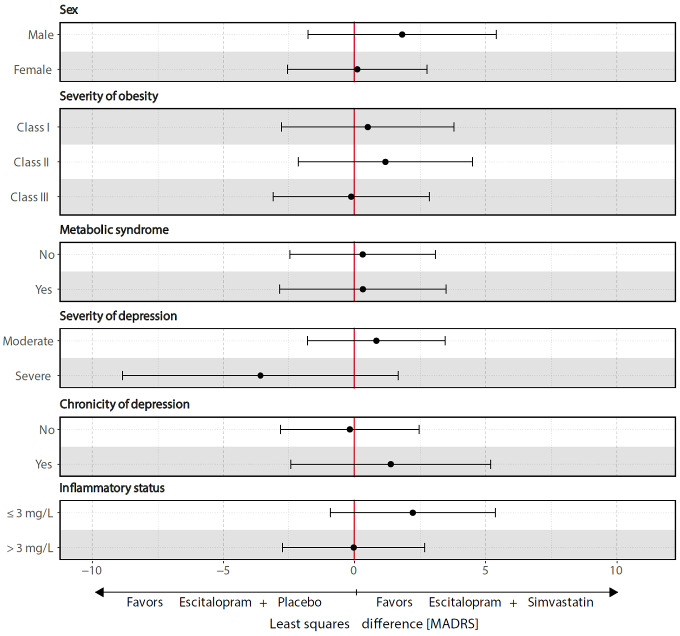

Error bars represent 95% confidence intervals (CI).

eFigure 5: Suicidal symptoms, assessed by MADRS item 10, at screening, baseline and throughout the study by treatment group.

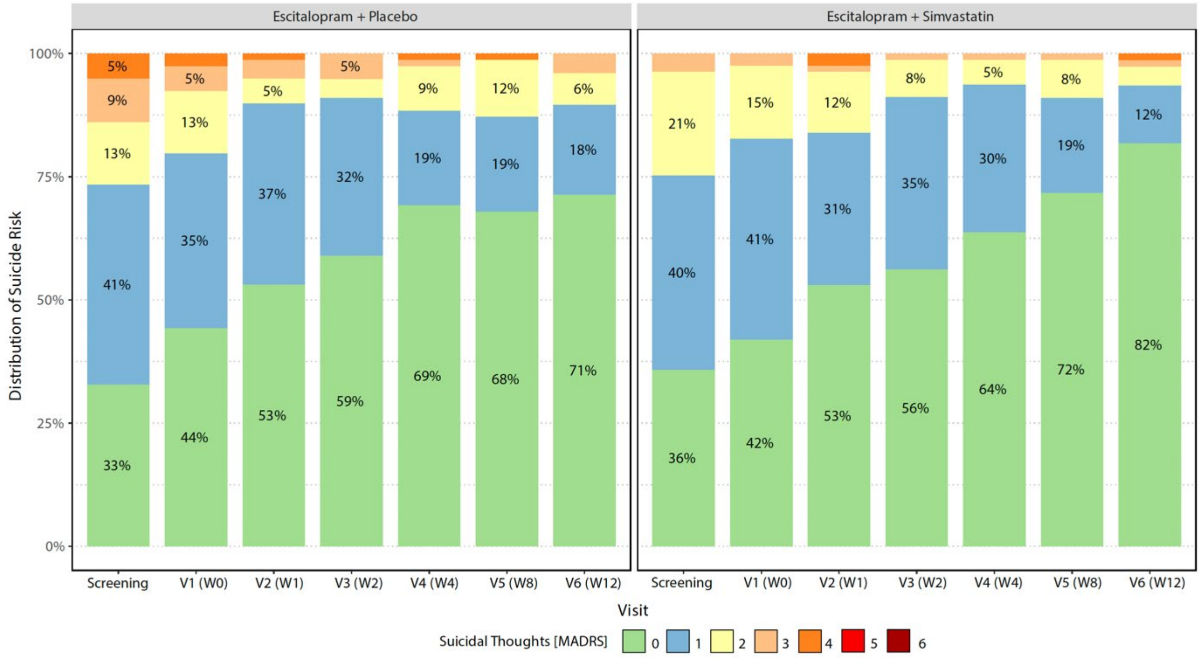

The MADRS item 10 assesses the presence and severity of suicidal symptoms, ranging from no symptoms (score of 0) to the highest severity, including attempted or planned suicide (score of 6). The figure illustrates the distribution of responses for item 10 by treatment group at the screening visit, baseline visit, and all subsequent visits through to the end of the study (week 12). As per the exclusion criteria, none of the participants exhibited high levels of suicidality (MADRS item 10 > 4) at study entry, nor did any participant develop high levels of suicidality during the study.

© 2025 Otte C et al. *JAMA Psychiatry*.

eFigure 6: Meta-analysis of all available evidence from RCTs evaluating the efficacy of statins for depression severity in adults with MDD. (a) Risk-of-bias assessment of the included 8 RCTs (3-9); (b) meta-analysis, stratified by risk-of-bias.

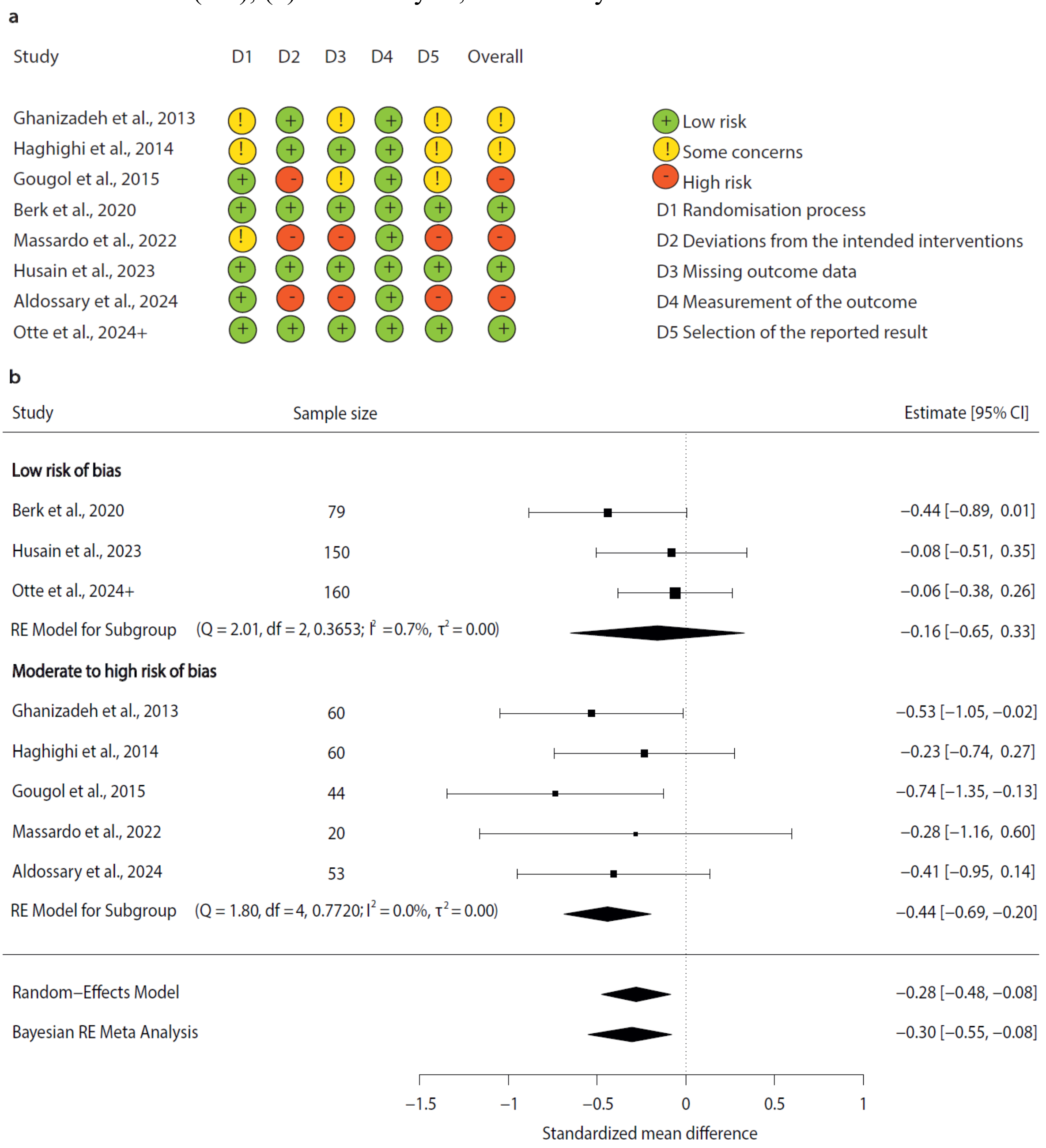

[a]Risk of bias was assessed using the revised Cochrane risk-of-bias tool for randomized trials (RoB 2).

© 2025 Otte C et al. *JAMA Psychiatry*.

eReferences

1. Gold SM, Otte C, Friede T, Brasanac J, Dogan DY, Chae WR. Do statins reduce severity of depressive symptoms in adults with major depressive disorder? 2024; CRD42024588640. Available from: https://www.crd.york.ac.uk/prospero/display_record.php?ID=CRD42024588640.

2. Ewald H, Klerings I, Wagner G, Heise TL, Stratil JM, Lhachimi SK, et al. Searching two or more databases decreased the risk of missing relevant studies: a metaresearch study. J Clin Epidemiol. 2022;149:154-64.

3. Ghanizadeh A, Hedayati A. Augmentation of fluoxetine with lovastatin for treating major depressive disorder, a randomized double-blind placebo controlled-clinical trial. *Depress Anxiety*. 2013;30(11):1084-1088. doi:10.1002/da.22195

4. Haghighi M, Khodakarami S, Jahangard L, et al. In a randomized, double-blind clinical trial, adjuvant atorvastatin improved symptoms of depression and blood lipid values in patients suffering from severe major depressive disorder. *J Psychiatr Res*. 2014;58:109-114. doi:10.1016/j.jpsychires.2014.07.018

5. Gougol A, Zareh-Mohammadi N, Raheb S, et al. Simvastatin as an adjuvant therapy to fluoxetine in patients with moderate to severe major depression: A double-blind placebo-controlled trial. *J Psychopharmacol Oxf Engl*. 2015;29(5):575-581. doi:10.1177/0269881115578160

6. Berk M, Mohebbi M, Dean OM, et al. Youth Depression Alleviation with Anti-inflammatory Agents (YoDA-A): a randomised clinical trial of rosuvastatin and aspirin. *BMC Med*. 2020;18(1):16. doi:10.1186/s12916-019-1475-6

7. Massardo T, Quintana JC, Risco L, et al. Effect of Low-Dose Statins in Addition to Standard Therapy on Brain Perfusion and Neurocognitive Performance in Patients with Major Depressive Disorder. *Neuropsychobiology*. 2022;81(4):271-285. doi:10.1159/000521104

8. Husain MI, Chaudhry IB, Khoso AB, et al. Effect of Adjunctive Simvastatin on Depressive Symptoms Among Adults With Treatment-Resistant Depression: A Randomized Clinical Trial. *JAMA Netw Open*. 2023;6(2):e230147. doi:10.1001/jamanetworkopen.2023.0147

9. Aldossary KM, Ali LS, Abdallah MS, et al. Effect of a high dose atorvastatin as added-on therapy on symptoms and serum AMPK/NLRP3 inflammasome and IL-6/STAT3 axes in patients with major depressive disorder: randomized controlled clinical study. *Front Pharmacol*. 2024;15:1381523. doi:10.3389/fphar.2024.1381523

© 2025 Otte C et al. *JAMA Psychiatry*.
