# Supplementary material for: Simvastatin as Add-On Treatment to Escitalopram in Patients With Major Depression and Obesity: A Randomized Clinical Trial
Source: JAMA Psychiatry. 2025 Jun 4;82(8):759–67. doi: 10.1001/jamapsychiatry.2025.0801 (PMC12138799; doi:10.1001/jamapsychiatry.2025.0801)
Supplement: Supplement 3. — Data Sharing Statement. [file jamapsychiatry-e250801-s003.pdf]

Data Sharing Statement

Otte C, Chae WR, Dogan DY, et al. Simvastatin as add-on treatment to escitalopram in patients with major depression and obesity: a randomized clinical trial. *JAMA Psychiatry*. Published online June 4, 2025. doi:10.1001/jamapsychiatry.2025.0801

Data
Additional Information: Clinicaltrials.gov (NCT04301271), German Clinical Trials Register (DRKS, DRKS00021119) and clinicaltrialsregister.eu (EudraCT 2018-002947-27)

Data available: Yes
Data types: Deidentified participant data
How to access data: Individual patient level data on primary and secondary endpoints are available for download via the Zenodo repository (dot: 10.5281/zenodo.15004761).
When available: beginning date: 30-05-2025

Supporting Documents
Document types: None
Additional Information
Who can access the data: Anyone requesting the data.
Types of analyses: Individual patient level data on primary and secondary endpoints are available for download via the Zenodo repository (dot: 10.5281/zenodo.15004761).
Mechanisms of data availability: Without investigator support.
